# Cloning, analysis and functional annotation of expressed sequence tags from the Earthworm *Eisenia fetida*

**DOI:** 10.1186/1471-2105-8-S7-S7

**Published:** 2007-11-01

**Authors:** Mehdi Pirooznia, Ping Gong, Xin Guan, Laura S Inouye, Kuan Yang, Edward J Perkins, Youping Deng

**Affiliations:** 1 Department of Biological Sciences, University of Southern Mississippi, Hattiesburg, MS, 39406, USA; 2 SpecPro Inc., 3909 Halls Ferry Rd, Vicksburg, MS, 39180, USA; 3 Environmental Laboratory, U.S. Army Engineer Research and Development Center, 3909 Halls Ferry Rd, Vicksburg, MS, 39180, USA

## Abstract

**Background:**

*Eisenia fetida*, commonly known as red wiggler or compost worm, belongs to the Lumbricidae family of the Annelida phylum. Little is known about its genome sequence although it has been extensively used as a test organism in terrestrial ecotoxicology. In order to understand its gene expression response to environmental contaminants, we cloned 4032 cDNAs or expressed sequence tags (ESTs) from two *E. fetida *libraries enriched with genes responsive to ten ordnance related compounds using suppressive subtractive hybridization-PCR.

**Results:**

A total of 3144 good quality ESTs (GenBank dbEST accession number EH669363–EH672369 and EL515444–EL515580) were obtained from the raw clone sequences after cleaning. Clustering analysis yielded 2231 unique sequences including 448 contigs (from 1361 ESTs) and 1783 singletons. Comparative genomic analysis showed that 743 or 33% of the unique sequences shared high similarity with existing genes in the GenBank nr database. Provisional function annotation assigned 830 Gene Ontology terms to 517 unique sequences based on their homology with the annotated genomes of four model organisms *Drosophila melanogaster*, *Mus musculus*, *Saccharomyces cerevisiae*, and *Caenorhabditis elegans*. Seven percent of the unique sequences were further mapped to 99 Kyoto Encyclopedia of Genes and Genomes pathways based on their matching Enzyme Commission numbers. All the information is stored and retrievable at a highly performed, web-based and user-friendly relational database called EST model database or ESTMD version 2.

**Conclusion:**

The ESTMD containing the sequence and annotation information of 4032 *E. fetida *ESTs is publicly accessible at .

## Background

As key representatives of the soil fauna, earthworms are essential in maintaining soil fertility through their burrowing, ingestion and excretion activities [1]. There are over 8000 described species worldwide, existing everywhere but in Polar and arid climates [2]. They are increasingly recognized as indicators of agroecosystem health and ecotoxicological sentinel species because they are constantly exposed to contaminants in soil. The earthworm species (e.g., *Eisenia fetida*, *Eisenia andrei*, and *Lumbricus terrestris*) widely used in standardized acute and reproduction toxicity tests belong to the Lumbricidae family (phylum, Annelida; class, Clitellata; subclass Oligochaeta; order, Haplotaxida; superfamily, Lumbricoidea; family, Lumbricidae). *E. fetida and E. andrei *are two sibling species commonly found in North American composters and are sold commercially for fish bait. They have a life span of 4–5 years and are obligatorily amphimictic even though each worm has both male and female reproductive organs [3].

Like many other ecologically important species, genomics research in earthworms lags far behind other model species such as *Mus musculus *and *Caenorhabditis elegans*. In the absence of full genome sequences, expressed sequence tags (ESTs) allow rapid identification of expressed genes by sequence analysis and are an important resource for comparative and functional genomic studies. ESTs are often generated from either end of randomly selected cDNA clones and provide valuable transcriptional data for the annotation of genomic sequences. Because of recent advances in biotechnology, ESTs are produced daily in large quantities, with nearly 42 million entries in the current GenBank db EST database (release 030207). Nevertheless, it is still a challenging bioinformatics problem to analyze and annotate the often short, redundant and yet error prone EST sequences in an appropriate and efficient manner, especially when the genome sequence of the organism is unknown. Recent years have seen some EST projects undertaken with *L. rubellus *[4] and *E. andrei *[5], which have generated 19,934 and 1,108 ESTs, respectively (db EST release 030207). Before this study, there were only 96 nucleotide and 89 protein Entrez records found for *E. fetida*. In the present study, we cloned, sequenced and analyzed 4032 ESTs from *E. fetida*. We used suppression subtractive hybridization-PCR (SSH) to enrich cDNAs responsive to ten ordnance related compounds (ORCs). This work is part of a larger effort to identify candidate molecular biomarkers for rapid, mechanism-based gene expression assays to supplement current acute and reproductive toxicity tests. The specific objectives of this study were (1) to isolate and characterize cDNAs from *E. fetida *that can be used to monitor exposure to ORCs, and (2) to make the *E. fetida *EST information publicly accessible by integrating it to our web-based EST model organism database (ESTMD) so that it can be shared with interested parties.

## Results

### cDNA library and EST sequence analysis

**Figure 1:**
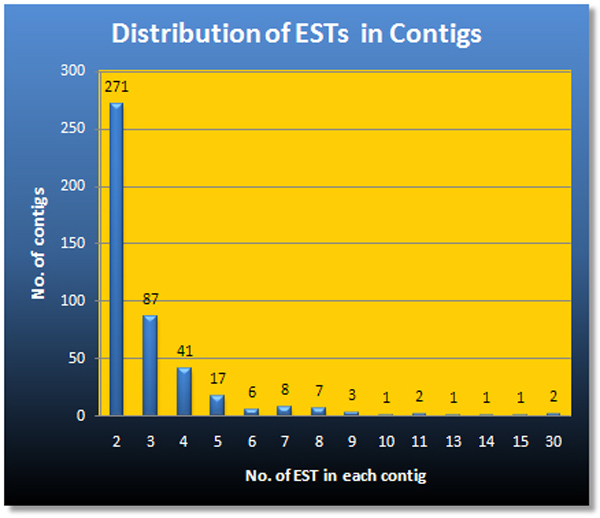
Distribution of 1361 good quality ESTs in 448 assembled contigs.

We cloned a total of 4032 cDNAs from the two SSH libraries (see **Methods **for details). We transformed and picked 2208 clones from forward subtracted cDNA pools and 1824 from the reverse subtracted cDNA pools. After running on 96-well gel electrophoresis, 216 clones were found to be false positives with no inserts or had more than one insert. We sequenced the remaining 3816 clones producing 3144 good quality sequences with an average length of 310 bases. We batch-deposited them in the GenBank db EST under accession numbers EH669363–EH672369 and EL515444–EL515580. Clone sequences that were too short (<50 bases) or of poor quality (<50 good quality bases, see methods for quality criteria) were excluded from further analysis. The observed failure rate (18%) is typical for high-throughput sequencing [6]. The deposited, cleaned sequences were further assembled into 2231 clusters (or unique sequences) on the basis of sequence similarity and quality. Nearly 80% or 1783 of the clusters produced were singletons, and 80% of the remaining 448 contigs (average length = 428 bases) were assembled from 2 or 3 clone sequences (Figure 1). The highest number of sequences assembled into one contig was 30. The most represented putative genes in our libraries are Cd-metallothionein, cytochrome oxidase, chitotriosidase, actin, ATP synthase, Nahoda protein, lysozyme, SCBP (soluble calcium binding protein), ferritin, troponin T, lumbrokinase, and myohemerythrin (Table 1).

**Table 1:** The most represented putative genes in the *Eisenia fetida *cDNA libraries

**Contig**	**ESTs**	**Length**	**Accession Version #**	**bit**	**E-value**	**Identities**	**Organism**	**Description**
Contig423	7	452	AAH69614.1	336	1.00E-30	64/137	Homo sapiens	CHIT1 protein
Contig424	7	480	CAE18118.1	205	2.00E-15	40/58	Lumbricus terrestris	SCBP3 protein
Contig426	7	659	AAW25147.1	171	5.00E-11	42/83	Schistosoma japonicum	SJCHGC00665 protein
Contig427	7	494	CAA48798.1	714	3.00E-74	132/135	Podocoryne carnea	actin
Contig428	7	230	BAC06447.1	195	3.00E-14	37/76	Haemaphysalis longicornis	chitinase
Contig428	7	230	NP_001020370.1	182	1.00E-12	36/73	Homo sapiens	chitinase 3-like 2 isoform c
Contig429	7	439	ABC60436.1	749	2.00E-78	145/146	Hirudo medicinalis	cytoplasmic actin
Contig431	8	397	AAX51817.1	383	5.00E-36	73/100	Diloma arida	actin
Contig434	8	579	CAA65364.1	971	1.00E-104	189/189	Lumbricus terrestris	Actin
Contig435	8	601	AAA96144.1	322	1.00E-28	62/134	Hirudo medicinalis	destabilase I
Contig436	8	810	XP_394202.2	217	3.00E-16	47/162	Apis mellifera	PREDICTED: similar to GA11808-PA
Contig436	8	810	EAL25702.1	216	4.00E-16	51/183	Drosophila pseudoobscura	GA11808-PA
Contig437	8	394	AAX77000.1	552	1.00E-55	110/122	Metaphire feijani	cytochrome c oxidase subunit 1
Contig438	9	1055	EAR81082.1	127	1.00E-05	27/60	Tetrahymena thermophila	hypothetical protein TTHERM_02141640
Contig440	9	472	NP_008244.1	439	2.00E-42	96/152	Lumbricus terrestris	ATP6_10599 ATP synthase F0 subunit 6
Contig442	11	449	CAA65364.1	760	1.00E-79	147/147	Lumbricus terrestris	Actin
Contig443	11	846	NP_008239.1	256	1.00E-20	57/105	Lumbricus terrestris	COX2_10599 cytochrome c oxidase subunit II
Contig444	13	894	AAH69614.1	614	4.00E-62	128/294	Homo sapiens	CHIT1 protein
Contig446	15	584	AAX62723.1	576	4.00E-58	122/166	Eisenia fetida	cytochrome oxidase subunit I
Contig448	30	488	CAA15423.1	246	5.00E-20	40/41	Eisenia fetida	metallothionein

### Comparative sequence analysis

We used the 2,231 unique sequences to search non-redundant protein databases using blastx [6-8]. A total of 743 sequences (33% of all unique sequences) matched known proteins with cut-off expect (*E*) values of 10^-5 ^or lower, among which 71 (3%) had *E*-values between 10^-100 ^and 10^-50^, 309 (14%) between 10^-50 ^and 10^-20^, and 363 (16%) between 10^-20 ^and 10^-5 ^(Table 2). A total of 880 unique sequences had less meaningful matches (*E *> 10^-5^). The remaining 608 sequences (27%) had no matches. We also examined unique *E. fetida *sequences to determine similarity to the genes of four model organisms *Drosophila melanogaster*, *Mus musculus*, *Saccharomyces cerevisiae*, and *Caenorhabditis elegans*. A total of 830 blastx matches were found for 517 *E. fetida *unique sequences (23%) at the cut-off *E*-value of 10^-5 ^(Table 3). Some *E. fetida *ESTs matched genes conserved between the four organisms. More than 50% of the matches came from the mouse genome, whereas only 5 matches were found in the yeast genome. These results suggest that earthworms may be more evolutionarily distant from the yeast than from the other three organisms.

**Table 2:** Homology analysis of the 2231 unique *Eisenia fetida *EST sequences based on the results from BLASTX against NCBI's nr database

	Contig		Singleton		Total	
**Homology**	**N**	**%**	**N**	**%**	**N**	**%**

10^-150 ^< E ≤ 10^-100^	0	0	0	0	0	0
10^-100 ^< E ≤ 10^-50^	38	8	33	2	71	3
10^-50 ^< E ≤ 10^-20^	93	21	216	12	309	14
10^-20 ^< E ≤ 10^-5^	78	17	285	16	363	16
**Total meaningful match (E ≤ 10^-5^)**	**209**	**46**	**534**	**30**	**743**	**33**
**Less meaningful match (E > 10^-5^)**	**165**	**37**	**715**	**40**	**880**	**40**
**No match (No hit)**	**74**	**17**	**534**	**30**	**608**	**27**
Total	448	100	1783	100	2231	100

**Table 3:** Comparison of significant homologous matches (*E *≤ 10^-5^) to four model organisms of the 2231 unique *Eisenia fetida *EST sequences. The full listing of matches is available in Additional file 1.

**Organism Name**	**Number of matches**	**% of unique sequences**
Drosophila melanogaster	265	12%
Mus musculus	447	20%
Saccharomyces cerevisiae	5	0.2%
Caenorhabditis elegans	113	5%
**Total matches**	**830**	
**Total unique sequences**	**517**	**23%**

### Functional classification

We adopted the Gene Ontology (GO) annotation of the aforesaid four model organisms to interpret the function of the *E. fetida *ESTs [6-8]. Each unique sequence of *E. fetida *was assigned the same gene functions of the best blastx hit genes (*E *≤ 10^-5^) in these model organisms' genome. The assigned GO terms for the unique sequences are categorized and outlined in Table 4 (biological process), Table 5 (molecular function), and Table 6 (cellular component). A complete listing of all the GO mappings is available in Additional file 1. The most represented molecular function is "binding" accounting for 51% of the total 517 unique sequences assigned with at least one GO term (Table 5), whereas those for biological processes are "cellular process" (39%) and "physiological process" (40%) (Table 4). In terms of the final child GO categories, the most frequently assigned biological processes are "protein metabolism" (12.5%), "cellular macromolecule metabolism" (11.7%), and "cellular protein metabolism" (11%) under both cellular and physiological processes (Table 4), whereas those for molecular functions are "hydrolase activity" (11%) and "protein binding" (10%) (Table 5). The largest subcategory in cellular components is "intracellular organelle" (23.6%) under both the intracellular part and the organelle (Table 6).

**Table 4:** Distribution of Gene Ontology biological process terms assigned to *Eisenia fetida *unique sequences on the basis of their homology to the annotated genome of four model organisms. The number of total matches is 830 as shown in Table 3 and the full listing is available in Additional file 2.

Gene Ontology term	Unique sequences	Percentage of total matches
cellular process	328	39.52%
cell communication	52	6.27%
cellular physiological process	309	37.23%
cell organization and biogenesis	62	7.47%
cellular metabolism	255	30.72%
cellular biosynthesis	46	5.54%
cellular macromolecule metabolism	97	11.69%
cellular protein metabolism	92	11.08%
regulation of cellular physiological process	48	5.78%
transport	71	8.55%
regulation of cellular process	51	6.14%
development	51	6.14%
physiological process	331	39.88%
cellular physiological process	309	37.23%
cell organization and biogenesis	62	7.47%
cellular metabolism	255	30.72%
cellular macromolecule metabolism	97	11.69%
localization	53	6.39%
metabolism	272	32.77%
biosynthesis	70	8.43%
cellular metabolism	255	30.72%
cellular biosynthesis	46	5.54%
cellular macromolecule metabolism	97	11.69%
cellular protein metabolism	92	11.08%
organic acid metabolism	10	1.20%
macromolecule metabolism	181	21.81%
biopolymer metabolism	58	6.99%
cellular macromolecule metabolism	97	11.69%
macromolecule biosynthesis	34	4.10%
protein metabolism	96	11.57%
primary metabolism	164	19.76%
protein metabolism	104	12.53%
regulation of physiological process	51	6.14%
regulation of biological process	57	6.87%
response to stimulus	47	5.66%

**Table 5:** Distribution of Gene Ontology molecular function terms assigned to *Eisenia fetida *unique sequences on the basis of their homology to the annotated genome of four model organisms. The number of total matches is 830 as shown in Table 3 and the full listing is available in Additional file 2.

**Gene Ontology term**	**Unique sequences**	**Percentage of total matches**
antioxidant activity	2	0.24%
binding	426	51.33%
carbohydrate binding	18	2.17%
cofactor binding	6	0.72%
ion binding	84	10.12%
lipid binding	5	0.60%
metal cluster binding	3	0.36%
neurotransmitter binding	3	0.36%
nucleic acid binding	53	6.39%
nucleotide binding	68	8.19%
pattern binding	10	1.20%
peptide binding	4	0.48%
protein binding	90	10.84%
tetrapyrrole binding	5	0.60%
vitamin binding	2	0.24%
catalytic activity	194	23.37%
helicase activity	4	0.48%
hydrolase activity	94	11.33%
isomerase activity	8	0.96%
ligase activity	7	0.84%
lyase activity	11	1.33%
oxidoreductase activity	46	5.54%
small protein activating enzyme activity	3	0.36%
transferase activity	27	3.25%
enzyme regulator activity	16	1.93%
motor activity	4	0.48%
nutrient reservoir activity	2	0.24%
signal transducer activity	26	3.13%
structural molecule activity	47	5.66%
transcription regulator activity	16	1.93%
translation regulator activity	13	1.57%
transporter activity	33	3.98%

**Table 6:** Distribution of Gene Ontology cellular component terms assigned to *Eisenia fetida *unique sequences on the basis of their homology to the annotated genome of four model organisms. The number of total matches is 830 as shown in Table 3 and the full listing is available in Additional file 2.

**Gene Ontology term**	**Unique sequences**	**Percentage of total matches**
cell part	280	33.73%
intracellular part	224	26.99%
calcineurin complex	2	0.24%
cytoplasm	152	18.31%
cytoplasmic part	132	15.90%
intracellular organelle	196	23.61%
intracellular organelle part	97	11.69%
proteasome complex (sensu Eukaryota)	10	1.20%
proteasome regulatory particle (sensu Eukaryota)	8	0.96%
proton-transporting ATP synthase complex	4	0.48%
respiratory chain complex I	3	0.36%
respiratory chain complex III	3	0.36%
respiratory chain complex IV	1	0.12%
ribonucleoprotein complex	35	4.22%
RNA polymerase complex	2	0.24%
RNAi effector complex(1)	1	0.12%
ubiquinol-cytochrome-c reductase complex	3	0.36%
membrane	107	12.89%
membrane part	81	9.76%
protein serine/threonine phosphatase complex	2	0.24%
envelope	33	3.98%
extracellular matrix	10	1.20%
extracellular matrix part	6	0.72%
extracellular region	51	6.14%
extracellular region part	40	4.82%
membrane-enclosed lumen	2	0.24%
organelle	196	23.61%
intracellular organelle	196	23.61%
membrane-bound organelle	148	17.83%
non-membrane-bound organelle	68	8.19%
organelle part	97	11.69%
vesicle	8	0.96%
organelle part	97	11.69%
protein complex	102	12.29%
synapse	7	0.84%
synapse part	3	0.36%

### Pathway assignment

We assigned the unique *E. fetida *sequences to a specific Kyoto Encyclopedia of Genes and Genomes (KEGG) pathway based on their matching Enzyme Commission (EC) numbers. A total of 157 unique sequences (accounting for 7% of all unique sequences) including 28 contigs and 129 singletons matched enzymes with an EC number. Fifty-eight unique sequences are involved in two or more pathways. The remaining 99 pathway-assigned sequences are mapped to only one pathway. Eighty-two unique sequences (52% of total) containing 14 contigs and 68 singletons were assigned to metabolism pathways (Table 7 and complete listing available in Additional file 2). Amino acid metabolism has the highest number of assigned pathways, followed by carbohydrate metabolism, energy metabolism, translation, and signal transduction. Genes putatively coded by a singleton EW1_F1plate05_B07 (enoyl coenzyme A hydratase) and Contig 251 (thioredoxin peroxidase) are most versatile, which are mapped to 10 and 8 pathways, respectively.

**Table 7:** KEGG pathway mapping for *Eisenia fetida *unique sequences. The total number of mapped unique sequences is 157. The full listing of pathways is available in Additional file 3.

**KEGG pathway**	**No. of unique sequence**	Percentage of total unique sequences*	**No. of KEGG pathways mapped**
**Metabolism**	**82**	**52%**	**57**
Carbohydrate Metabolism	35	22%	10
Energy Metabolism	28	18%	8
Nucleotide Metabolism	2	1%	2
Amino Acid Metabolism	18	11%	12
Metabolism of Other Amino Acids	10	6%	3
Glycan Biosynthesis and Metabolism	6	4%	8
Metabolism of Cofactors and Vitamins	9	6%	6
Biosynthesis of Secondary Metabolites	2	1%	1
Xenobiotics Biodegradation and Metabolism	6	4%	7
**Genetic Information Processing**	**28**	**18%**	**6**
Transcription	2	1%	2
Translation	17	11%	1
Folding, Sorting and Degradation	9	6%	3
**Environmental Information Processing**	**27**	**17%**	**10**
Membrane Transport	1	1%	1
Signal Transduction	14	9%	6
Signaling Molecules and Interaction	13	8%	3
**Cellular Processes**	**37**	**24%**	**18**
Cell Motility	9	6%	3
Cell Communication	13	8%	4
Endocrine System	4	3%	3
Immune System	5	3%	3
Nervous System	8	5%	2
Sensory System	3	2%	1
Development	3	2%	2
**Human Diseases**	**9**	**6%**	**8**
Neurodegenerative Disorders	6	4%	4
Metabolic Disorders	2	1%	2
Cancers	2	1%	2

### ESTMD (EST Model Database) web application

The ESTMD is a highly performed, web-accessible and user-friendly relational database [6]. It facilitates and enhances the retrieval and analysis of EST information by providing a number of comprehensive tools for mining raw, cleaned and clustered EST sequences, GO terms and KEGG pathway information as well as a variety of web-based services such as BLAST search, data submission and sequence download. The application is developed using advanced Java technology (Jsp and Servlets) and it supports portability, extensibility and data recovery. It can be accessed at . The workflow process is as follows: Users input keywords or IDs from the web interface and then submit them as a query to the server. The server processes the query and retrieves date from the backend database through the database connection interface. The results are processed and sent to the users in proper formats.

**Figure 2:**
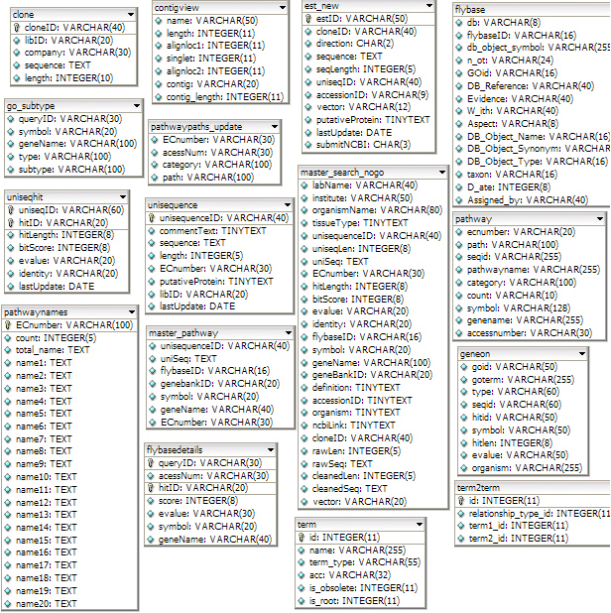
The ESTMD database schema showing tables, fields, and data types.

The main ESTMD tables are clone, contigview, est new, flybase, geneon, gomodels, pathway, term, uniseqhit, master_search and unisequence (Figure 2). Main sequence information including ECnumber, Labname, raw and clean sequence, and vector information are stored in the master_search table.

## Discussion

**Figure 3:**
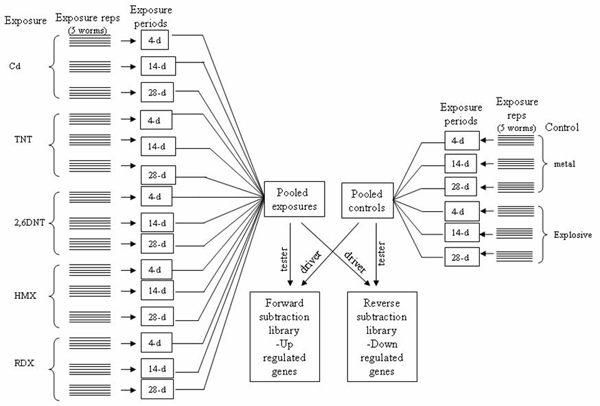
Scheme of RNA sample pooling for subtractive suppression hybridization cDNA library construction: the first library.

**Figure 4:**
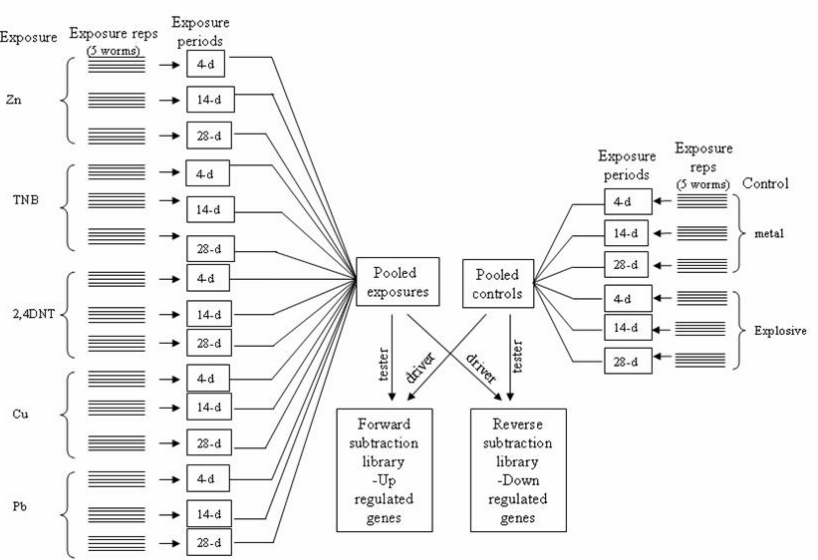
Scheme of RNA sample pooling for subtractive suppression hybridization cDNA library construction: the second library.

Using SSH-PCR we enriched earthworm cDNAs responsive to exposure of ten ORCs that represent three classes of chemicals, i.e., nitroaromatics (2,4-dinitrotoluene, 2,6-dinitrotoluene, 2,4,6-trinitrotoluene (TNT), and trinitrobenzene), heterocyclic nitroamines (1,3,5-trinitroperhydro-1,3,5-triazine or RDX and 1,3,5,7-tetranitro-1,3,5,7-tetrazocane or HMX) and heavy metals (Cd, Cu, Zn and Pb) (Figures 3 and 4). Exposure times varied from 4-d to 28-d to capture gene expression changes at different time points. In consideration of the magnitude of effort required by this study, we selected a single dose for each compound. We also believe that differentially expressed transcripts captured on the time scale may represent to a certain degree those manifested on the dosage scale, and vise versa. We chose time over dosage mainly because we are more interested in early indication of later effects. We purposely mixed the RNA samples from different exposures for library construction because of the large variety of chemicals and exposure length, compared to the relatively small amount of resources available. The cloned cDNAs may not represent genes responding to one specific compound because each chemical, especially each class of chemicals, is likely to have specific mode of action involving different genes. Nevertheless, this library construction strategy served our downstream purpose of making cDNA microarrays with the isolated cDNA clones even though we cannot identify which cDNA or groups of cDNAs responded to which compound and at which exposure time point using the raw EST data. The combination of SSH-PCR and cDNA microarray analysis has been a widely used approach for identifying differentially expressed genes [9,10] and characterizing mechanisms of action of known and suspected toxicants [11,12], especially when there is no or little genomic information available for the test organism. Our microarray studies have generated data enabling us to further identify differentially expressed transcripts and to elucidate sublethal toxicological mechanisms in *E. fetida *exposed to TNT alone [13] or a mixture of TNT and RDX [14].

It is worth noting that the comparative sequence analysis (23%) and functional classification (7%) based on GO and KEGG analysis only found a small portion of the ESTs highly homologous (*E *≤ 10^-5^) with well-annotated genes. Nevertheless, the functions of these ESTs are widely distributed representing 830 different GO terms and 99 different KEGG pathways. Notably, genes putatively involved in carbohydrate, energy and amino acid metabolism, cellular processes of endocrine, immune, nervous and sensory systems, signal transduction, DNA transcription, RNA translation and post-translation splicing are identified suggesting that the ten ORCs may have affected a wide range of important pathways.

From candidate biomarker gene point of view, we found repeatedly the existence of some toxicant-specific *E. fetida *mRNAs in our libraries (Table 1 and Additional file 3). For instance, the expression of metallothionein (MT) mRNA, the most abundant transcript in our cDNA libraries, is reportedly a sensitive and early genetic biomarker of metal exposure [15-18]. Demuynck et al. [15] demonstrated that a single exposure to 8 mg Cd/kg of dry soil for 1 day induced MT mRNA. Brulle et al. [16] observed changes in MT mRNA expression as early as 14 hr after exposure. There are also clear differences of MT gene expression between worms exposed to different Cd concentrations (8, 80 or 800 mg Cd/kg of dry soil) [15]. Copper is an essential element for the activity of a number of physiologically important enzymes including cytochrome c oxidase (COX), Cu/Zn-superoxide dismutase (SOD), and dopamine-beta-hydroxylase (DBH). However, exposure to a toxic level of copper can not only induce MT for Cu sequestration [17] but also alter the expression of COX (Table 1), SOD and DBH genes (Additional file 3). Further research is required to establish dose-dependent gene expression in both laboratory and field conditions.

## Conclusion

This study presented a framework for cloning, analyzing and annotating differentially expressed ESTs from the oligochaete *E. fetida*. A total of 2231 unique sequences were clustered from 3144 good quality clones, among which 743 (33%) share high similarity with existing genes in the GenBank nr database. We assigned 830 GO terms to 517 unique sequences based on their homology with the annotated genomes of four model organisms *Drosophila melanogaster*, *Mus musculus*, *Saccharomyces cerevisiae*, and *Caenorhabditis elegans*. Seven percent of the unique sequences were further mapped to 99 KEGG pathways. All the sequence and annotation information is accessible at .

## Methods

### cDNA library construction

Two earthworm cDNA libraries were constructed using SSH-PCR [19]. Earthworms (*E. fetida*) were maintained in continuous culture from stocks obtained from Carolina Biological Supply (Burlington, NC). Worms were kept in moistened sphagnum peat (pH 6.5–7.5, moisture content 50%), and were fed ad libitum on a diet of Magic Worm Food (Carolina Biological Supply). Fully clitellate adults weighing 0.3–0.6 g (live weight) were selected for all experiments.

The first SSH library (Figure 3) was made using pooled mRNA (10 μg) extracted from control unexposed worms against worms exposed to Cd (2.6 mmol/kg or 292 mg/kg), TNT (100 mg/kg), 2,6-DNT (54 mg/kg), RDX (50 mg/kg), or HMX (10 mg/kg). For the construction of the second library (Figure 4), mRNA (10 μg) from worms exposed to Cu (293 mg/kg), Pb (8778 mg/kg), Zn (357 mg/kg), 2,4-DNT (100 mg/kg), and TNB (100 mg/kg) was run against mRNA from another set of control worms. Exposures (4-, 14-, or 28-d) were conducted in an Organization for Economic Cooperation and Development (OECD) artificial soil consisting of 70% sand, 20% kaolin clay, and 10% 2-mm sieved peat moss with an adjusted pH between 6.5 and 7.0. Chemical concentrations were selected at effective concentrations for 50% (EC_50_) reduction in fecundity on the basis of our previous studies as well as published literature.

**Figure 5:**
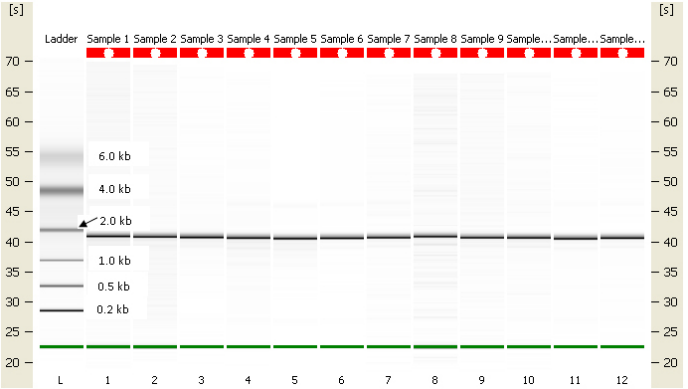
Earthworm total RNA electrophoresis using Agilent 2100 Bioanalyzer.

**Figure 6:**
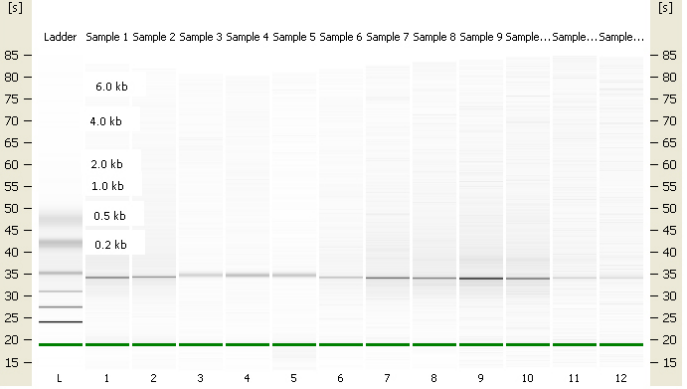
Earthworm purified mRNA electrophoresis using Agilent 2100 Bioanalyzer.

**Figure 7:**
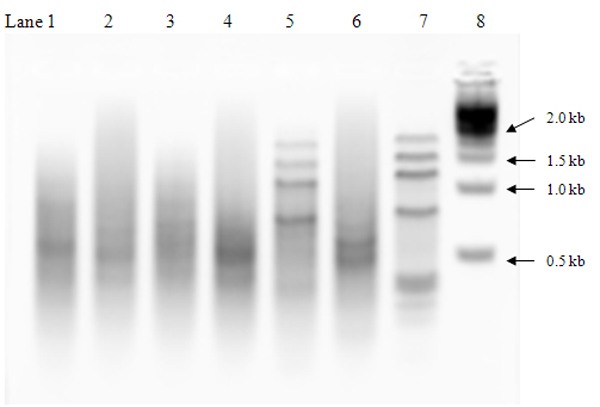
Subtracted and non-subtracted cDNAs electrophoresed on a 2% agarose/SybrGreen gel in 1× sodium borate buffer. Lane 1: forward subtracted earthworm (EW) cDNA; Lane 2: forward non-subtracted EW cDNA; Lane 3: reverse subtracted EW cDNA; Lane 4: reverse non-subtracted EW cDNA; Lane 5: subtracted human skeleton muscle (HSM) cDNA; Lane 6: non-subtracted HSM cDNA; Lane 7: control subtracted human skeleton muscle cDNA; Lane 8: 1 kb DNA ladder.

Exposed and unexposed earthworms were fixed in RNAlater (Ambion, Austin, TX) and stored at -80°C. Total RNA was extracted using RNeasy kits (Qiagen, Valencia, CA), and poly(A) mRNA was separated from total RNA using NucleoTrap mRNA purification kit (BD Biosciences, San Jose, CA). The integrity and concentration of both total and mRNA were checked on an Agilent 2100 Bioanalyzer (Palo Alto, CA). The gel-like images generated by the Bioanalyzer show that both RNAs have only one bright band close to the 2 kb ladder band (Figures 5 &6), which is distinctive from the two bands seen with 18S and 26S RNA of mammalian RNA. A Clontech PCR-Select^™ ^cDNA subtraction kit (BD Biosciences) was then used to enrich for differentially expressed genes (Figure 7).

### EST cloning and sequencing

After the secondary PCR amplification, both forward and reverse subtracted PCR products of the two libraries were cloned using pCR2.1 or pCR4.0 vectors and Mach1-T1 chemically competent cells (Invitrogen, Carlsbad, CA). Positive colonies were picked and grown overnight at 37°C in LB media containing 50 μg/mL ampicillin in a 96-deep well block format. Half of the clone culture (300 μl) was archived with 300 μl of 60% glycerol and stored at -80°C. Two μl of the remaining clone culture was amplified in a 100-μl PCR reaction. After amplification, 8 μl of the PCR reaction was checked on a 96-well electrophoresis gel (2% agarose) for inserts of 100–2000 bps. Amplicons (cDNA inserts) were purified using Millipore Montage PCR 96 Cleanup Kit (Billerica, MA). We checked the concentration of randomly selected purified cDNA using PicoGreen (Molecular Probes, Eugene, OR), which ranged from 100–500 ng/μl with an average of 240 ng/μl. Four μl of the purified cDNA (55 μl in total) was sequenced using BigDye^® ^Terminator v3.1 and a 16-capillary ABI PRISM^® ^3100 Genetic Analyzer (Applied Biosystems, Foster City, CA) according to manufacturer's instruction.

### EST processing

Many software programs are available that provide sequence cleansing and assembly. These include commercial software such as Sequencher (Gene Codes, Ann Arbor, Michigan, USA), and Aligner (CodonCode, Dedham, MA, USA), and open source software such as CAP and TIGR Assembler. With these software packages it is possible to quickly remove vector sequences from each EST clone and screen the ESTs for low-quality sequences. The high-quality and trimmed EST sequences then can be used to find overlap assembly of contiguous sequences. Sequence information was stored in ABI chromatograph trace files, and Phred was used to perform base-calling [20]. Phred read DNA trace data, called bases, assigned quality values to the bases, and wrote the base calls and quality values to output sequence files in either FASTA or SCF format. Quality values for the bases were later used by the sequence assembly program, Phrap [21], to increase the accuracy of assembled sequences. Phred uses simple Fourier methods [22] to examine the four base traces in the data set to predict a series of evenly spaced locations. It determines where the true peak location would be if there were no compressions, dropouts, or other factors shifting the peaks from their locations. Then Phred examines each trace to find the centers of the observed peaks and the areas of these peaks relative to their neighbors. A dynamic programming algorithm [23] is used to match the observed peaks detected in the second step with the predicted peak locations found in the first step. It uses a quality value lookup table to assign the corresponding quality value. The quality value is related to the base call error probability by the formula *QV *= -10 × log_10_(*P*_*e*_) where *P*_*e *_is the probability that the base call is an error [20].

Typically, sequence chromatograms have low-quality regions at the beginning and the end of each sequence read [24]. One can automatically remove the low-quality ends when quality values are available. This process is called "end clipping" or "end trimming". There are two different end clipping methods [24], (1) maximizing regions with error rates below a given threshold, and (2) using separate criteria at the start and the end of the sequence. We chose the former method which was implemented in CodonCode Aligner [25] to remove low quality bases at both ends by setting quality score *QV *≥ 20 (or *P*_*e *_≤ 0.01). Flanking vector/adaptor sequences should also be trimmed off because they can lead to incorrect assemblies or alignment. We input a custom-made vector/adaptor file [24] into the Aligner [25] to trim vector/adaptor sequences. Furthermore, we used VecScreen [26] to detect and then manually removed any residual and partial vector contamination in our ESTs.

Phrap was used to assemble sequence fragments into a larger sequence by identifying overlaps between sample sequences [27]. Samples that can be joined together are put into "contigs". The following greedy algorithm is used in Phrap. First, it finds potential overlaps between samples by looking for shared 12-nucleotide "words" in the sequence. Then the pair of samples with highest number of shared words is found. If the alignment is good enough, it would be kept as a new contig, and the consensus sequence would be calculated; otherwise, the alignment would be rejected, and the two samples would be left separated. Four criteria were used to determine whether to accept or reject an alignment: (1) minimum percent identity (the minimum percentage of identical bases in the aligned region) ≥70%; (2) minimum overlap length ≥25 bps, (3) minimum alignment score; which is similar to (2) but takes any mismatches into account, ≥20 bps; and (4) maximum gap size ≤15 bps. Overall, these criteria were relatively relaxed if compared to more stringent settings such as 90% for minimum percentage identity or minimum overlap length ≥35 bps. If one sample has an insertion/deletion that is larger than 15 bps, the alignment will typically stop there, and the rest of the sample will be considered unaligned. The alignment process would be then repeated. If a sample is in a contig, the consensus sequence is then used for the contig. If the two samples are already in the same contig, the next pair is retrieved and analyzed. It repeats and continues the pairwise joins until all possible joins have been tried, or until the maximum number of merge failures in a row has occurred.

After assembly, all contigs with more than three ESTs were assessed for missassemblies using the assembly viewer Consed [28]. Contigs flagged for possible missassemblies were manually edited using Consed tools to remove potential chimeric ESTs or other suspect ESTs. Chimerism occurs because of multiple insert cloning or mistracking of sequence gel lanes. After assembly with Phrap, contigs with more than three ESTs were examined again in Consed to eliminate additional missassemblies not resolved by Phrap. Any bps with a calculated quality value below 12 was changed to an N (unknown base) which was considered as a suspect ESTs.

### EST comparative analysis and functional assignment

Comparative analysis was performed using blastx through NCBI with the unique sequences (including the consensus sequences of assembled contigs and the singletons). Blastx searches were conducted on our local BLAST server against the NCBI's non-redundant peptide sequence database. The returned search results (100 best hits) were transferred automatically into a relational database. We discarded hits with an *E*-value > 10^-5 ^and sorted out the remaining hits by organism name. To assign putative functions to the unique *E. fetida *sequences, we extracted the GO hierarchical terms of their homologous genes from the protein databases of the following four model organisms: *Mus musculus*, *Drosophila melanogaster*, *Caenorhabditis elegans*, and *Saccharomyces cerevisiae *[29-31]. Meanwhile, we also mapped the unique sequences to metabolic pathways in accordance with the KEGG [32]. Enzyme commission (EC) numbers [33] were acquired for the unique sequences by blastx searching (*E*-value ≤ 10^-5^) the SWIR database, which is made up from three protein databases WormPep, SwissProt and Trembl. The EC numbers were then used to putatively map unique sequences to specific biochemical pathways [6,7]. All the matched GO and pathway information was automatically stored in our local relational database.

### EST database implementation and web application

To facilitate efficient management and retrieval of the EST information obtained from this project, we upgraded our previous developed EST model database (ESTMD version 1) [6] and integrated the earthworm EST information into the new version of ESTMD. The current implementation of ESTMD (version 2) has many new features. The main changes include further normalization of tables from 50 tables to 17, altering main tables to be capable of storing multiple organism information, adding a new table (contigview) to store view information, using a 2D Java class for displaying contigs instead of a Perl script, and implementing the whole web application as a unified portable web module.

**Figure 8:**
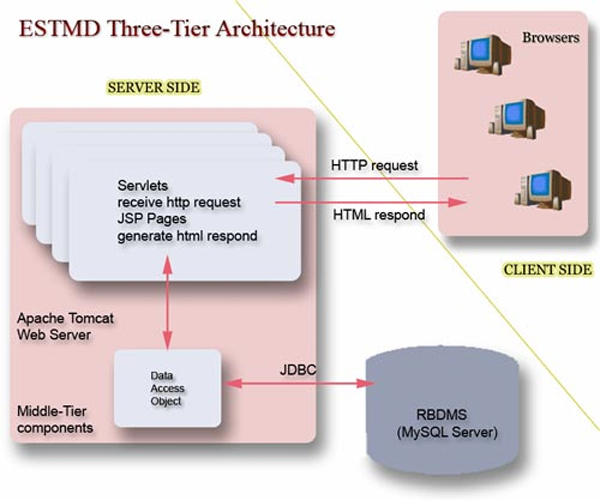
The architecture of ESTMD web application and database.

ESTMD is currently hosted on Suse Linux 10 and can be implemented in MySQL 4.0 or higher version. It has an integrated web-based application with a three-tier structure, i.e., client, sever and backend database (Figure 8). The web-based interface of the database was created using HTML and JavaScript to evaluate the validation of the input on the client side and to reduce the burden on the server side. Apache 2.2 is used as the HTTP web server, while Tomcat 5.5 is the Servlets container. Both of these programs were developed and maintained on Linux and WinNT, ensuring that the database is transplantable and platform-independent. The server-side programs are implemented by Java 2 Enterprise Edition (J2EE) technologies. Servlet and JSP (JavaServer Pages) are used to communicate between users and databases and to implement a query.

## Abbreviations

**COX **Cytochrome c Oxidase

**DBH **Dopamine-Beta-Hydroxylase

**DNT (2,4-DNT) **2,4-dinitrotoluene

**DNT (2,6-DNT) **2,6-dinitrotoluene

**EST **Expressed Sequence Tags

**ESTMD **Expressed Sequence Tags Model Database

**GO **Gene Ontology

**HMX **octahydro-1,3,5,7-tetranitro-1,3,5,7-tetrazocine

**J2EE **Java 2 Enterprise Edition

**JSP **JavaServer Pages

**KEGG **Kyoto Encyclopedia of Genes and Genomes

**ORCs **Ordnance Related Compounds

**RDX **1,3,5-trinitro-1,3,5-triazacyclohexane

**SSH **Suppression Subtractive Hybridization

**SOD **Cu/Zn-superoxide Dismutase

**TNB **1,3,5-trinitrobenzene

**TNT **2,4,6-trinitrotoluene

## Competing interests

The authors declare that they have no competing interests.

## Authors' contributions

YD, PG and EJP initiated the study. LSI performed worm exposure. XG and PG conducted RNA isolation, cDNA cloning and sequencing. MP, YD and PG designed the framework for data analysis and interpretation of data. MP and YD designed and implemented cleansing and assembling process, blast extraction, gene ontology, and pathway analysis. KY implemented a local blast for EST data analysis and participated in cleansing and assembling process. MP and YD designed and implemented the database, normalized tables, data management, web server configuration and web application programming. MP and PG drafted the original manuscript. YD, PG and EJP coordinated and directed the project. All authors have read and approved the final manuscript.

## Supplementary Material

Additional file 1 A complete listing of significant blastx hits (E ≤ 10-5) of the 2231 unique Eisenia fetida EST sequences matching four model organisms *Mus musculus, Drosophila melanogaster, Caenorhabditis elegans, and Saccharomyces cerevisiae.* Click here for file

Additional file 2 A complete listing of 5129 GO terms for the 517 unique *Eisenia fetida* sequences with significant homology with 830 genes of the four model organisms. Click here for file

Additional file 3 A complete listing of the KEGG pathways mapped for 157 unique *Eisenia fetida* sequences. Click here for file
