# Supplementary material for: Cloning, analysis and functional annotation of expressed sequence tags from the Earthworm Eisenia fetida
Source: BMC Bioinformatics. 2007 Nov 1;8(Suppl 7):S7. doi: 10.1186/1471-2105-8-S7-S7 (PMC2099499; doi:10.1186/1471-2105-8-S7-S7)
Supplement: Additional file 1 — A complete listing of significant blastx hits (E ≤ 10-5) of the 2231 unique Eisenia fetida EST sequences matching four model organisms Mus musculus, Drosophila melanogaster, Caenorhabditis elegans, and Saccharomyces cerevisiae. [file 1471-2105-8-S7-S7-S1.doc]

| **Gene Ontology info for EarthWorm** |  |
| --- | --- |
|  |  |
| **Drosophila Melanogaster** | 265 |
| **Mus Musculus** | 447 |
| **Saccharomyces Cerevisiae** | 5 |
| **Caenorhabditis Elegans** | 113 |
| **All** | 830 |

| QueryID | Accession Version # | gene name | db_xref | QueryID | Accession Version # | gene name | db_xref |
| --- | --- | --- | --- | --- | --- | --- | --- |
| Contig1 | AAH55439.1 | Myl9 | [MGI:2138915](http://www.informatics.jax.org/searches/accession_report.cgi?id=MGI:2138915) | EW1_F2plate13_H08 | NP_722674.1 | s | [FBgn0003310](http://flybase.bio.indiana.edu/.bin/fbidq.html?FBgn0003310) |
| Contig1 | CAA86772.1 | mlc-4 | [WBGene00003372](http://www.wormbase.org/db/gene/gene?name=WBGene00003372;class=Gene) | EW1_F2plate14_A04 | AAV65815.1 | Aldrl6 | [MGI:1891725](http://www.informatics.jax.org/searches/accession_report.cgi?id=MGI:1891725) |
| Contig10 | AAR99150.1 | CG1746 | [FBgn0039830](http://flybase.bio.indiana.edu/.bin/fbidq.html?FBgn0039830) | EW1_F2plate14_A08 | NP_609321.1 | CG5885 | [FBgn0025700](http://flybase.bio.indiana.edu/.bin/fbidq.html?FBgn0025700) |
| Contig105 | NP_056623.1 | Krtap5-1 | [MGI:1354732](http://www.informatics.jax.org/searches/accession_report.cgi?id=MGI:1354732) | EW1_F2plate14_B05 | AAA80448.2 | F41C6.7 | [WBGene00018280](http://www.wormbase.org/db/gene/gene?class=CDS;name=WBGene00018280) |
| Contig105 | NP_056624.1 | Krtap5-4 | [MGI:1354758](http://www.informatics.jax.org/searches/accession_report.cgi?id=MGI:1354758) | EW1_F2plate14_B05 | NP_498611.1 | F56C9.3 | [WBGene00018948](http://www.wormbase.org/db/gene/gene?class=CDS;name=WBGene00018948) |
| Contig105 | NP_081083.1 | 1110033F04Rik | [MGI:1915923](http://www.informatics.jax.org/searches/accession_report.cgi?id=MGI:1915923) | EW1_F2plate14_B05 | AAV28339.1 | R02F11.3 | [WBGene00019841](http://www.wormbase.org/db/gene/gene?name=WBGene00019841;class=Gene) |
| Contig105 | NP_083889.1 | Krtap4-7 | [MGI:1923694](http://www.informatics.jax.org/searches/accession_report.cgi?id=MGI:1923694) | EW1_F2plate14_D06 | CAA68781.1 | Hex | [MGI:96073](http://www.informatics.jax.org/searches/accession_report.cgi?id=MGI:96073) |
| Contig105 | AAI11114.1 | Krtap5-5 | [MGI:2149673](http://www.informatics.jax.org/searches/accession_report.cgi?id=MGI:2149673) | EW1_F2plate14_E07 | NP_609402.1 | CG5343 | [FBgn0032248](http://flybase.bio.indiana.edu/.bin/fbidq.html?FBgn0032248) |
| Contig108 | AAH60707.1 | Eef2 | [MGI:95288](http://www.informatics.jax.org/searches/accession_report.cgi?id=MGI:95288) | EW1_F2plate14_E07 | NP_723611.1 | CG17118 | [FBgn0032291](http://flybase.bio.indiana.edu/.bin/fbidq.html?FBgn0032291) |
| Contig109 | NP_733277.1 | CG14516 | [FBgn0039640](http://flybase.bio.indiana.edu/.bin/fbidq.html?FBgn0039640) | EW1_F2plate14_E07 | NP_032213.1 | Gtl3 | [MGI:107428](http://www.informatics.jax.org/searches/accession_report.cgi?id=MGI:107428) |
| Contig110 | AAH22602.1 | 1110039B18Rik | [MGI:1916046](http://www.informatics.jax.org/searches/accession_report.cgi?id=MGI:1916046) | EW1_F2plate14_G02 | AAH56999.1 | Pnpla6 | [MGI:1354723](http://www.informatics.jax.org/searches/accession_report.cgi?id=MGI:1354723) |
| Contig116 | NP_608710.1 | aph-1 | [FBgn0031458](http://flybase.bio.indiana.edu/.bin/fbidq.html?FBgn0031458) | EW1_F2plate14_G02 | AAH25621.1 | Pnpla7 | [MGI:2385325](http://www.informatics.jax.org/searches/accession_report.cgi?id=MGI:2385325) |
| Contig116 | AAH24111.1 | Aph1a | [MGI:2385110](http://www.informatics.jax.org/searches/accession_report.cgi?id=MGI:2385110) | EW1_F2plate14_H02 | AAH03780.1 | Chi3l1 | [MGI:1340899](http://www.informatics.jax.org/searches/accession_report.cgi?id=MGI:1340899) |
| Contig119 | NP_084289.1 | Cyp20a1 | [MGI:1925201](http://www.informatics.jax.org/searches/accession_report.cgi?id=MGI:1925201) | EW1_F2plate14_H02 | CAA63603.1 | brp39 | [MGI:1340899](http://www.informatics.jax.org/searches/accession_report.cgi?id=MGI:1340899) |
| Contig125 | NP_648588.1 | CG10627 | [FBgn0036298](http://flybase.bio.indiana.edu/.bin/fbidq.html?FBgn0036298) | EW1_F2plate14_H04 | AAV36864.1 | CG7671 | [FBgn0038609](http://flybase.bio.indiana.edu/.bin/fbidq.html?FBgn0038609) |
| Contig127 | AAI06104.1 | 1190005P17Rik | [MGI:1913475](http://www.informatics.jax.org/searches/accession_report.cgi?id=MGI:1913475) | EW1_F2plate14_H04 | AAH52530.2 | Nup43 | [MGI:1917162](http://www.informatics.jax.org/searches/accession_report.cgi?id=MGI:1917162) |
| Contig13 | AAH19190.1 | Fap | [MGI:109608](http://www.informatics.jax.org/searches/accession_report.cgi?id=MGI:109608) | EW1_F2plate15_B01 | AAH24896.1 | Copg | [MGI:1858696](http://www.informatics.jax.org/searches/accession_report.cgi?id=MGI:1858696) |
| Contig132 | NP_523400.1 | betaCop | [FBgn0008635"](http://flybase.bio.indiana.edu/.bin/fbidq.html?FBgn0008635) | EW1_F2plate15_D10 | AAH60991.1 | Cetn4 | [MGI:2677454](http://www.informatics.jax.org/searches/accession_report.cgi?id=MGI:2677454) |
| Contig132 | AAH30837.1 | Copb1 | [MGI:1917599"](http://www.informatics.jax.org/searches/accession_report.cgi?id=MGI:1917599) | EW1_F2plate15_E02 | AAK30001.1 | Rdh1 | [MGI:1195275](http://www.informatics.jax.org/searches/accession_report.cgi?id=MGI:1195275) |
| Contig136 | NP_608668.2 | CG16995 | [FBgn0031412](http://flybase.bio.indiana.edu/.bin/fbidq.html?FBgn0031412) | EW1_F2plate15_E02 | AAC46535.1 | F55E10.6 | [WBGene00018885](http://www.wormbase.org/db/gene/gene?class=CDS;name=WBGene00018885) |
| Contig136 | NP_731097.1 | CG31482 | [FBgn0051482](http://flybase.bio.indiana.edu/.bin/fbidq.html?FBgn0051482) | EW1_F2plate15_E09 | NP_033066.1 | Rdh16 | [MGI:1201375](http://www.informatics.jax.org/searches/accession_report.cgi?id=MGI:1201375) |
| Contig141 | AAR96131.1 | RpL3 | [FBgn0020910](http://flybase.bio.indiana.edu/.bin/fbidq.html?FBgn0020910) | EW1_F2plate15_E09 | NP_081577.1 | Sdro | [MGI:1917311](http://www.informatics.jax.org/searches/accession_report.cgi?id=MGI:1917311) |
| Contig143 | AAL39841.1 | Lmpt | [FBgn0036672](http://flybase.bio.indiana.edu/.bin/fbidq.html?FBgn0036672) | EW1_F2plate15_E11 | AAH86915.1 | Eif3s3 | [MGI:1915385](http://www.informatics.jax.org/searches/accession_report.cgi?id=MGI:1915385) |
| Contig143 | CAJ30230.1 | tag-15 | [WBGene00006407](http://www.wormbase.org/db/gene/gene?name=WBGene00006407;class=Gene) | EW1_F2plate15_F08 | CAD44093.1 | ifa-1 | [WBGene00002050](http://www.wormbase.org/db/searches/basic?class=Any&query=WBGene00002050) |
| Contig144 | AAH61131.1 | Csrp3 | [MGI:1330824](http://www.informatics.jax.org/searches/accession_report.cgi?id=MGI:1330824) | EW1_F2plate15_F10 | NP_001014737.1 | up | [FBgn0004169](http://flybase.bio.indiana.edu/.bin/fbidq.html?FBgn0004169) |
| Contig158 | AAH61504.1 | Mpdz | [MGI:1343489](http://www.informatics.jax.org/searches/accession_report.cgi?id=MGI:1343489) | EW1_F2plate15_F10 | AAR24587.1 | TpnT | [FBgn0004169](http://flybase.bio.indiana.edu/.bin/fbidq.html?FBgn0004169) |
| Contig162 | AAH46956.1 | M6pr | [MGI:96904](http://www.informatics.jax.org/searches/accession_report.cgi?id=MGI:96904) | EW1_F2plate15_G07 | NP_001014737.1 | TpnT | [FBgn0004169](http://flybase.bio.indiana.edu/.bin/fbidq.html?FBgn0004169) |
| Contig163 | BAA08076.1 | met10 | [SGD:S000001926](http://db.yeastgenome.org/cgi-bin/SGD/locus.pl?sgdid=S000001926) | EW1_F2plate15_G07 | AAR24583.1 | up | [FBgn0004169](http://flybase.bio.indiana.edu/.bin/fbidq.html?FBgn0004169) |
| Contig164 | AAI06122.1 | Rpl6 | [MGI:108057](http://www.informatics.jax.org/searches/accession_report.cgi?id=MGI:108057) | EW1_F2plate15_H06 | NP_730317.1 | Tsp74F | [FBgn0036769](http://flybase.bio.indiana.edu/.bin/fbidq.html?FBgn0036769) |
| Contig168 | AAH05671.1 | Dctn5 | [MGI:1891689](http://www.informatics.jax.org/searches/accession_report.cgi?id=MGI:1891689) | EW1_F2plate16_A04 | NP_996385.1 | CG12065 | [FBgn0030052](http://flybase.bio.indiana.edu/.bin/fbidq.html?FBgn0030052) |
| Contig174 | NP_034022.1 | Chi3l3 | [MGI:1330860](http://www.informatics.jax.org/searches/accession_report.cgi?id=MGI:1330860) | EW1_F2plate16_A04 | AAN63406.1 | H14E04.2 | [WBGene00019199](http://www.wormbase.org/db/gene/gene?class=CDS;name=WBGene00019199) |
| Contig178 | AAH02125.1 | Ctss | [MGI:107341](http://www.informatics.jax.org/searches/accession_report.cgi?id=MGI:107341) | EW1_F2plate16_A07 | NP_650073.1 | CG6764 | [FBgn0037899](http://flybase.bio.indiana.edu/.bin/fbidq.html?FBgn0037899) |
| Contig178 | AAH46320.1 | Ctsk | [MGI:107823](http://www.informatics.jax.org/searches/accession_report.cgi?id=MGI:107823) | EW1_F2plate16_A07 | NP_941011.1 | BC003885 | [MGI:2681840](http://www.informatics.jax.org/searches/accession_report.cgi?id=MGI:2681840) |
| Contig18 | NP_031647.1 | Cbr2 | [MGI:107200](http://www.informatics.jax.org/searches/accession_report.cgi?id=MGI:107200) | EW1_F2plate16_A08 | NP_650073.1 | CG6764 | [FBgn0037899](http://flybase.bio.indiana.edu/.bin/fbidq.html?FBgn0037899) |
| Contig18 | AAH12247.1 | Dcxr | [MGI:1915130](http://www.informatics.jax.org/searches/accession_report.cgi?id=MGI:1915130) | EW1_F2plate16_A08 | NP_941011.1 | BC003885 | [MGI:2681840](http://www.informatics.jax.org/searches/accession_report.cgi?id=MGI:2681840) |
| Contig181 | NP_035782.3 | Ttn | [MGI:98864](http://www.informatics.jax.org/searches/accession_report.cgi?id=MGI:98864) | EW1_F2plate16_A09 | NP_650073.1 | CG6764 | [FBgn0037899](http://flybase.bio.indiana.edu/.bin/fbidq.html?FBgn0037899) |
| Contig184 | NP_649159.1 | CG7770 | [FBgn0036918](http://flybase.bio.indiana.edu/.bin/fbidq.html?FBgn0036918) | EW1_F2plate16_A09 | NP_941011.1 | BC003885 | [MGI:2681840](http://www.informatics.jax.org/searches/accession_report.cgi?id=MGI:2681840) |
| Contig184 | CAA95804.1 | pfd-6 | [WBGene00009004](http://www.wormbase.org/db/gene/gene?name=WBGene00009004;class=Gene) | EW1_F2plate16_A10 | NP_650073.1 | CG6764 | [FBgn0037899](http://flybase.bio.indiana.edu/.bin/fbidq.html?FBgn0037899) |
| Contig186 | NP_808355.1 | Crebl2 | [MGI:1889385](http://www.informatics.jax.org/searches/accession_report.cgi?id=MGI:1889385) | EW1_F2plate16_A10 | NP_941011.1 | BC003885 | [MGI:2681840](http://www.informatics.jax.org/searches/accession_report.cgi?id=MGI:2681840) |
| Contig195 | AAH50197.1 | Usp14 | [MGI:1928898](http://www.informatics.jax.org/searches/accession_report.cgi?id=MGI:1928898) | EW1_F2plate16_A11 | NP_650073.1 | CG6764 | [FBgn0037899](http://flybase.bio.indiana.edu/.bin/fbidq.html?FBgn0037899) |
| Contig199 | AAR30181.1 | veli | [FBgn0039269](http://flybase.bio.indiana.edu/.bin/fbidq.html?FBgn0039269) | EW1_F2plate16_A11 | NP_941011.1 | BC003885 | [MGI:2681840](http://www.informatics.jax.org/searches/accession_report.cgi?id=MGI:2681840) |
| Contig199 | AAH31780.1 | Lin7b | [MGI:1330858](http://www.informatics.jax.org/searches/accession_report.cgi?id=MGI:1330858) | EW1_F2plate16_A12 | NP_650073.1 | CG6764 | [FBgn0037899](http://flybase.bio.indiana.edu/.bin/fbidq.html?FBgn0037899) |
| Contig201 | NP_524211.1 | RpLP0 | [FBgn0000100](http://flybase.bio.indiana.edu/.bin/fbidq.html?FBgn0000100) | EW1_F2plate16_A12 | NP_941011.1 | BC003885 | [MGI:2681840](http://www.informatics.jax.org/searches/accession_report.cgi?id=MGI:2681840) |
| Contig201 | AAH11291.1 | Arbp | [MGI:88066](http://www.informatics.jax.org/searches/accession_report.cgi?id=MGI:88066) | EW1_F2plate16_B01 | AAK21486.1 | lbp-5 | [WBGene00002257](http://www.wormbase.org/db/searches/basic?class=Any&query=WBGene00002257) |
| Contig206 | AAH58360.1 | Mat2a | [MGI:2443731](http://www.informatics.jax.org/searches/accession_report.cgi?id=MGI:2443731) | EW1_F2plate16_B02 | AAH23297.1 | Cpsf3 | [MGI:1859328](http://www.informatics.jax.org/searches/accession_report.cgi?id=MGI:1859328) |
| Contig21 | NP_081577.1 | Sdro | [MGI:1917311](http://www.informatics.jax.org/searches/accession_report.cgi?id=MGI:1917311) | EW1_F2plate16_B03 | AAH58630.1 | Ube1x | [MGI:98890](http://www.informatics.jax.org/searches/accession_report.cgi?id=MGI:98890) |
| Contig21 | AAB69884.1 | dhs-16 | [WBGene00000979](http://www.wormbase.org/db/gene/gene?class=CDS;name=WBGene00000979) | EW1_F2plate16_C08 | XP_903324.1 | Dennd3 | [MGI:2146009](http://www.informatics.jax.org/searches/accession_report.cgi?id=MGI:2146009) |
| Contig213 | AAH26816.1 | Sel1l | [MGI:1329016](http://www.informatics.jax.org/searches/accession_report.cgi?id=MGI:1329016) | EW1_F2plate16_D05 | NP_649477.1 | CG9783 | [FBgn0037256](http://flybase.bio.indiana.edu/.bin/fbidq.html?FBgn0037256) |
| Contig214 | NP_032039.2 | Fgl2 | [MGI:103266](http://www.informatics.jax.org/searches/accession_report.cgi?id=MGI:103266) | EW1_F2plate16_D05 | AAR28089.1 | Gucy2g | [MGI:106025](http://www.informatics.jax.org/searches/accession_report.cgi?id=MGI:106025) |
| Contig214 | AAH19828.1 | Fgg | [MGI:95526](http://www.informatics.jax.org/searches/accession_report.cgi?id=MGI:95526) | EW1_F2plate16_D07 | AAM11389.1 | Alk | [FBgn0040505](http://flybase.bio.indiana.edu/.bin/fbidq.html?FBgn0040505) |
| Contig215 | AAH05457.1 | Calml3 | [MGI:1917655](http://www.informatics.jax.org/searches/accession_report.cgi?id=MGI:1917655) | EW1_F2plate16_E09 | AAM11389.1 | Alk | [FBgn0040505](http://flybase.bio.indiana.edu/.bin/fbidq.html?FBgn0040505) |
| Contig222 | NP_611420.1 | cer | [FBgn0034443](http://flybase.bio.indiana.edu/.bin/fbidq.html?FBgn0034443) | EW1_F2plate16_F12 | AAH35278.1 | Osbpl11 | [MGI:2146553](http://www.informatics.jax.org/searches/accession_report.cgi?id=MGI:2146553) |
| Contig222 | AAK00509.1 | Cts2 | [MGI:1860275](http://www.informatics.jax.org/searches/accession_report.cgi?id=MGI:1860275) | EW1_F2plate16_H08 | AAH35278.1 | Osbpl11 | [MGI:2146553](http://www.informatics.jax.org/searches/accession_report.cgi?id=MGI:2146553) |
| Contig222 | AAH68241.1 | Cts8 | [MGI:1860275](http://www.informatics.jax.org/searches/accession_report.cgi?id=MGI:1860275) | EW1_F2Plate17_B06 | AAH35278.1 | Osbpl11 | [MGI:2146553](http://www.informatics.jax.org/searches/accession_report.cgi?id=MGI:2146553) |
| Contig228 | CAC32040.1 | EDF-1 | [MGI:1891227](http://www.informatics.jax.org/searches/accession_report.cgi?id=MGI:1891227) | EW1_F2Plate17_D07 | NP_723596.1 | Myo31DF | [FBgn0011673](http://flybase.bio.indiana.edu/.bin/fbidq.html?FBgn0011673) |
| Contig231 | NP_730672.1 | CG32446 | [FBgn0052446](http://flybase.bio.indiana.edu/.bin/fbidq.html?FBgn0052446) | EW1_F2Plate17_D07 | CAI25853.1 | Myo1d | [MGI:107728](http://www.informatics.jax.org/searches/accession_report.cgi?id=MGI:107728) |
| Contig231 | CAI35353.1 | Atox1 | [MGI:1333855](http://www.informatics.jax.org/searches/accession_report.cgi?id=MGI:1333855) | EW1_F2Plate19_A04 | AAH67063.1 | Ctsc | [MGI:109553](http://www.informatics.jax.org/searches/accession_report.cgi?id=MGI:109553) |
| Contig231 | AAL00882.1 | cuc-1 | [WBGene00000835](http://www.wormbase.org/db/gene/gene?class=CDS;name=WBGene00000835) | EW1_F2Plate19_B05 | CAA67981.1 | ATPsyn-d | [FBgn0016120](http://flybase.bio.indiana.edu/.bin/fbidq.html?FBgn0016120) |
| Contig233 | AAV36956.1 | Cp1 | [FBgn0013770](http://flybase.bio.indiana.edu/.bin/fbidq.html?FBgn0013770) | EW1_F2Plate19_B06 | NP_724343.1 | His2A:CG31618 | [FBgn0051618](http://flybase.bio.indiana.edu/.bin/fbidq.html?FBgn0051618) |
| Contig236 | AAL89955.1 | CG18369 | [FBgn0033860](http://flybase.bio.indiana.edu/.bin/fbidq.html?FBgn0033860) | EW1_F2Plate19_B06 | NP_001027376.1 | His2A:CG33859 | [FBgn0053859](http://flybase.bio.indiana.edu/.bin/fbidq.html?FBgn0053859) |
| Contig236 | CAJ18445.1 | Lap3 | [MGI:1914238](http://www.informatics.jax.org/searches/accession_report.cgi?id=MGI:1914238) | EW1_F2Plate19_B06 | AAH10336.1 | H2afx | [MGI:102688](http://www.informatics.jax.org/searches/accession_report.cgi?id=MGI:102688) |
| Contig245 | NP_524480.2 | crb | [FBgn0000368](http://flybase.bio.indiana.edu/.bin/fbidq.html?FBgn0000368) | EW1_F2Plate19_C08 | AAH83327.1 | Rpl10 | [MGI:105943](http://www.informatics.jax.org/searches/accession_report.cgi?id=MGI:105943) |
| Contig245 | CAE48492.1 | Snep | [MGI:3045960](http://www.informatics.jax.org/searches/accession_report.cgi?id=MGI:3045960) | EW1_F2Plate19_D01 | NP_523366.2 | Cyp1 | [FBgn0004432](http://flybase.bio.indiana.edu/.bin/fbidq.html?FBgn0004432) |
| Contig246 | AAO41413.1 | EfTuM | [FBgn0024556](http://flybase.bio.indiana.edu/.bin/fbidq.html?FBgn0024556) | EW1_F2Plate19_D01 | NP_729966.1 | CG7768 | [FBgn0036415](http://flybase.bio.indiana.edu/.bin/fbidq.html?FBgn0036415) |
| Contig246 | AAI00597.1 | Tufm | [MGI:1923686](http://www.informatics.jax.org/searches/accession_report.cgi?id=MGI:1923686) | EW1_F2Plate19_E04 | XP_919947.1 | BC006779 | [MGI:2385169](http://www.informatics.jax.org/searches/accession_report.cgi?id=MGI:2385169) |
| Contig249 | AAL39841.1 | Lmpt | [FBgn0036672](http://flybase.bio.indiana.edu/.bin/fbidq.html?FBgn0036672) | EW1_F2Plate19_E05 | NP_524875.1 | arg | [FBgn0023535](http://flybase.bio.indiana.edu/.bin/fbidq.html?FBgn0023535) |
| Contig249 | CAJ30230.1 | tag-15 | [WBGene00006407](http://www.wormbase.org/db/gene/gene?name=WBGene00006407;class=Gene) | EW1_F2Plate19_G09 | XP_925719.1 | 3200001K10Rik | [MGI:1919670](http://www.informatics.jax.org/searches/accession_report.cgi?id=MGI:1919670) |
| Contig254 | AAF25478.1 | Chm | [MGI:892979](http://www.informatics.jax.org/searches/accession_report.cgi?id=MGI:892979) | EW1_F2Plate19_G10 | AAH03896.2 | Rpl17 | [MGI:2448270](http://www.informatics.jax.org/searches/accession_report.cgi?id=MGI:2448270) |
| Contig254 | AAP46267.1 | Y67D2.1 | [WBGene00022051](http://www.wormbase.org/db/gene/gene?class=CDS;name=WBGene00022051) | EW1_F2Plate19_H08 | CAA15707.1 | EG:30B8.3 | [FBgn0023525](http://flybase.bio.indiana.edu/.bin/fbidq.html?FBgn0023525) |
| Contig260 | AAK37595.1 | Ms4a4b | [MGI:1913083](http://www.informatics.jax.org/searches/accession_report.cgi?id=MGI:1913083) | EW1_F2Plate19_H08 | NP_726804.1 | CG3191 | [FBgn0023525](http://flybase.bio.indiana.edu/.bin/fbidq.html?FBgn0023525) |
| Contig261 | AAH18235.1 | Eef1a2 | [MGI:1096317](http://www.informatics.jax.org/searches/accession_report.cgi?id=MGI:1096317) | EW1_F2Plate19_H08 | AAL48733.1 | CG3091 | [FBgn0029608](http://flybase.bio.indiana.edu/.bin/fbidq.html?FBgn0029608) |
| Contig265 | AAH28865.1 | Bzw1 | [MGI:1914132](http://www.informatics.jax.org/searches/accession_report.cgi?id=MGI:1914132) | EW1_F2Plate19_H08 | NP_724192.1 | CG10237 | [FBgn0032783](http://flybase.bio.indiana.edu/.bin/fbidq.html?FBgn0032783) |
| Contig265 | AAH13060.1 | Bzw2 | [MGI:1914162](http://www.informatics.jax.org/searches/accession_report.cgi?id=MGI:1914162) | EW1_F2Plate19_H08 | NP_649535.1 | CG2663 | [FBgn0037323](http://flybase.bio.indiana.edu/.bin/fbidq.html?FBgn0037323) |
| Contig267 | AAR82815.1 | nAcRalpha-30D | [FBgn0032151](http://flybase.bio.indiana.edu/.bin/fbidq.html?FBgn0032151) | EW1_F2Plate20_A02 | AAD46929.2 | Ef1beta | [FBgn0028737](http://flybase.bio.indiana.edu/.bin/fbidq.html?FBgn0028737) |
| Contig269 | NP_082255.1 | Chit1 | [MGI:1919134](http://www.informatics.jax.org/searches/accession_report.cgi?id=MGI:1919134) | EW1_F2Plate20_A02 | CAA21314.1 | EG:EG0003.7 | [FBgn0028737](http://flybase.bio.indiana.edu/.bin/fbidq.html?FBgn0028737) |
| Contig27 | NP_611645.1 | Fmr1 | [FBgn0028734](http://flybase.bio.indiana.edu/.bin/fbidq.html?FBgn0028734) | EW1_F2Plate20_A12 | CAB53186.1 | Cbp80 | [FBgn0022942](http://flybase.bio.indiana.edu/.bin/fbidq.html?FBgn0022942) |
| Contig27 | CAD19443.1 | dfxr | [FBgn0028734](http://flybase.bio.indiana.edu/.bin/fbidq.html?FBgn0028734) | EW1_F2Plate20_A12 | AAH55777.1 | AU014645 | [MGI:2140419](http://www.informatics.jax.org/searches/accession_report.cgi?id=MGI:2140419) |
| Contig273 | AAR24583.1 | TpnT | [FBgn0004169](http://flybase.bio.indiana.edu/cgi-bin/fbidq.html?FBgn0004169) | EW1_F2Plate20_B03 | NP_694875.1 | Fbxo7 | [MGI:1917004](http://www.informatics.jax.org/searches/accession_report.cgi?id=MGI:1917004) |
| Contig273 | NP_525088.2 | up | [FBgn0004169](http://flybase.bio.indiana.edu/.bin/fbidq.html?FBgn0004169) | EW1_F2Plate20_C02 | NP_608996.1 | Arc-p20 | [FBgn0031781](http://flybase.bio.indiana.edu/.bin/fbidq.html?FBgn0031781) |
| Contig275 | NP_082255.1 | Chit1 | [MGI:1919134](http://www.informatics.jax.org/searches/accession_report.cgi?id=MGI:1919134) | EW1_F2Plate20_C02 | AAH55309.1 | Arpc4 | [MGI:1915339](http://www.informatics.jax.org/searches/accession_report.cgi?id=MGI:1915339) |
| Contig278 | AAH13480.1 | Cbs | [MGI:88285](http://www.informatics.jax.org/searches/accession_report.cgi?id=MGI:88285) | EW1_F2Plate20_D05 | AAS15671.1 | CG8680 | [FBgn0031684](http://flybase.bio.indiana.edu/.bin/fbidq.html?FBgn0031684) |
| Contig279 | AAH03471.1 | Npc2 | [MGI:1915213](http://www.informatics.jax.org/searches/accession_report.cgi?id=MGI:1915213) | EW1_F2Plate20_D05 | AAH86933.1 | Ndufs6 | [MGI:107932](http://www.informatics.jax.org/searches/accession_report.cgi?id=MGI:107932) |
| Contig28 | NP_082582.1 | Hspa12b | [MGI:1919880](http://www.informatics.jax.org/searches/accession_report.cgi?id=MGI:1919880) | EW1_F2Plate20_E03 | NP_648288.1 | Tequila | [FBgn0023479](http://flybase.bio.indiana.edu/.bin/fbidq.html?FBgn0023479) |
| Contig28 | AAH30362.1 | Hspa12a | [MGI:1920692](http://www.informatics.jax.org/searches/accession_report.cgi?id=MGI:1920692) | EW1_F2Plate20_E03 | AAK93496.1 | CG4821 | [FBgn0023479](http://flybase.bio.indiana.edu/.bin/fbidq.html?FBgn0023479) |
| Contig28 | BAC41423.1 | mKIAA0417 | [MGI:1920692](http://www.informatics.jax.org/searches/accession_report.cgi?id=MGI:1920692) | EW1_F2Plate20_E03 | CAC35209.1 | graal | [FBgn0023479](http://flybase.bio.indiana.edu/.bin/fbidq.html?FBgn0023479) |
| Contig284 | NP_651737.1 | CG18041 | [FBgn0039710](http://flybase.bio.indiana.edu/.bin/fbidq.html?FBgn0039710) | EW1_F2Plate20_E03 | NP_728468.1 | CG32499 | [FBgn0052499](http://flybase.bio.indiana.edu/.bin/fbidq.html?FBgn0052499) |
| Contig284 | AAH92275.1 | 2310037I24Rik | [MGI:1916862](http://www.informatics.jax.org/searches/accession_report.cgi?id=MGI:1916862) | EW1_F2Plate20_E03 | NP_788912.1 | CG33173 | [FBgn0053173](http://flybase.bio.indiana.edu/.bin/fbidq.html?FBgn0053173) |
| Contig286 | AAI06137.1 | Rps2 | [MGI:105110](http://www.informatics.jax.org/searches/accession_report.cgi?id=MGI:105110) | EW1_F2Plate20_E07 | AAH83340.1 | Naca | [MGI:106095](http://www.informatics.jax.org/searches/accession_report.cgi?id=MGI:106095) |
| Contig29 | NP_572407.2 | CG1402 | [FBgn0029962](http://flybase.bio.indiana.edu/.bin/fbidq.html?FBgn0029962) | EW1_F2Plate20_G03 | NP_477209.2 | T3dh | [FBgn0017482](http://flybase.bio.indiana.edu/.bin/fbidq.html?FBgn0017482) |
| Contig29 | AAH66111.1 | Car10 | [MGI:1919855](http://www.informatics.jax.org/searches/accession_report.cgi?id=MGI:1919855) | EW1_F2Plate20_G03 | AAH26584.2 | Adhfe1 | [MGI:1923437](http://www.informatics.jax.org/searches/accession_report.cgi?id=MGI:1923437) |
| Contig29 | NP_498083.1 | cah-1 | [WBGene00000279](http://www.wormbase.org/db/gene/gene?class=CDS;name=WBGene00000279) | EW1_F2Plate20_H03 | AAH13545.1 | Cetn2 | [MGI:1347085](http://www.informatics.jax.org/searches/accession_report.cgi?id=MGI:1347085) |
| Contig290 | NP_033006.1 | Ptpra | [MGI:97808](http://www.informatics.jax.org/searches/accession_report.cgi?id=MGI:97808) | EW1_F2Plate20_H09 | NP_524156.1 | rept | [FBgn0040075](http://flybase.bio.indiana.edu/.bin/fbidq.html?FBgn0040075) |
| Contig291 | NP_728141.1 | wupA | [FBgn0004028](http://flybase.bio.indiana.edu/.bin/fbidq.html?FBgn0004028) | EW1_F2Plate20_H11 | AAH27437.1 | Eif1a | [MGI:95298](http://www.informatics.jax.org/searches/accession_report.cgi?id=MGI:95298) |
| Contig292 | AAB68073.1 | PRE2 | [SGD:S000006307](http://db.yeastgenome.org/cgi-bin/SGD/locus.pl?sgdid=S000006307) | EW1_R1plate01_B09 | CAA52754.1 | STK | [MGI:99614](http://www.informatics.jax.org/searches/accession_report.cgi?id=MGI:99614) |
| Contig293 | NP_509939.1 | ftt-2 | [WBGene00001502](http://www.wormbase.org/db/gene/gene?class=CDS;name=WBGene00001502) | EW1_R1plate01_C01 | NP_649765.1 | CG7910 | [FBgn0037547](http://flybase.bio.indiana.edu/.bin/fbidq.html?FBgn0037547) |
| Contig295 | NP_523366.2 | Cyp1 | [FBgn0004432](http://flybase.bio.indiana.edu/.bin/fbidq.html?FBgn0004432) | EW1_R1plate01_C01 | NP_649766.2 | CG7900 | [FBgn0037548](http://flybase.bio.indiana.edu/.bin/fbidq.html?FBgn0037548) |
| Contig295 | NP_729966.1 | CG7768 | [FBgn0036415](http://flybase.bio.indiana.edu/.bin/fbidq.html?FBgn0036415) | EW1_R1plate01_C01 | NP_651400.1 | CG5112 | [FBgn0039341](http://flybase.bio.indiana.edu/.bin/fbidq.html?FBgn0039341) |
| Contig299 | NP_033102.1 | Rpl12 | [MGI:98002](http://www.informatics.jax.org/searches/accession_report.cgi?id=MGI:98002) | EW1_R1plate01_C09 | BAA01894.1 | DGK | [FBgn0004568](http://flybase.bio.indiana.edu/.bin/fbidq.html?FBgn0004568) |
| Contig30 | NP_035808.1 | Usp4 | [MGI:98905](http://www.informatics.jax.org/searches/accession_report.cgi?id=MGI:98905) | EW1_R1plate01_E03 | AAR99133.1 | CG6686 | [FBgn0032388](http://flybase.bio.indiana.edu/.bin/fbidq.html?FBgn0032388) |
| Contig301 | AAL68137.1 | CG15524 | [FBgn0039731](http://flybase.bio.indiana.edu/.bin/fbidq.html?FBgn0039731) | EW1_R1plate01_E03 | AAH51394.1 | Sart1 | [MGI:1309453](http://www.informatics.jax.org/searches/accession_report.cgi?id=MGI:1309453) |
| Contig305 | AAS18679.1 | Ap3b2 | [MGI:1100869](http://www.informatics.jax.org/searches/accession_report.cgi?id=MGI:1100869) | EW1_R1plate01_H07 | NP_033569.1 | Rnf103 | [MGI:109483](http://www.informatics.jax.org/searches/accession_report.cgi?id=MGI:109483) |
| Contig305 | NP_033810.1 | Ap3b1 | [MGI:1333879](http://www.informatics.jax.org/searches/accession_report.cgi?id=MGI:1333879) | EW1_R1plate02_A03 | NP_500208.1 | C45G7.1 | [WBGene00016668](http://www.wormbase.org/db/gene/gene?class=CDS;name=WBGene00016668) |
| Contig311 | NP_056624.1 | Krtap5-4 | [MGI:1354758](http://www.informatics.jax.org/searches/accession_report.cgi?id=MGI:1354758) | EW1_R1plate02_A03 | NP_500207.1 | C45G7.2 | [WBGene00016669](http://www.wormbase.org/db/gene/gene?class=CDS;name=WBGene00016669) |
| Contig312 | AAI06153.1 | Rps4x | [MGI:98158](http://www.informatics.jax.org/searches/accession_report.cgi?id=MGI:98158) | EW1_R1plate02_A03 | NP_500206.1 | C45G7.3 | [WBGene00016670](http://www.wormbase.org/db/gene/gene?class=CDS;name=WBGene00016670) |
| Contig318 | AAH86899.1 | Rps8 | [MGI:98166](http://www.informatics.jax.org/searches/accession_report.cgi?id=MGI:98166) | EW1_R1plate02_A03 | NP_501313.2 | C55F2.2 | [WBGene00016958](http://www.wormbase.org/db/gene/gene?class=CDS;name=WBGene00016958) |
| Contig321 | AAH13498.1 | Acadm | [MGI:87867](http://www.informatics.jax.org/searches/accession_report.cgi?id=MGI:87867) | EW1_R1plate02_A03 | AAA83197.1 | F22A3.6 | [WBGene00017691](http://www.wormbase.org/db/searches/basic?class=Any&query=WBGene00017691) |
| Contig331 | NP_572331.2 | CG3973 | [FBgn0029881](http://flybase.bio.indiana.edu/.bin/fbidq.html?FBgn0029881) | EW1_R1plate02_A06 | NP_524230.2 | UbcD6 | [FBgn0004436](http://flybase.bio.indiana.edu/.bin/fbidq.html?FBgn0004436) |
| Contig331 | AAH31785.1 | Gas2l1 | [MGI:1926176](http://www.informatics.jax.org/searches/accession_report.cgi?id=MGI:1926176) | EW1_R1plate02_A06 | AAC02561.2 | ubc-1 | [WBGene00006701](http://www.wormbase.org/db/searches/basic?class=Any&query=WBGene00006701) |
| Contig347 | AAH88733.1 | Usp28 | [MGI:2442293](http://www.informatics.jax.org/searches/accession_report.cgi?id=MGI:2442293) | EW1_R1plate02_C11 | CAB46016.1 | KIF9 | [MGI:1098237](http://www.informatics.jax.org/searches/accession_report.cgi?id=MGI:1098237) |
| Contig35 | AAO39656.1 | CG13349 | [FBgn0033886](http://flybase.bio.indiana.edu/.bin/fbidq.html?FBgn0033886) | EW1_R1plate02_D07 | AAH66197.1 | Ubb | [MGI:98888](http://www.informatics.jax.org/searches/accession_report.cgi?id=MGI:98888) |
| Contig35 | AAH31517.1 | Adrm1 | [MGI:1929289](http://www.informatics.jax.org/searches/accession_report.cgi?id=MGI:1929289) | EW1_R1plate02_E09 | AAH49638.1 | Rnut1 | [MGI:1913319](http://www.informatics.jax.org/searches/accession_report.cgi?id=MGI:1913319) |
| Contig353 | AAK21364.1 | ftn-2 | [WBGene00001501](http://www.wormbase.org/db/gene/gene?name=WBGene00001501;class=Gene) | EW1_R1plate02_E09 | AAB70323.1 | F23F1.5 | [WBGene00017746](http://www.wormbase.org/db/searches/basic?class=Any&query=WBGene00017746) |
| Contig356 | NP_033806.1 | Aox1 | [MGI:88035](http://www.informatics.jax.org/searches/accession_report.cgi?id=MGI:88035) | EW1_R1plate02_F07 | NP_647717.1 | CG9004 | [FBgn0035336](http://flybase.bio.indiana.edu/.bin/fbidq.html?FBgn0035336) |
| Contig360 | NP_608897.1 | CG3887 | [FBgn0031670](http://flybase.bio.indiana.edu/.bin/fbidq.html?FBgn0031670) | EW1_R1plate02_G05 | AAH36558.1 | Matn4 | [MGI:1328314](http://www.informatics.jax.org/searches/accession_report.cgi?id=MGI:1328314) |
| Contig363 | NP_610710.1 | ERp60 | [FBgn0033663](http://flybase.bio.indiana.edu/.bin/fbidq.html?FBgn0033663) | EW1_R1plate02_G06 | AAH75704.1 | Aldh1a2 | [MGI:107928](http://www.informatics.jax.org/searches/accession_report.cgi?id=MGI:107928) |
| Contig363 | AAH33439.1 | Pdia3 | [MGI:95834](http://www.informatics.jax.org/searches/accession_report.cgi?id=MGI:95834) | EW1_R1plate02_G06 | AAH58277.1 | Aldh1a3 | [MGI:1861722](http://www.informatics.jax.org/searches/accession_report.cgi?id=MGI:1861722) |
| Contig373 | NP_034022.1 | Chi3l3 | [MGI:1330860](http://www.informatics.jax.org/searches/accession_report.cgi?id=MGI:1330860) | EW1_R1plate02_G08 | NP_648712.1 | CG5048 | [FBgn0036437](http://flybase.bio.indiana.edu/.bin/fbidq.html?FBgn0036437) |
| Contig373 | AAL57751.1 | Chi3l4 | [MGI:1341098](http://www.informatics.jax.org/searches/accession_report.cgi?id=MGI:1341098) | EW1_R1plate02_G08 | AAI07263.1 | 4930521A18Rik | [MGI:1921958](http://www.informatics.jax.org/searches/accession_report.cgi?id=MGI:1921958) |
| Contig373 | AAL03953.2 | Ym2 | [MGI:1341098](http://www.informatics.jax.org/searches/accession_report.cgi?id=MGI:1341098) | EW1_R1plate02_G09 | NP_477369.1 | TER94 | [FBgn0024923](http://flybase.bio.indiana.edu/.bin/fbidq.html?FBgn0024923) |
| Contig374 | AAH02233.1 | Eef2 | [MGI:95288](http://www.informatics.jax.org/searches/accession_report.cgi?id=MGI:95288) | EW1_R1plate03_B09 | AAH10403.1 | Psen2 | [MGI:109284](http://www.informatics.jax.org/searches/accession_report.cgi?id=MGI:109284) |
| Contig379 | NP_081571.1 | Rab28 | [MGI:1917285](http://www.informatics.jax.org/searches/accession_report.cgi?id=MGI:1917285) | EW1_R1plate03_C01 | AAH04039.1 | Fuca2 | [MGI:1914098](http://www.informatics.jax.org/searches/accession_report.cgi?id=MGI:1914098) |
| Contig379 | NP_501609.1 | rab-28 | [WBGene00004281](http://www.wormbase.org/db/gene/gene?class=CDS;name=WBGene00004281) | EW1_R1plate03_D04 | AAA76733.1 | Acadm | [MGI:87867](http://www.informatics.jax.org/searches/accession_report.cgi?id=MGI:87867) |
| Contig381 | NP_081561.2 | 1700029I01Rik | [MGI:1917255](http://www.informatics.jax.org/searches/accession_report.cgi?id=MGI:1917255) | EW1_R1plate03_D08 | AAH82558.1 | Tbccd1 | [MGI:1917823](http://www.informatics.jax.org/searches/accession_report.cgi?id=MGI:1917823) |
| Contig383 | AAI00349.1 | Crim1 | [MGI:1354756](http://www.informatics.jax.org/searches/accession_report.cgi?id=MGI:1354756) | EW1_R1plate03_E12 | AAH22991.1 | Fmo5 | [MGI:1310004](http://www.informatics.jax.org/searches/accession_report.cgi?id=MGI:1310004) |
| Contig383 | AAB88311.2 | C08G9.2 | [WBGene00015620](http://www.wormbase.org/db/gene/gene?class=CDS;name=WBGene00015620) | EW1_R1plate03_F07 | CAB46016.1 | KIF9 | [MGI:1098237](http://www.informatics.jax.org/searches/accession_report.cgi?id=MGI:1098237) |
| Contig385 | NP_476630.1 | RpLP1 | [FBgn0002593](http://flybase.bio.indiana.edu/.bin/fbidq.html?FBgn0002593) | EW1_R1plate03_F10 | AAH04039.1 | Fuca2 | [MGI:1914098](http://www.informatics.jax.org/searches/accession_report.cgi?id=MGI:1914098) |
| Contig48 | AAH58966.1 | Pcmt1 | [MGI:97502](http://www.informatics.jax.org/searches/accession_report.cgi?id=MGI:97502) | EW1_R1plate03_G02 | NP_524311.1 | Sodh-2 | [FBgn0022359](http://flybase.bio.indiana.edu/.bin/fbidq.html?FBgn0022359) |
| Contig48 | AAB69887.1 | pcm-1 | [WBGene00003954](http://www.wormbase.org/db/gene/gene?class=CDS;name=WBGene00003954) | EW1_R1plate03_G02 | NP_477348.1 | Sodh-1 | [FBgn0024289](http://flybase.bio.indiana.edu/.bin/fbidq.html?FBgn0024289) |
| Contig5 | AAH24113.1 | Cul4a | [MGI:1914487](http://www.informatics.jax.org/searches/accession_report.cgi?id=MGI:1914487) | EW1_R1plate03_G07 | NP_650837.1 | CG4686 | [FBgn0038739](http://flybase.bio.indiana.edu/.bin/fbidq.html?FBgn0038739) |
| Contig52 | NP_036178.1 | Polk | [MGI:1349767](http://www.informatics.jax.org/searches/accession_report.cgi?id=MGI:1349767) | EW1_R1plate04_B10 | NP_938041.1 | E130303B06Rik | [MGI:2142593](http://www.informatics.jax.org/searches/accession_report.cgi?id=MGI:2142593) |
| Contig55 | AAV36944.1 | Cad99C | [FBgn0039709](http://flybase.bio.indiana.edu/.bin/fbidq.html?FBgn0039709) | EW1_R1plate04_D09 | NP_071705.2 | Hspa5 | [MGI:95835](http://www.informatics.jax.org/searches/accession_report.cgi?id=MGI:95835) |
| Contig55 | NP_075604.1 | Pcdh15 | [MGI:1891428](http://www.informatics.jax.org/searches/accession_report.cgi?id=MGI:1891428) | EW1_R1plate04_H07 | NP_726251.1 | CG30266 | [FBgn0050266](http://flybase.bio.indiana.edu/.bin/fbidq.html?FBgn0050266) |
| Contig58 | AAR96134.1 | CG12079 | [FBgn0035404](http://flybase.bio.indiana.edu/.bin/fbidq.html?FBgn0035404) | EW1_R1plate04_H07 | AAH47067.1 | Ankrd26 | [MGI:1917887](http://www.informatics.jax.org/searches/accession_report.cgi?id=MGI:1917887) |
| Contig58 | AAH27270.1 | Ndufs3 | [MGI:1915599](http://www.informatics.jax.org/searches/accession_report.cgi?id=MGI:1915599) | EW1_R1plate05_B05 | NP_649765.1 | CG7910 | [FBgn0037547](http://flybase.bio.indiana.edu/.bin/fbidq.html?FBgn0037547) |
| Contig64 | NP_648754.2 | FucTA | [FBgn0036485](http://flybase.bio.indiana.edu/.bin/fbidq.html?FBgn0036485) | EW1_R1plate05_B05 | NP_649766.2 | CG7900 | [FBgn0037548](http://flybase.bio.indiana.edu/.bin/fbidq.html?FBgn0037548) |
| Contig65 | NP_609642.1 | CG9306 | [FBgn0032511](http://flybase.bio.indiana.edu/.bin/fbidq.html?FBgn0032511) | EW1_R1plate05_B05 | NP_651400.1 | CG5112 | [FBgn0039341](http://flybase.bio.indiana.edu/.bin/fbidq.html?FBgn0039341) |
| Contig65 | AAH31539.1 | Ndufb9 | [MGI:1913468](http://www.informatics.jax.org/searches/accession_report.cgi?id=MGI:1913468) | EW1_R1plate05_C01 | NP_796062.1 | Ccdc45 | [MGI:2443502](http://www.informatics.jax.org/searches/accession_report.cgi?id=MGI:2443502) |
| Contig66 | NP_032856.1 | Abcb4 | [MGI:97569](http://www.informatics.jax.org/searches/accession_report.cgi?id=MGI:97569) | EW1_R1plate05_C11 | AAS77442.1 | CG11796 | [FBgn0036992](http://flybase.bio.indiana.edu/.bin/fbidq.html?FBgn0036992) |
| Contig69 | AAH22991.1 | Fmo5 | [MGI:1310004](http://www.informatics.jax.org/searches/accession_report.cgi?id=MGI:1310004) | EW1_R1plate05_C11 | AAH13343.1 | Hpd | [MGI:96213](http://www.informatics.jax.org/searches/accession_report.cgi?id=MGI:96213) |
| Contig7 | AAM22077.1 | H13 | [MGI:95886](http://www.informatics.jax.org/searches/accession_report.cgi?id=MGI:95886) | EW1_R1plate05_E07 | NP_733305.1 | ATPsyn-gamma | [FBgn0020235](http://flybase.bio.indiana.edu/.bin/fbidq.html?FBgn0020235) |
| Contig75 | NP_081577.1 | Sdro | [MGI:1917311](http://www.informatics.jax.org/searches/accession_report.cgi?id=MGI:1917311) | EW1_R1plate05_E07 | AAK68562.1 | Y69A2AR.18 | [WBGene00022089](http://www.wormbase.org/db/gene/gene?class=CDS;name=WBGene00022089) |
| Contig78 | AAH31746.1 | Rps9 | [MGI:1924096](http://www.informatics.jax.org/searches/accession_report.cgi?id=MGI:1924096) | EW1_R1plate05_F01 | NP_652634.1 | CG3706 | [FBgn0040342](http://flybase.bio.indiana.edu/.bin/fbidq.html?FBgn0040342) |
| Contig8 | NP_647717.1 | CG9004 | [FBgn0035336](http://flybase.bio.indiana.edu/.bin/fbidq.html?FBgn0035336) | EW1_R1plate05_F01 | AAC02725.3 | C05D2.8 | [WBGene00015469](http://www.wormbase.org/db/gene/gene?class=CDS;name=WBGene00015469) |
| Contig86 | NP_724964.1 | CG7220 | [FBgn0033544](http://flybase.bio.indiana.edu/.bin/fbidq.html?FBgn0033544) | EW1_R1plate05_G05 | NP_725346.1 | CG13349 | [FBgn0033886](http://flybase.bio.indiana.edu/.bin/fbidq.html?FBgn0033886) |
| Contig86 | AAH20124.1 | Ube2w | [MGI:1914049](http://www.informatics.jax.org/searches/accession_report.cgi?id=MGI:1914049) | EW1_R1plate05_G05 | AAH19746.1 | Adrm1 | [MGI:1929289](http://www.informatics.jax.org/searches/accession_report.cgi?id=MGI:1929289) |
| Contig87 | AAH55732.1 | Keap1 | [MGI:1858732](http://www.informatics.jax.org/searches/accession_report.cgi?id=MGI:1858732) | EW1_R1plate05_H11 | NP_524311.1 | Sodh-2 | [FBgn0022359](http://flybase.bio.indiana.edu/.bin/fbidq.html?FBgn0022359) |
| Contig91 | CAD60206.1 | Aam-B | [MGI:1916523](http://www.informatics.jax.org/searches/accession_report.cgi?id=MGI:1916523) | EW1_R1plate05_H11 | NP_477348.1 | Sodh-1 | [FBgn0024289](http://flybase.bio.indiana.edu/.bin/fbidq.html?FBgn0024289) |
| Contig91 | AAH55034.1 | Mettl7a | [MGI:1916523](http://www.informatics.jax.org/searches/accession_report.cgi?id=MGI:1916523) | EW1_R1plate06_B02 | NP_082255.1 | Chit1 | [MGI:1919134](http://www.informatics.jax.org/searches/accession_report.cgi?id=MGI:1919134) |
| Contig91 | NP_082129.2 | Mettl7b | [MGI:1918914](http://www.informatics.jax.org/searches/accession_report.cgi?id=MGI:1918914) | EW1_R1plate06_B10 | AAH24850.1 | Utp6 | [MGI:2445193](http://www.informatics.jax.org/searches/accession_report.cgi?id=MGI:2445193) |
| Contig91 | NP_955771.2 | Ubie | [MGI:3026615](http://www.informatics.jax.org/searches/accession_report.cgi?id=MGI:3026615) | EW1_R1plate06_D08 | NP_651284.1 | CG5805 | [FBgn0039223](http://flybase.bio.indiana.edu/.bin/fbidq.html?FBgn0039223) |
| Contig92 | NP_056624.1 | Krtap5-4 | [MGI:1354758](http://www.informatics.jax.org/searches/accession_report.cgi?id=MGI:1354758) | EW1_R1plate06_D08 | AAH52771.2 | Slc25a44 | [MGI:2444391](http://www.informatics.jax.org/searches/accession_report.cgi?id=MGI:2444391) |
| Contig93 | XP_906141.1 | Pmpcb | [MGI:1920328](http://www.informatics.jax.org/searches/accession_report.cgi?id=MGI:1920328) | EW1_R1plate06_D10 | AAH28538.1 | Mrpl55 | [MGI:1914462](http://www.informatics.jax.org/searches/accession_report.cgi?id=MGI:1914462) |
| Contig94 | AAH30344.1 | Txnl5 | [MGI:1289248](http://www.informatics.jax.org/searches/accession_report.cgi?id=MGI:1289248) | EW1_R1plate06_D10 | AAD12855.2 | tag-313 | [WBGene00022045](http://www.wormbase.org/db/gene/gene?class=CDS;name=WBGene00022045) |
| Contig97 | NP_524714.1 | hoip | [FBgn0015393](http://flybase.bio.indiana.edu/.bin/fbidq.html?FBgn0015393) | EW1_R1plate06_D11 | NP_523388.1 | beta-Spec | [FBgn0003471](http://flybase.bio.indiana.edu/.bin/fbidq.html?FBgn0003471) |
| Contig98 | NP_034831.1 | Lect1 | [MGI:1341171](http://www.informatics.jax.org/searches/accession_report.cgi?id=MGI:1341171) | EW1_R1plate06_D11 | AAK77612.2 | unc-70 | [WBGene00006803](http://www.wormbase.org/db/gene/gene?class=CDS;name=WBGene00006803) |
| Contig99 | NP_872117.1 | T22F3.3b | [WBGene00020696](http://www.wormbase.org/db/gene/gene?class=CDS;name=WBGene00020696) | EW1_R1plate06_E08 | NP_523524.2 | Gdi | [FBgn0004868](http://flybase.bio.indiana.edu/.bin/fbidq.html?FBgn0004868) |
| EW1_F1plate01_B01 | AAL68189.2 | CG5703 | [FBgn0030853](http://flybase.bio.indiana.edu/.bin/fbidq.html?FBgn0030853) | EW1_R1plate06_E08 | AAH13758.1 | Gdi1 | [MGI:99846](http://www.informatics.jax.org/searches/accession_report.cgi?id=MGI:99846) |
| EW1_F1plate01_B01 | AAH30946.1 | Ndufv2 | [MGI:1920150](http://www.informatics.jax.org/searches/accession_report.cgi?id=MGI:1920150) | EW1_R1plate06_F01 | AAH19987.1 | Morn3 | [MGI:1922140](http://www.informatics.jax.org/searches/accession_report.cgi?id=MGI:1922140) |
| EW1_F1plate01_B02 | AAK73930.1 | Y71G12B.4 | [WBGene00022144](http://www.wormbase.org/db/gene/gene?class=CDS;name=WBGene00022144) | EW1_R1plate06_F03 | NP_731253.1 | CG31460 | [FBgn0051460](http://flybase.bio.indiana.edu/.bin/fbidq.html?FBgn0051460) |
| EW1_F1plate01_B03 | AAH21768.1 | H3f3b | [MGI:1101768](http://www.informatics.jax.org/searches/accession_report.cgi?id=MGI:1101768) | EW1_R1plate06_F03 | AAH43030.1 | Krtcap2 | [MGI:1913309](http://www.informatics.jax.org/searches/accession_report.cgi?id=MGI:1913309) |
| EW1_F1plate01_B04 | AAH17514.1 | Rabl4 | [MGI:1914292](http://www.informatics.jax.org/searches/accession_report.cgi?id=MGI:1914292) | EW1_R1plate06_G11 | NP_612087.1 | Sac1 | [FBgn0035195](http://flybase.bio.indiana.edu/.bin/fbidq.html?FBgn0035195) |
| EW1_F1plate01_B08 | NP_663509.2 | Btbd3 | [MGI:2385155](http://www.informatics.jax.org/searches/accession_report.cgi?id=MGI:2385155) | EW1_R1plate06_G11 | NP_492518.1 | F30A10.6 | [WBGene00009264](http://www.wormbase.org/db/gene/gene?class=CDS;name=WBGene00009264) |
| EW1_F1plate01_B08 | NP_964008.1 | Btbd6 | [MGI:3026623](http://www.informatics.jax.org/searches/accession_report.cgi?id=MGI:3026623) | EW1_R1plate07_B01 | AAH03283.1 | Pabpc4 | [MGI:2385206](http://www.informatics.jax.org/searches/accession_report.cgi?id=MGI:2385206) |
| EW1_F1plate01_B10 | NP_477445.1 | ARP-like | [FBgn0027095](http://flybase.bio.indiana.edu/.bin/fbidq.html?FBgn0027095) | EW1_R1plate07_B03 | NP_062770.1 | Syncrip | [MGI:1891690](http://www.informatics.jax.org/searches/accession_report.cgi?id=MGI:1891690) |
| EW1_F1plate01_B10 | AAK93864.2 | Y54G2A.23 | [WBGene00021888](http://www.wormbase.org/db/gene/gene?class=CDS;name=WBGene00021888) | EW1_R1plate07_B03 | AAH38051.1 | Hnrpr | [MGI:1891692](http://www.informatics.jax.org/searches/accession_report.cgi?id=MGI:1891692) |
| EW1_F1plate01_B11 | AAH03896.2 | Rpl17 | [MGI:2448270](http://www.informatics.jax.org/searches/accession_report.cgi?id=MGI:2448270) | EW1_R1plate07_C09 | CAD44093.1 | ifa-1 | [WBGene00002050](http://www.wormbase.org/db/searches/basic?class=Any&query=WBGene00002050) |
| EW1_F1plate01_C06 | AAH28865.1 | Bzw1 | [MGI:1914132](http://www.informatics.jax.org/searches/accession_report.cgi?id=MGI:1914132) | EW1_R1plate07_E01 | AAH85618.1 | Hspg2 | [MGI:96257](http://www.informatics.jax.org/searches/accession_report.cgi?id=MGI:96257) |
| EW1_F1plate01_C06 | AAH13060.1 | Bzw2 | [MGI:1914162](http://www.informatics.jax.org/searches/accession_report.cgi?id=MGI:1914162) | EW1_R1plate07_E02 | AAR16291.1 | met | [MGI:96969](http://www.informatics.jax.org/searches/accession_report.cgi?id=MGI:96969) |
| EW1_F1plate01_C07 | NP_727091.1 | G0030 | [FBgn0026708](http://flybase.bio.indiana.edu/.bin/fbidq.html?FBgn0026708) | EW1_R1plate07_E07 | AAH16523.1 | C130090K23Rik | [MGI:2444131](http://www.informatics.jax.org/searches/accession_report.cgi?id=MGI:2444131) |
| EW1_F1plate01_C07 | AAL39405.1 | CG3861 | [FBgn0029869](http://flybase.bio.indiana.edu/.bin/fbidq.html?FBgn0029869) | EW1_R1plate07_E10 | AAH10255.1 | Ptbp2 | [MGI:1860489](http://www.informatics.jax.org/searches/accession_report.cgi?id=MGI:1860489) |
| EW1_F1plate01_C07 | CAA83004.1 | cts-1 | [WBGene00000833](http://www.wormbase.org/db/gene/gene?class=CDS;name=WBGene00000833) | EW1_R1plate07_E12 | NP_647860.1 | Chd64 | [FBgn0035499](http://flybase.bio.indiana.edu/.bin/fbidq.html?FBgn0035499) |
| EW1_F1plate01_C10 | NP_573062.1 | G0136 | [FBgn0026666](http://flybase.bio.indiana.edu/.bin/fbidq.html?FBgn0026666) | EW1_R1plate07_E12 | AAH55338.1 | Tagln3 | [MGI:1926784](http://www.informatics.jax.org/searches/accession_report.cgi?id=MGI:1926784) |
| EW1_F1plate01_C10 | NP_081197.1 | Hbld2 | [MGI:1916296](http://www.informatics.jax.org/searches/accession_report.cgi?id=MGI:1916296) | EW1_R1plate07_F01 | AAI06185.1 | Pank2 | [MGI:1921700](http://www.informatics.jax.org/searches/accession_report.cgi?id=MGI:1921700) |
| EW1_F1plate01_C10 | XP_484225.2 | XM_484225 | [MGI:3574096](http://www.informatics.jax.org/searches/accession_report.cgi?id=MGI:3574096) | EW1_R1plate07_F01 | NP_076281.1 | Pank1 | [MGI:1922985](http://www.informatics.jax.org/searches/accession_report.cgi?id=MGI:1922985) |
| EW1_F1plate01_C10 | CAC51075.1 | Y39B6A.3 | [WBGene00012666](http://www.wormbase.org/db/gene/gene?class=CDS;name=WBGene00012666) | EW1_R1plate07_G03 | AAV36994.1 | 1810 | [FBgn0010497](http://flybase.bio.indiana.edu/.bin/fbidq.html?FBgn0010497) |
| EW1_F1plate01_D10 | AAV34801.1 | unc-89 | [WBGene00006820](http://www.wormbase.org/db/gene/gene?class=CDS;name=WBGene00006820) | EW1_R1plate07_G03 | NP_725600.1 | Picot | [FBgn0024315](http://flybase.bio.indiana.edu/.bin/fbidq.html?FBgn0024315) |
| EW1_F1plate01_E01 | NP_766194.2 | 6-Mar | [MGI:2442773](http://www.informatics.jax.org/searches/accession_report.cgi?id=MGI:2442773) | EW1_R1plate07_G03 | NP_609664.1 | CG9254 | [FBgn0028513](http://flybase.bio.indiana.edu/.bin/fbidq.html?FBgn0028513) |
| EW1_F1plate01_F03 | NP_071707.1 | Tnr | [MGI:99516](http://www.informatics.jax.org/searches/accession_report.cgi?id=MGI:99516) | EW1_R1plate07_G03 | NP_766361.1 | Slc17a5 | [MGI:1924105](http://www.informatics.jax.org/searches/accession_report.cgi?id=MGI:1924105) |
| EW1_F1plate01_F05 | NP_067617.2 | Agrin | [MGI:87961](http://www.informatics.jax.org/searches/accession_report.cgi?id=MGI:87961) | EW1_R1plate07_G07 | AAH28501.1 | Myg1 | [MGI:1929864](http://www.informatics.jax.org/searches/accession_report.cgi?id=MGI:1929864) |
| EW1_F1plate01_F05 | AAP13779.1 | F41G3.12 | [WBGene00018304](http://www.wormbase.org/db/gene/gene?class=CDS;name=WBGene00018304) | EW1_R1plate07_G08 | NP_001017427.1 | Rasef | [MGI:2448565](http://www.informatics.jax.org/searches/accession_report.cgi?id=MGI:2448565) |
| EW1_F1plate01_F11 | NP_067617.2 | Agrin | [MGI:87961](http://www.informatics.jax.org/searches/accession_report.cgi?id=MGI:87961) | EW1_R1plate07_G08 | NP_503120.1 | 4R79.2 | [WBGene00007067](http://www.wormbase.org/db/gene/gene?class=CDS;name=WBGene00007067) |
| EW1_F1plate01_F11 | AAP13779.1 | F41G3.12 | [WBGene00018304](http://www.wormbase.org/db/gene/gene?class=CDS;name=WBGene00018304) | EW1_R1plate07_H03 | AAH83109.1 | Ace | [MGI:87874](http://www.informatics.jax.org/searches/accession_report.cgi?id=MGI:87874) |
| EW1_F1plate01_F12 | NP_722724.1 | Eno | [FBgn0000579](http://flybase.bio.indiana.edu/.bin/fbidq.html?FBgn0000579) | EW1_R1plate07_H09 | NP_726105.1 | CG30290 | [FBgn0050290](http://flybase.bio.indiana.edu/.bin/fbidq.html?FBgn0050290) |
| EW1_F1plate01_G06 | NP_034210.1 | Dspp | [MGI:109172](http://www.informatics.jax.org/searches/accession_report.cgi?id=MGI:109172) | EW1_R1plate07_H09 | AAH52928.1 | Ppcdc | [MGI:1914062](http://www.informatics.jax.org/searches/accession_report.cgi?id=MGI:1914062) |
| EW1_F1plate01_G07 | AAH79896.1 | Tln2 | [MGI:1917799](http://www.informatics.jax.org/searches/accession_report.cgi?id=MGI:1917799) | EW1_R1Plate08_A03 | AAI08962.1 | Cyp2r1 | [MGI:2449771](http://www.informatics.jax.org/searches/accession_report.cgi?id=MGI:2449771) |
| EW1_F1plate01_G08 | AAK77295.1 | bt | [FBgn0005666](http://flybase.bio.indiana.edu/.bin/fbidq.html?FBgn0005666) | EW1_R1Plate08_A07 | AAH65084.1 | Mynn | [MGI:1931415](http://www.informatics.jax.org/searches/accession_report.cgi?id=MGI:1931415) |
| EW1_F1plate01_G08 | CAA98082.2 | unc-22 | [WBGene00006759](http://www.wormbase.org/db/gene/gene?class=CDS;name=WBGene00006759) | EW1_R1Plate08_B05 | CAA64892.1 | ahcY | [FBgn0014455](http://flybase.bio.indiana.edu/.bin/fbidq.html?FBgn0014455) |
| EW1_F1plate01_H02 | NP_034155.2 | Dcx | [MGI:1277171](http://www.informatics.jax.org/searches/accession_report.cgi?id=MGI:1277171) | EW1_R1Plate08_B05 | AAM27497.1 | Ahcy13 | [FBgn0014455](http://flybase.bio.indiana.edu/.bin/fbidq.html?FBgn0014455) |
| EW1_F1plate01_H09 | AAH14783.1 | Psmb3 | [MGI:1347014](http://www.informatics.jax.org/searches/accession_report.cgi?id=MGI:1347014) | EW1_R1Plate08_B05 | AAY55520.1 | alphaPS4 | [FBgn0034005](http://flybase.bio.indiana.edu/.bin/fbidq.html?FBgn0034005) |
| EW1_F1plate02_A02 | NP_511136.2 | fw | [FBgn0001083](http://flybase.bio.indiana.edu/.bin/fbidq.html?FBgn0001083) | EW1_R1Plate08_B10 | AAH61252.1 | Orc3l | [MGI:1354944](http://www.informatics.jax.org/searches/accession_report.cgi?id=MGI:1354944) |
| EW1_F1plate02_A02 | NP_573006.1 | CG9095 | [FBgn0030617](http://flybase.bio.indiana.edu/.bin/fbidq.html?FBgn0030617) | EW1_R1Plate08_D10 | NP_500207.1 | C45G7.2 | [WBGene00016669](http://www.wormbase.org/db/gene/gene?class=CDS;name=WBGene00016669) |
| EW1_F1plate02_A05 | AAH03310.1 | Commd7 | [MGI:1914197](http://www.informatics.jax.org/searches/accession_report.cgi?id=MGI:1914197) | EW1_R1Plate08_D10 | NP_500206.1 | C45G7.3 | [WBGene00016670](http://www.wormbase.org/db/gene/gene?class=CDS;name=WBGene00016670) |
| EW1_F1plate02_B02 | AAH55871.1 | Anxa4 | [MGI:88030](http://www.informatics.jax.org/searches/accession_report.cgi?id=MGI:88030) | EW1_R1Plate08_D10 | NP_501313.2 | C55F2.2 | [WBGene00016958](http://www.wormbase.org/db/gene/gene?class=CDS;name=WBGene00016958) |
| EW1_F1plate02_B05 | AAH03780.1 | Chi3l1 | [MGI:1340899](http://www.informatics.jax.org/searches/accession_report.cgi?id=MGI:1340899) | EW1_R1Plate08_D10 | AAA83197.1 | F22A3.6 | [WBGene00017691](http://www.wormbase.org/db/searches/basic?class=Any&query=WBGene00017691) |
| EW1_F1plate02_B05 | CAA63603.1 | brp39 | [MGI:1340899](http://www.informatics.jax.org/searches/accession_report.cgi?id=MGI:1340899) | EW1_R1Plate08_E02 | AAB59220.1 | N | [FBgn0004647](http://flybase.bio.indiana.edu/.bin/fbidq.html?FBgn0004647) |
| EW1_F1plate02_B07 | XP_289920.5 | Flna | [MGI:95556](http://www.informatics.jax.org/searches/accession_report.cgi?id=MGI:95556) | EW1_R1Plate08_E02 | CAB37610.1 | EG:140G11.1 | [FBgn0004647](http://flybase.bio.indiana.edu/.bin/fbidq.html?FBgn0004647) |
| EW1_F1plate02_D07 | NP_523783.1 | Gbp | [FBgn0013969](http://flybase.bio.indiana.edu/.bin/fbidq.html?FBgn0013969) | EW1_R1Plate08_E02 | AAB82004.1 | notch4 | [MGI:107471](http://www.informatics.jax.org/searches/accession_report.cgi?id=MGI:107471) |
| EW1_F1plate02_E08 | CAI25512.1 | Dusp14 | [MGI:1927168](http://www.informatics.jax.org/searches/accession_report.cgi?id=MGI:1927168) | EW1_R1Plate08_E02 | CAE48492.1 | Snep | [MGI:3045960](http://www.informatics.jax.org/searches/accession_report.cgi?id=MGI:3045960) |
| EW1_F1plate02_E08 | CAB03837.1 | C04F12 | [WBGene00004426](http://www.wormbase.org/db/gene/gene?class=CDS;name=WBGene00004426) | EW1_R1Plate08_H03 | AAH55291.1 | Car2 | [MGI:88269](http://www.informatics.jax.org/searches/accession_report.cgi?id=MGI:88269) |
| EW1_F1plate02_E12 | NP_731187.1 | CG31472 | [FBgn0051472](http://flybase.bio.indiana.edu/.bin/fbidq.html?FBgn0051472) | EW2_F1plate01_B03 | AAH16543.1 | Rnf126 | [MGI:1917544](http://www.informatics.jax.org/searches/accession_report.cgi?id=MGI:1917544) |
| EW1_F1plate02_E12 | AAH26564.1 | Pnpo | [MGI:2144151](http://www.informatics.jax.org/searches/accession_report.cgi?id=MGI:2144151) | EW2_F1plate01_D02 | NP_524525.3 | NepYr | [FBgn0004842](http://flybase.bio.indiana.edu/.bin/fbidq.html?FBgn0004842) |
| EW1_F1plate02_F03 | CAJ18497.1 | Gm2a | [MGI:95762](http://www.informatics.jax.org/searches/accession_report.cgi?id=MGI:95762) | EW2_F1plate01_D02 | AAO39845.1 | NPFR76F | [FBgn0036934](http://flybase.bio.indiana.edu/.bin/fbidq.html?FBgn0036934) |
| EW1_F1plate02_F09 | AAH53023.1 | Thbs3 | [MGI:98739](http://www.informatics.jax.org/searches/accession_report.cgi?id=MGI:98739) | EW2_F1plate01_D02 | NP_035065.1 | Npy6r | [MGI:1098590](http://www.informatics.jax.org/searches/accession_report.cgi?id=MGI:1098590) |
| EW1_F1plate02_F11 | AAH24078.1 | Ftcd | [MGI:1339962](http://www.informatics.jax.org/searches/accession_report.cgi?id=MGI:1339962) | EW2_F1plate01_D02 | AAT68889.1 | Y58G8A.4 | [WBGene00021983](http://www.wormbase.org/db/gene/gene?class=CDS;name=WBGene00021983) |
| EW1_F1plate02_F12 | AAH82790.1 | Cyc1 | [MGI:1913695](http://www.informatics.jax.org/searches/accession_report.cgi?id=MGI:1913695) | EW2_F1plate01_D07 | AAH58817.1 | Rps24 | [MGI:98147](http://www.informatics.jax.org/searches/accession_report.cgi?id=MGI:98147) |
| EW1_F1plate02_G01 | AAH83340.1 | Naca | [MGI:106095](http://www.informatics.jax.org/searches/accession_report.cgi?id=MGI:106095) | EW2_F1plate01_E02 | NP_727949.1 | CG9911 | [FBgn0030734](http://flybase.bio.indiana.edu/.bin/fbidq.html?FBgn0030734) |
| EW1_F1plate02_G06 | NP_082255.1 | Chit1 | [MGI:1919134](http://www.informatics.jax.org/searches/accession_report.cgi?id=MGI:1919134) | EW2_F1plate01_E02 | AAH19558.1 | Txndc4 | [MGI:1923549](http://www.informatics.jax.org/searches/accession_report.cgi?id=MGI:1923549) |
| EW1_F1plate02_G07 | CAA67981.1 | ATPsyn-d | [FBgn0016120](http://flybase.bio.indiana.edu/.bin/fbidq.html?FBgn0016120) | EW2_F1plate01_E07 | NP_523625.1 | Act42A | [FBgn0000043](http://flybase.bio.indiana.edu/.bin/fbidq.html?FBgn0000043) |
| EW1_F1plate02_G09 | AAH31986.1 | Arf1 | [MGI:99431](http://www.informatics.jax.org/searches/accession_report.cgi?id=MGI:99431) | EW2_F1plate01_F02 | NP_524003.1 | Uch-L3 | [FBgn0011327](http://flybase.bio.indiana.edu/.bin/fbidq.html?FBgn0011327) |
| EW1_F1plate02_H12 | NP_788553.1 | CG33054 | [FBgn0053054](http://flybase.bio.indiana.edu/.bin/fbidq.html?FBgn0053054) | EW2_F1plate01_F06 | AAB81971.1 | mopsm | [MGI:1858193](http://www.informatics.jax.org/searches/accession_report.cgi?id=MGI:1858193) |
| EW1_F1plate02_H12 | NP_997102.1 | AI314976 | [MGI:2146818](http://www.informatics.jax.org/searches/accession_report.cgi?id=MGI:2146818) | EW2_F1plate01_F06 | NP_058050.2 | Folh1 | [MGI:1858193](http://www.informatics.jax.org/searches/accession_report.cgi?id=MGI:1858193) |
| EW1_F1plate03_A09 | CAA98124.1 | lrp-1 | [WBGene00003071](http://www.wormbase.org/db/gene/gene?class=CDS;name=WBGene00003071) | EW2_F1plate01_G05 | NP_001027420.1 | CG30084 | [FBgn0083919](http://flybase.bio.indiana.edu/.bin/fbidq.html?FBgn0083919) |
| EW1_F1plate03_B11 | NP_082255.1 | Chit1 | [MGI:1919134](http://www.informatics.jax.org/searches/accession_report.cgi?id=MGI:1919134) | EW2_F1plate01_G07 | NP_610436.1 | CG11824 | [FBgn0033360](http://flybase.bio.indiana.edu/.bin/fbidq.html?FBgn0033360) |
| EW1_F1plate03_C01 | NP_032846.1 | Pepd | [MGI:97542](http://www.informatics.jax.org/searches/accession_report.cgi?id=MGI:97542) | EW2_F1plate02_A01 | NP_608658.1 | s5379 | [FBgn0010704](http://flybase.bio.indiana.edu/.bin/fbidq.html?FBgn0010704) |
| EW1_F1plate03_D11 | NP_524820.1 | net | [FBgn0002931](http://flybase.bio.indiana.edu/.bin/fbidq.html?FBgn0002931) | EW2_F1plate02_A01 | AAH51035.1 | Golph3 | [MGI:1913879](http://www.informatics.jax.org/searches/accession_report.cgi?id=MGI:1913879) |
| EW1_F1plate03_D11 | AAH23684.1 | Atoh8 | [MGI:1918343](http://www.informatics.jax.org/searches/accession_report.cgi?id=MGI:1918343) | EW2_F1plate02_A01 | AAF60665.1 | Y47G6A.18 | [WBGene00021644](http://www.wormbase.org/db/gene/gene?class=CDS;name=WBGene00021644) |
| EW1_F1plate03_E10 | AAH94900.1 | Hspa8 | [MGI:105384](http://www.informatics.jax.org/searches/accession_report.cgi?id=MGI:105384) | EW2_F1plate02_A07 | NP_996109.1 | Lmpt | [FBgn0036672](http://flybase.bio.indiana.edu/.bin/fbidq.html?FBgn0036672) |
| EW1_F1plate03_G07 | NP_001015410.1 | Cht3 | [FBgn0022701](http://flybase.bio.indiana.edu/.bin/fbidq.html?FBgn0022701) | EW2_F1plate02_B06 | NP_523507.2 | TepIII | [FBgn0041181](http://flybase.bio.indiana.edu/.bin/fbidq.html?FBgn0041181) |
| EW1_F1plate03_G07 | AAH03780.1 | Chi3l1 | [MGI:1340899](http://www.informatics.jax.org/searches/accession_report.cgi?id=MGI:1340899) | EW2_F1plate02_B06 | NP_694738.1 | Cd109 | [MGI:2445221](http://www.informatics.jax.org/searches/accession_report.cgi?id=MGI:2445221) |
| EW1_F1plate03_G07 | CAA63603.1 | brp39 | [MGI:1340899](http://www.informatics.jax.org/searches/accession_report.cgi?id=MGI:1340899) | EW2_F1plate02_B12 | AAR30199.1 | Ef1alpha48D | [FBgn0000556](http://flybase.bio.indiana.edu/.bin/fbidq.html?FBgn0000556) |
| EW1_F1plate03_G11 | NP_728275.1 | CG14207 | [FBgn0031037](http://flybase.bio.indiana.edu/.bin/fbidq.html?FBgn0031037) | EW2_F1plate02_B12 | AAT94431.1 | Ef1alpha100E | [FBgn0000557](http://flybase.bio.indiana.edu/.bin/fbidq.html?FBgn0000557) |
| EW1_F1plate03_H06 | NP_610454.2 | tsu | [FBgn0033378](http://flybase.bio.indiana.edu/.bin/fbidq.html?FBgn0033378) | EW2_F1plate02_B12 | CAA29994.1 | EF-1-alpha | [FBgn0000557](http://flybase.bio.indiana.edu/.bin/fbidq.html?FBgn0000557) |
| EW1_F1plate03_H06 | AAL48627.1 | CG8781 | [FBgn0033378](http://flybase.bio.indiana.edu/.bin/fbidq.html?FBgn0033378) | EW2_F1plate02_B12 | AAO21384.1 | eft-4 | [WBGene00001169](http://www.wormbase.org/db/gene/gene?class=CDS;name=WBGene00001169) |
| EW1_F1plate03_H06 | NP_080151.1 | Rbm8a | [MGI:1913129](http://www.informatics.jax.org/searches/accession_report.cgi?id=MGI:1913129) | EW2_F1plate02_C08 | AAH68193.1 | Rad23b | [MGI:105128](http://www.informatics.jax.org/searches/accession_report.cgi?id=MGI:105128) |
| EW1_F1plate03_H12 | NP_808331.1 | Isg20l2 | [MGI:2140076](http://www.informatics.jax.org/searches/accession_report.cgi?id=MGI:2140076) | EW2_F1plate02_C08 | CAA63146.1 | MHR23B | [MGI:105128](http://www.informatics.jax.org/searches/accession_report.cgi?id=MGI:105128) |
| EW1_F1plate04_A02 | AAL90369.1 | nahoda | [FBgn0034797](http://flybase.bio.indiana.edu/.bin/fbidq.html?FBgn0034797) | EW2_F1plate02_D09 | NP_733305.1 | ATPsyn-gamma | [FBgn0020235](http://flybase.bio.indiana.edu/.bin/fbidq.html?FBgn0020235) |
| EW1_F1plate04_A02 | NP_497053.1 | C09F9 | [WBGene00007479](http://www.wormbase.org/db/gene/gene?class=CDS;name=WBGene00007479) | EW2_F1plate02_G03 | NP_631883.2 | Ogt | [MGI:1339639](http://www.informatics.jax.org/searches/accession_report.cgi?id=MGI:1339639) |
| EW1_F1plate04_A08 | AAH25884.1 | Rpl35 | [MGI:1913739](http://www.informatics.jax.org/searches/accession_report.cgi?id=MGI:1913739) | EW2_F1plate03_A01 | CAD30668.1 | Grin2b | [MGI:95821](http://www.informatics.jax.org/searches/accession_report.cgi?id=MGI:95821) |
| EW1_F1plate04_A10 | NP_610269.1 | CG11107 | [FBgn0033160](http://flybase.bio.indiana.edu/.bin/fbidq.html?FBgn0033160) | EW2_F1plate03_A07 | NP_500207.1 | C45G7.2 | [WBGene00016669](http://www.wormbase.org/db/gene/gene?class=CDS;name=WBGene00016669) |
| EW1_F1plate04_A10 | AAC36129.1 | mDEAH9 | [MGI:1099786](http://www.informatics.jax.org/searches/accession_report.cgi?id=MGI:1099786) | EW2_F1plate03_A07 | NP_500206.1 | C45G7.3 | [WBGene00016670](http://www.wormbase.org/db/gene/gene?class=CDS;name=WBGene00016670) |
| EW1_F1plate04_B12 | NP_067617.2 | Agrin | [MGI:87961](http://www.informatics.jax.org/searches/accession_report.cgi?id=MGI:87961) | EW2_F1plate03_A07 | AAA83197.1 | F22A3.6 | [WBGene00017691](http://www.wormbase.org/db/searches/basic?class=Any&query=WBGene00017691) |
| EW1_F1plate04_B12 | AAP13779.1 | F41G3 | [WBGene00018304](http://www.wormbase.org/db/gene/gene?class=CDS;name=WBGene00018304) | EW2_F1plate03_B06 | AAK30001.1 | Rdh1 | [MGI:1195275](http://www.informatics.jax.org/searches/accession_report.cgi?id=MGI:1195275) |
| EW1_F1plate04_C01 | AAH99683.1 | Hnrpl | [MGI:104816](http://www.informatics.jax.org/searches/accession_report.cgi?id=MGI:104816) | EW2_F1plate03_B07 | CAJ18497.1 | Gm2a | [MGI:95762](http://www.informatics.jax.org/searches/accession_report.cgi?id=MGI:95762) |
| EW1_F1plate04_C04 | AAH60951.1 | Ndufab1 | [MGI:1917566](http://www.informatics.jax.org/searches/accession_report.cgi?id=MGI:1917566) | EW2_F1plate03_B09 | AAH29260.1 | Cul1 | [MGI:1349658](http://www.informatics.jax.org/searches/accession_report.cgi?id=MGI:1349658) |
| EW1_F1plate04_D05 | NP_610986.1 | CG10153 | [FBgn0033962](http://flybase.bio.indiana.edu/.bin/fbidq.html?FBgn0033962) | EW2_F1plate03_C05 | AAH24888.1 | Tmem66 | [MGI:1915137](http://www.informatics.jax.org/searches/accession_report.cgi?id=MGI:1915137) |
| EW1_F1plate04_D05 | NP_079977.2 | Trappc5 | [MGI:1913932](http://www.informatics.jax.org/searches/accession_report.cgi?id=MGI:1913932) | EW2_F1plate03_C07 | NP_032103.2 | Gad1 | [MGI:95632](http://www.informatics.jax.org/searches/accession_report.cgi?id=MGI:95632) |
| EW1_F1plate04_D05 | CAB55019.1 | Y57A10A | [WBGene00013267](http://www.wormbase.org/db/searches/basic?class=Any&query=WBGene00013267) | EW2_F1plate03_C09 | AAH29260.1 | Cul1 | [MGI:1349658](http://www.informatics.jax.org/searches/accession_report.cgi?id=MGI:1349658) |
| EW1_F1plate04_D12 | AAH30840.1 | Psmc5 | [MGI:105047](http://www.informatics.jax.org/searches/accession_report.cgi?id=MGI:105047) | EW2_F1plate03_C11 | NP_648985.1 | CG7580 | [FBgn0036728](http://flybase.bio.indiana.edu/.bin/fbidq.html?FBgn0036728) |
| EW1_F1plate04_E03 | AAH67063.1 | Ctsc | [MGI:109553](http://www.informatics.jax.org/searches/accession_report.cgi?id=MGI:109553) | EW2_F1plate03_D07 | AAH05547.1 | Tubb2c | [MGI:1915472](http://www.informatics.jax.org/searches/accession_report.cgi?id=MGI:1915472) |
| EW1_F1plate04_E04 | CAB65751.1 | TFF2/SP | [MGI:1306805](http://www.informatics.jax.org/searches/accession_report.cgi?id=MGI:1306805) | EW2_F1plate03_D07 | CAA86310.1 | tbb-4 | [WBGene00006538](http://www.wormbase.org/db/searches/basic?class=Any&query=WBGene00006538) |
| EW1_F1plate04_E04 | AAH50086.1 | Tff2 | [MGI:1306805](http://www.informatics.jax.org/searches/accession_report.cgi?id=MGI:1306805) | EW2_F1plate03_D09 | AAM50240.1 | CG15828 | [FBgn0032136](http://flybase.bio.indiana.edu/.bin/fbidq.html?FBgn0032136) |
| EW1_F1plate04_E06 | NP_731983.1 | Rpb7 | [FBgn0051155](http://flybase.bio.indiana.edu/.bin/fbidq.html?FBgn0051155) | EW2_F1plate03_E09 | AAH07152.1 | Eef2 | [MGI:95288](http://www.informatics.jax.org/searches/accession_report.cgi?id=MGI:95288) |
| EW1_F1plate04_E06 | NP_080605.1 | Polr2g | [MGI:1914960](http://www.informatics.jax.org/searches/accession_report.cgi?id=MGI:1914960) | EW2_F1plate03_F02 | NP_572306.1 | CG11700 | [FBgn0029856](http://flybase.bio.indiana.edu/.bin/fbidq.html?FBgn0029856) |
| EW1_F1plate04_E06 | AAF59550.1 | Y54E10BR | [WBGene00021845](http://www.wormbase.org/db/gene/gene?class=CDS;name=WBGene00021845) | EW2_F1plate03_F02 | AAH66197.1 | Ubb | [MGI:98888](http://www.informatics.jax.org/searches/accession_report.cgi?id=MGI:98888) |
| EW1_F1plate04_E12 | AAH32288.1 | Trhde | [MGI:2384311](http://www.informatics.jax.org/searches/accession_report.cgi?id=MGI:2384311) | EW2_F1plate03_F08 | NP_722724.1 | Eno | [FBgn0000579](http://flybase.bio.indiana.edu/.bin/fbidq.html?FBgn0000579) |
| EW1_F1plate04_F08 | NP_079615.1 | Tmem42 | [MGI:1277176](http://www.informatics.jax.org/searches/accession_report.cgi?id=MGI:1277176) | EW2_F1plate03_F09 | NP_001021590.1 | R11A5.4 | [WBGene00011232](http://www.wormbase.org/db/gene/gene?class=CDS;name=WBGene00011232) |
| EW1_F1plate04_F09 | AAH31986.1 | Arf1 | [MGI:99431](http://www.informatics.jax.org/searches/accession_report.cgi?id=MGI:99431) | EW2_F1plate03_G08 | AAH04745.1 | Tuba6 | [MGI:1095409](http://www.informatics.jax.org/searches/accession_report.cgi?id=MGI:1095409) |
| EW1_F1plate04_F10 | NP_079719.1 | Pno1 | [MGI:1913499](http://www.informatics.jax.org/searches/accession_report.cgi?id=MGI:1913499) | EW2_F1plate03_H02 | NP_001015410.1 | Cht3 | [FBgn0022701](http://flybase.bio.indiana.edu/.bin/fbidq.html?FBgn0022701) |
| EW1_F1plate04_G09 | AAL90369.1 | nahoda | [FBgn0034797](http://flybase.bio.indiana.edu/.bin/fbidq.html?FBgn0034797) | EW2_F1plate03_H02 | AAH03780.1 | Chi3l1 | [MGI:1340899](http://www.informatics.jax.org/searches/accession_report.cgi?id=MGI:1340899) |
| EW1_F1plate04_G09 | NP_497053.1 | C09F9 | [WBGene00007479](http://www.wormbase.org/db/gene/gene?class=CDS;name=WBGene00007479) | EW2_F1plate03_H02 | CAA63603.1 | brp39 | [MGI:1340899](http://www.informatics.jax.org/searches/accession_report.cgi?id=MGI:1340899) |
| EW1_F1plate04_H03 | AAH94900.1 | Hspa8 | [MGI:105384](http://www.informatics.jax.org/searches/accession_report.cgi?id=MGI:105384) | EW2_F1plate03_H05 | NP_722724.1 | Eno | [FBgn0000579](http://flybase.bio.indiana.edu/.bin/fbidq.html?FBgn0000579) |
| EW1_F1plate04_H08 | NP_082255.1 | Chit1 | [MGI:1919134](http://www.informatics.jax.org/searches/accession_report.cgi?id=MGI:1919134) | EW2_F1plate03_H07 | AAH86890.1 | Rpl30 | [MGI:98037](http://www.informatics.jax.org/searches/accession_report.cgi?id=MGI:98037) |
| EW1_F1plate05_A07 | AAH06019.1 | Sqstm1 | [MGI:107931](http://www.informatics.jax.org/searches/accession_report.cgi?id=MGI:107931) | EW2_F1plate03_H07 | CAA21573.1 | rpl-30 | [WBGene00004444](http://www.wormbase.org/db/searches/basic?class=Any&query=WBGene00004444) |
| EW1_F1plate05_A10 | AAO39563.1 | CG11255 | [FBgn0036337](http://flybase.bio.indiana.edu/.bin/fbidq.html?FBgn0036337) | EW2_F1plate03_H08 | AAS55697.1 | Casc1 | [MGI:2444480](http://www.informatics.jax.org/searches/accession_report.cgi?id=MGI:2444480) |
| EW1_F1plate05_B05 | NP_477418.1 | Taf10b | [FBgn0026324](http://flybase.bio.indiana.edu/.bin/fbidq.html?FBgn0026324) | EW2_R1plate01_A06 | AAF22221.1 | beta4GalT-III | [MGI:1928767](http://www.informatics.jax.org/searches/accession_report.cgi?id=MGI:1928767) |
| EW1_F1plate05_B05 | CAB59510.1 | TAFII30 | [MGI:1346320](http://www.informatics.jax.org/searches/accession_report.cgi?id=MGI:1346320) | EW2_R1plate01_A06 | AAH13619.1 | B4galt3 | [MGI:1928767](http://www.informatics.jax.org/searches/accession_report.cgi?id=MGI:1928767) |
| EW1_F1plate05_B07 | NP_611187.1 | CG6984 | [FBgn0034191](http://flybase.bio.indiana.edu/.bin/fbidq.html?FBgn0034191) | EW2_R1plate01_A08 | NP_731187.1 | CG31472 | [FBgn0051472](http://flybase.bio.indiana.edu/.bin/fbidq.html?FBgn0051472) |
| EW1_F1plate05_B07 | AAH02214.1 | Echdc3 | [MGI:1915106](http://www.informatics.jax.org/searches/accession_report.cgi?id=MGI:1915106) | EW2_R1plate01_A08 | AAH26564.1 | Pnpo | [MGI:2144151](http://www.informatics.jax.org/searches/accession_report.cgi?id=MGI:2144151) |
| EW1_F1plate05_B12 | AAH31986.1 | Arf1 | [MGI:99431](http://www.informatics.jax.org/searches/accession_report.cgi?id=MGI:99431) | EW2_R1plate01_A10 | NP_500207.1 | C45G7.2 | [WBGene00016669](http://www.wormbase.org/db/gene/gene?class=CDS;name=WBGene00016669) |
| EW1_F1plate05_C11 | NP_083572.1 | 1700001C19Rik | [MGI:1922712](http://www.informatics.jax.org/searches/accession_report.cgi?id=MGI:1922712) | EW2_R1plate01_C02 | AAK93412.1 | CG1869 | [FBgn0035398](http://flybase.bio.indiana.edu/.bin/fbidq.html?FBgn0035398) |
| EW1_F1plate05_D03 | NP_573314.1 | CG6540 | [FBgn0030943](http://flybase.bio.indiana.edu/.bin/fbidq.html?FBgn0030943) | EW2_R1plate01_D03 | AAA15214.1 | cbn | [FBgn0004580](http://flybase.bio.indiana.edu/.bin/fbidq.html?FBgn0004580) |
| EW1_F1plate05_D03 | NP_081367.1 | Nup35 | [MGI:1916732](http://www.informatics.jax.org/searches/accession_report.cgi?id=MGI:1916732) | EW2_R1plate01_E03 | AAH24078.1 | Ftcd | [MGI:1339962](http://www.informatics.jax.org/searches/accession_report.cgi?id=MGI:1339962) |
| EW1_F1plate05_D04 | AAH08241.1 | Psmb4 | [MGI:1098257](http://www.informatics.jax.org/searches/accession_report.cgi?id=MGI:1098257) | EW2_R1plate01_E04 | NP_741145.1 | mlc-3 | [WBGene00003371](http://www.wormbase.org/db/gene/gene?class=CDS;name=WBGene00003371) |
| EW1_F1plate05_D06 | AAH83327.1 | Rpl10 | [MGI:105943](http://www.informatics.jax.org/searches/accession_report.cgi?id=MGI:105943) | EW2_R1plate01_F09 | NP_476963.1 | RpL27A | [FBgn0010410](http://flybase.bio.indiana.edu/.bin/fbidq.html?FBgn0010410) |
| EW1_F1plate05_D12 | NP_291079.1 | Bcl3 | [MGI:88140](http://www.informatics.jax.org/searches/accession_report.cgi?id=MGI:88140) | EW2_R1plate01_F09 | AAH86939.1 | Rpl27a | [MGI:1347076](http://www.informatics.jax.org/searches/accession_report.cgi?id=MGI:1347076) |
| EW1_F1plate05_E04 | NP_573110.1 | CG3560 | [FBgn0030733](http://flybase.bio.indiana.edu/.bin/fbidq.html?FBgn0030733) | EW2_R1plate01_F10 | AAH49114.1 | Vcp | [MGI:99919](http://www.informatics.jax.org/searches/accession_report.cgi?id=MGI:99919) |
| EW1_F1plate05_E04 | NP_651614.1 | CG17856 | [FBgn0039576](http://flybase.bio.indiana.edu/.bin/fbidq.html?FBgn0039576) | EW2_R1plate01_F11 | AAH83344.1 | Tuba1 | [MGI:98869](http://www.informatics.jax.org/searches/accession_report.cgi?id=MGI:98869) |
| EW1_F1plate05_E04 | AAH86921.1 | Uqcrb | [MGI:1914780](http://www.informatics.jax.org/searches/accession_report.cgi?id=MGI:1914780) | EW2_R1plate01_H03 | AAG22803.1 | Gmfb | [MGI:1927133](http://www.informatics.jax.org/searches/accession_report.cgi?id=MGI:1927133) |
| EW1_F1plate05_E07 | NP_524741.1 | CanB | [FBgn0010014](http://flybase.bio.indiana.edu/.bin/fbidq.html?FBgn0010014) | EW2_R1plate01_H12 | NP_598839.2 | Chmp7 | [MGI:1913922](http://www.informatics.jax.org/searches/accession_report.cgi?id=MGI:1913922) |
| EW1_F1plate05_E07 | NP_524874.2 | CanB2 | [FBgn0015614](http://flybase.bio.indiana.edu/.bin/fbidq.html?FBgn0015614) | EW2_R1plate01_H12 | CAA92759.1 | T24B8.2 | [WBGene00011976](http://www.wormbase.org/db/gene/gene?name=WBGene00011976;class=Gene) |
| EW1_F1plate05_F01 | NP_032779.1 | Oaz1 | [MGI:109433](http://www.informatics.jax.org/searches/accession_report.cgi?id=MGI:109433) | EW2_R1plate02_D03 | CAA92745.1 | M7.1 - let-70 | [WBGene00002344](http://www.wormbase.org/db/searches/basic?class=Any&query=WBGene00002344) |
| EW1_F1plate05_F06 | NP_524714.1 | hoip | [FBgn0015393](http://flybase.bio.indiana.edu/.bin/fbidq.html?FBgn0015393) | EW2_R1plate02_D06 | AAH24666.1 | Cox6c | [MGI:104614](http://www.informatics.jax.org/searches/accession_report.cgi?id=MGI:104614) |
| EW1_F1plate05_F12 | AAS56006.1 | YDR174W | [SGD:S000002581](http://db.yeastgenome.org/cgi-bin/SGD/locus.pl?sgdid=S000002581) | EW2_R1plate02_E02 | AAH67063.1 | Ctsc | [MGI:109553](http://www.informatics.jax.org/searches/accession_report.cgi?id=MGI:109553) |
| EW1_F1plate05_F12 | AAS56006.1 | HMO1 | [SGD:S000002581](http://db.yeastgenome.org/cgi-bin/SGD/locus.pl?sgdid=S000002581) | EW2_R1plate02_F04 | NP_035290.1 | Prkg1 | [MGI:108174](http://www.informatics.jax.org/searches/accession_report.cgi?id=MGI:108174) |
| EW1_F1plate05_G04 | NP_724343.1 | His2A:CG31618 | [FBgn0051618](http://flybase.bio.indiana.edu/.bin/fbidq.html?FBgn0051618) | EW2_R1plate02_F05 | AAH25884.1 | Rpl35 | [MGI:1913739](http://www.informatics.jax.org/searches/accession_report.cgi?id=MGI:1913739) |
| EW1_F1plate05_G04 | NP_001027376.1 | His2A:CG33859 | [FBgn0053859](http://flybase.bio.indiana.edu/.bin/fbidq.html?FBgn0053859) | EW2_R1plate02_G02 | AAK21364.1 | ftn-2 | [WBGene00001501](http://www.wormbase.org/db/gene/gene?name=WBGene00001501;class=Gene) |
| EW1_F1plate05_H01 | NP_741145.1 | mlc-3 | [WBGene00003371](http://www.wormbase.org/db/gene/gene?class=CDS;name=WBGene00003371) | EW2_R1plate02_G03 | AAH94900.1 | Hspa8 | [MGI:105384](http://www.informatics.jax.org/searches/accession_report.cgi?id=MGI:105384) |
| EW1_F1plate06_A01 | AAO19472.1 | nucleostemin | [MGI:1353651](http://www.informatics.jax.org/searches/accession_report.cgi?id=MGI:1353651) | EW2_R1plate02_H03 | NP_082255.1 | Chit1 | [MGI:1919134](http://www.informatics.jax.org/searches/accession_report.cgi?id=MGI:1919134) |
| EW1_F1plate06_A01 | CAA88860.1 | K01C8.9 | [WBGene00003821](http://www.wormbase.org/db/searches/basic?class=Any&query=WBGene00003821) | EW2_R1plate03_A02 | CAD30668.1 | Grin2b | [MGI:95821](http://www.informatics.jax.org/searches/accession_report.cgi?id=MGI:95821) |
| EW1_F1plate06_A01 | CAA88860.1 | nst-1 | [WBGene00003821](http://www.wormbase.org/db/searches/basic?class=Any&query=WBGene00003821) | EW2_R1plate03_A10 | NP_871809.1 | carboxypeptidase | [WBGene00020281](http://www.wormbase.org/db/gene/gene?class=CDS;name=WBGene00020281) |
| EW1_F1plate06_A02 | CAB07240.1 | ran-1 | [WBGene00004302](http://www.wormbase.org/db/searches/basic?class=Any&query=WBGene00004302) | EW2_R1plate03_A12 | AAH31986.1 | Arf1 | [MGI:99431](http://www.informatics.jax.org/searches/accession_report.cgi?id=MGI:99431) |
| EW1_F1plate06_A03 | AAH66197.1 | Ubb | [MGI:98888](http://www.informatics.jax.org/searches/accession_report.cgi?id=MGI:98888) | EW2_R1plate03_B10 | NP_082255.1 | Chit1 | [MGI:1919134](http://www.informatics.jax.org/searches/accession_report.cgi?id=MGI:1919134) |
| EW1_F1plate06_A03 | AAH08661.1 | Ubc | [MGI:98889](http://www.informatics.jax.org/searches/accession_report.cgi?id=MGI:98889) | EW2_R1plate03_D02 | AAH82289.1 | Rpl13a | [MGI:1351455](http://www.informatics.jax.org/searches/accession_report.cgi?id=MGI:1351455) |
| EW1_F1plate06_A10 | NP_609792.1 | CaBP1 | [FBgn0025678](http://flybase.bio.indiana.edu/.bin/fbidq.html?FBgn0025678) | EW2_R1plate03_D04 | AAK68198.1 | C18E3.6 | [WBGene00015975](http://www.wormbase.org/db/gene/gene?class=CDS;name=WBGene00015975) |
| EW1_F1plate06_B04 | NP_523625.1 | Act42A | [FBgn0000043](http://flybase.bio.indiana.edu/.bin/fbidq.html?FBgn0000043) | EW2_R1plate03_D05 | NP_035864.2 | Siae | [MGI:104803](http://www.informatics.jax.org/searches/accession_report.cgi?id=MGI:104803) |
| EW1_F1plate06_B12 | NP_082255.1 | Chit1 | [MGI:1919134](http://www.informatics.jax.org/searches/accession_report.cgi?id=MGI:1919134) | EW2_R1plate03_F01 | NP_608575.1 | CG4764 | [FBgn0031310](http://flybase.bio.indiana.edu/.bin/fbidq.html?FBgn0031310) |
| EW1_F1plate06_D04 | NP_997151.1 | Ccdc87 | [MGI:3026882](http://www.informatics.jax.org/searches/accession_report.cgi?id=MGI:3026882) | EW2_R1plate03_F03 | AAH08997.1 | Anxa7 | [MGI:88031](http://www.informatics.jax.org/searches/accession_report.cgi?id=MGI:88031) |
| EW1_F1plate06_E05 | NP_080888.1 | Ndufb2 | [MGI:1915448](http://www.informatics.jax.org/searches/accession_report.cgi?id=MGI:1915448) | EW2_R1plate03_G04 | CAA91057.4 | ifa-3 | [WBGene00002051](http://www.wormbase.org/db/searches/basic?class=Any&query=WBGene00002051) |
| EW1_F1plate06_F02 | AAL28453.1 | Asph | [FBgn0034075](http://flybase.bio.indiana.edu/.bin/fbidq.html?FBgn0034075) | EW2_R1plate04_A03 | NP_511057.1 | sqh | [FBgn0003514](http://flybase.bio.indiana.edu/.bin/fbidq.html?FBgn0003514) |
| EW1_F1plate06_F03 | NP_741145.1 | mlc-3 | [WBGene00003371](http://www.wormbase.org/db/gene/gene?class=CDS;name=WBGene00003371) | EW2_R1plate04_A03 | AAH55439.1 | Myl9 | [MGI:2138915](http://www.informatics.jax.org/searches/accession_report.cgi?id=MGI:2138915) |
| EW1_F1plate06_F10 | NP_898911.1 | 2410018M08Rik | [MGI:1919220](http://www.informatics.jax.org/searches/accession_report.cgi?id=MGI:1919220) | EW2_R1plate04_A03 | CAA86772.1 | mlc-4 | [WBGene00003372](http://www.wormbase.org/db/searches/basic?class=Any&query=WBGene00003372) |
| EW1_F1plate06_H02 | NP_476969.1 | Vha14 | [FBgn0010426](http://flybase.bio.indiana.edu/.bin/fbidq.html?FBgn0010426) | EW2_R1plate04_A07 | CAA54318.1 | sod-1 | [WBGene00004930](http://www.wormbase.org/db/gene/gene?name=WBGene00004930;class=Gene) |
| EW1_F1plate06_H02 | AAH16553.1 | Atp6v1f | [MGI:1913394](http://www.informatics.jax.org/searches/accession_report.cgi?id=MGI:1913394) | EW2_R1plate04_B05 | AAH03780.1 | Chi3l1 | [MGI:1340899](http://www.informatics.jax.org/searches/accession_report.cgi?id=MGI:1340899) |
| EW1_F1plate06_H04 | NP_082255.1 | Chit1 | [MGI:1919134](http://www.informatics.jax.org/searches/accession_report.cgi?id=MGI:1919134) | EW2_R1plate04_B05 | CAA63603.1 | brp39 | [MGI:1340899](http://www.informatics.jax.org/searches/accession_report.cgi?id=MGI:1340899) |
| EW1_F1plate06_H06 | NP_035647.1 | Sycp3 | [MGI:109542](http://www.informatics.jax.org/searches/accession_report.cgi?id=MGI:109542) | EW2_R1plate04_B08 | CAD30668.1 | Grin2b | [MGI:95821](http://www.informatics.jax.org/searches/accession_report.cgi?id=MGI:95821) |
| EW1_F1plate07_A01 | NP_995711.1 | smi35A | [FBgn0016930](http://flybase.bio.indiana.edu/.bin/fbidq.html?FBgn0016930) | EW2_R1plate04_C03 | NP_728275.1 | CG14207 | [FBgn0031037](http://flybase.bio.indiana.edu/.bin/fbidq.html?FBgn0031037) |
| EW1_F1plate07_A01 | CAJ80825.1 | mbk-2 | [WBGene00003150](http://www.wormbase.org/db/searches/basic?class=Any&query=WBGene00003150) | EW2_R1plate04_C03 | AAO61437.1 | hsp-25 | [WBGene00002023](http://www.wormbase.org/db/gene/gene?class=CDS;name=WBGene00002023) |
| EW1_F1plate07_A05 | NP_610182.3 | l(2)09851 | [FBgn0022288](http://flybase.bio.indiana.edu/.bin/fbidq.html?FBgn0022288) | EW2_R1plate04_C07 | AAL48901.1 | CG7047 | [FBgn0035103](http://flybase.bio.indiana.edu/.bin/fbidq.html?FBgn0035103) |
| EW1_F1plate07_A05 | AAH83143.1 | Grwd1 | [MGI:2141989](http://www.informatics.jax.org/searches/accession_report.cgi?id=MGI:2141989) | EW2_R1plate04_C10 | NP_080145.1 | Dusp26 | [MGI:1914209](http://www.informatics.jax.org/searches/accession_report.cgi?id=MGI:1914209) |
| EW1_F1plate07_A07 | AAH54778.1 | Eftud2 | [MGI:1336880](http://www.informatics.jax.org/searches/accession_report.cgi?id=MGI:1336880) | EW2_R1plate04_D09 | NP_035864.2 | Siae | [MGI:104803](http://www.informatics.jax.org/searches/accession_report.cgi?id=MGI:104803) |
| EW1_F1plate07_B08 | NP_609792.1 | CaBP1 | [FBgn0025678](http://flybase.bio.indiana.edu/.bin/fbidq.html?FBgn0025678) | EW2_R1plate04_E12 | NP_731549.1 | RpL3 | [FBgn0020910](http://flybase.bio.indiana.edu/.bin/fbidq.html?FBgn0020910) |
| EW1_F1plate07_B12 | NP_572308.2 | Rpt4 | [FBgn0028685](http://flybase.bio.indiana.edu/.bin/fbidq.html?FBgn0028685) | EW2_R1plate04_G11 | NP_732311.1 | 14-3-3epsilon | [FBgn0020238](http://flybase.bio.indiana.edu/.bin/fbidq.html?FBgn0020238) |
| EW1_F1plate07_B12 | NP_648525.1 | CG7257 | [FBgn0036224](http://flybase.bio.indiana.edu/.bin/fbidq.html?FBgn0036224) | EW2_R1plate04_G12 | NP_511057.1 | sqh | [FBgn0003514](http://flybase.bio.indiana.edu/.bin/fbidq.html?FBgn0003514) |
| EW1_F1plate07_C07 | AAM11317.1 | cher | [FBgn0014141](http://flybase.bio.indiana.edu/.bin/fbidq.html?FBgn0014141) | EW2_R1plate04_G12 | AAH55439.1 | Myl9 | [MGI:2138915](http://www.informatics.jax.org/searches/accession_report.cgi?id=MGI:2138915) |
| EW1_F1plate07_C07 | AAH04061.1 | Flna | [MGI:95556](http://www.informatics.jax.org/searches/accession_report.cgi?id=MGI:95556) | EW2_R1plate04_G12 | CAA86772.1 | mlc-4 | [WBGene00003372](http://www.wormbase.org/db/searches/basic?class=Any&query=WBGene00003372) |
| EW1_F1plate07_E01 | NP_788664.2 | CG10126 | [FBgn0038088](http://flybase.bio.indiana.edu/.bin/fbidq.html?FBgn0038088) | EW2_R1plate05_B07 | NP_001033879.1 | CG9109 | [FBgn0031765](http://flybase.bio.indiana.edu/.bin/fbidq.html?FBgn0031765) |
| EW1_F1plate07_E01 | NP_731744.1 | CG31345 | [FBgn0051345](http://flybase.bio.indiana.edu/.bin/fbidq.html?FBgn0051345) | EW2_R1plate05_E01 | AAL29177.1 | CG2103 | [FBgn0035375](http://flybase.bio.indiana.edu/.bin/fbidq.html?FBgn0035375) |
| EW1_F1plate07_E08 | AAI08369.1 | Psma5 | [MGI:1347009](http://www.informatics.jax.org/searches/accession_report.cgi?id=MGI:1347009) | EW2_R1plate05_E06 | AAH07152.1 | Eef2 | [MGI:95288](http://www.informatics.jax.org/searches/accession_report.cgi?id=MGI:95288) |
| EW1_F1plate07_F07 | NP_728831.1 | CG32276 | [FBgn0047135](http://flybase.bio.indiana.edu/.bin/fbidq.html?FBgn0047135) | EW2_R1plate05_G02 | NP_511057.1 | sqh | [FBgn0003514](http://flybase.bio.indiana.edu/.bin/fbidq.html?FBgn0003514) |
| EW1_F1plate07_F07 | XP_899659.1 | 2810032E02Rik | [MGI:1919911](http://www.informatics.jax.org/searches/accession_report.cgi?id=MGI:1919911) | EW2_R1plate05_G02 | AAH55439.1 | Myl9 | [MGI:2138915](http://www.informatics.jax.org/searches/accession_report.cgi?id=MGI:2138915) |
| EW1_F1plate07_F07 | CAB03157.1 | F59F4 | [WBGene00009922](http://www.wormbase.org/db/searches/basic?class=Any&query=WBGene00009922) | EW2_R1plate05_G02 | CAA86772.1 | mlc-4 | [WBGene00003372](http://www.wormbase.org/db/searches/basic?class=Any&query=WBGene00003372) |
| EW1_F1plate07_F08 | AAR99111.1 | ATPsyn-b | [FBgn0019644](http://flybase.bio.indiana.edu/.bin/fbidq.html?FBgn0019644) | EW2_R1plate05_G03 | NP_609018.2 | CG9500 | [FBgn0031804](http://flybase.bio.indiana.edu/.bin/fbidq.html?FBgn0031804) |
| EW1_F1plate07_F08 | CAA86329.1 | asb-1 | [WBGene00000206](http://www.wormbase.org/db/searches/basic?class=Any&query=WBGene00000206) | EW2_R1plate05_G04 | NP_082255.1 | Chit1 | [MGI:1919134](http://www.informatics.jax.org/searches/accession_report.cgi?id=MGI:1919134) |
| EW1_F1plate07_F08 | AAA96210.1 | asb-2 | [WBGene00000207](http://www.wormbase.org/db/searches/basic?class=Any&query=WBGene00000207) | EW2_R1plate05_G05 | AAK21364.1 | ftn-2 | [WBGene00001501](http://www.wormbase.org/db/gene/gene?name=WBGene00001501;class=Gene) |
| EW1_F1plate07_G03 | AAA92327.1 | cpr-4 | [WBGene00000784](http://www.wormbase.org/db/gene/gene?class=CDS;name=WBGene00000784) | EW2_R1plate05_H01 | CAD30668.1 | Grin2b | [MGI:95821](http://www.informatics.jax.org/searches/accession_report.cgi?id=MGI:95821) |
| EW1_F1plate07_G05 | AAA15214.1 | cbn | [FBgn0004580](http://flybase.bio.indiana.edu/.bin/fbidq.html?FBgn0004580) | EW2_R1plate06_A01 | AAH86916.1 | Rpl31 | [MGI:2149632](http://www.informatics.jax.org/searches/accession_report.cgi?id=MGI:2149632) |
| EW1_F1plate07_H02 | AAH58382.1 | Rab2 | [MGI:1928750](http://www.informatics.jax.org/searches/accession_report.cgi?id=MGI:1928750) | EW2_R1plate06_A03 | NP_001014737.1 | up | [FBgn0004169](http://flybase.bio.indiana.edu/.bin/fbidq.html?FBgn0004169) |
| EW1_F1plate07_H02 | AAB52431.1 | unc-108 | [WBGene00006833](http://www.wormbase.org/db/gene/gene?class=CDS;name=WBGene00006833) | EW2_R1plate06_A03 | AAR24587.1 | TpnT | [FBgn0004169](http://flybase.bio.indiana.edu/.bin/fbidq.html?FBgn0004169) |
| EW1_F1plate07_H11 | AAH48073.1 | Ccdc65 | [MGI:2146001](http://www.informatics.jax.org/searches/accession_report.cgi?id=MGI:2146001) | EW2_R1plate06_A04 | NP_727486.1 | CG2061 | [FBgn0027498](http://flybase.bio.indiana.edu/.bin/fbidq.html?FBgn0027498) |
| EW1_F1plate08_A01 | NP_038853.1 | Ly75 | [MGI:106662](http://www.informatics.jax.org/searches/accession_report.cgi?id=MGI:106662) | EW2_R1plate06_A04 | NP_775590.1 | Lancl3 | [MGI:2443335](http://www.informatics.jax.org/searches/accession_report.cgi?id=MGI:2443335) |
| EW1_F1plate08_A01 | AAH27742.1 | Clec7a | [MGI:1861431](http://www.informatics.jax.org/searches/accession_report.cgi?id=MGI:1861431) | EW2_R1plate06_A07 | AAI00349.1 | Crim1 | [MGI:1354756](http://www.informatics.jax.org/searches/accession_report.cgi?id=MGI:1354756) |
| EW1_F1plate08_B05 | NP_611051.2 | CG8207 | [FBgn0034035](http://flybase.bio.indiana.edu/.bin/fbidq.html?FBgn0034035) | EW2_R1plate06_A07 | CAJ26240.1 | crm-1 | [WBGene00007103](http://www.wormbase.org/db/searches/basic?class=Any&query=WBGene00007103) |
| EW1_F1plate08_B05 | NP_598469.1 | Gmppa | [MGI:1916330](http://www.informatics.jax.org/searches/accession_report.cgi?id=MGI:1916330) | EW2_R1plate06_B03 | AAH03780.1 | Chi3l1 | [MGI:1340899](http://www.informatics.jax.org/searches/accession_report.cgi?id=MGI:1340899) |
| EW1_F1plate08_B06 | AAH82331.1 | Col2a1 | [MGI:88452](http://www.informatics.jax.org/searches/accession_report.cgi?id=MGI:88452) | EW2_R1plate06_B03 | CAA63603.1 | brp39 | [MGI:1340899](http://www.informatics.jax.org/searches/accession_report.cgi?id=MGI:1340899) |
| EW1_F1plate08_C02 | AAR30197.1 | CoVa | [FBgn0019624](http://flybase.bio.indiana.edu/.bin/fbidq.html?FBgn0019624) | EW2_R1plate06_D04 | AAH09167.1 | Sugt1 | [MGI:1915205](http://www.informatics.jax.org/searches/accession_report.cgi?id=MGI:1915205) |
| EW1_F1plate08_C02 | AAH34302.1 | Cox5a | [MGI:88474](http://www.informatics.jax.org/searches/accession_report.cgi?id=MGI:88474) | EW2_R1plate06_D08 | AAH86924.1 | Uba52 | [MGI:98887](http://www.informatics.jax.org/searches/accession_report.cgi?id=MGI:98887) |
| EW1_F1plate08_C10 | NP_492177.2 | R11A5.7 | [WBGene00011235](http://www.wormbase.org/db/gene/gene?class=CDS;name=WBGene00011235) | EW2_R1plate06_D08 | CAB04967.1 | ubq-2 | [WBGene00006728](http://www.wormbase.org/db/searches/basic?class=Any&query=WBGene00006728) |
| EW1_F1plate08_C10 | NP_871809.1 | carboxypeptidase | [WBGene00020281](http://www.wormbase.org/db/gene/gene?class=CDS;name=WBGene00020281) | EW2_R1plate06_D10 | CAA44197.1 | Epithelin | [MGI:95832](http://www.informatics.jax.org/searches/accession_report.cgi?id=MGI:95832) |
| EW1_F1plate08_C10 | NP_490776.2 | Y18H1A.9 | [WBGene00021213](http://www.wormbase.org/db/gene/gene?class=CDS;name=WBGene00021213) | EW2_R1plate06_E04 | NP_001014737.1 | up | [FBgn0004169](http://flybase.bio.indiana.edu/.bin/fbidq.html?FBgn0004169) |
| EW1_F1plate08_E04 | NP_082255.1 | Chit1 | [MGI:1919134](http://www.informatics.jax.org/searches/accession_report.cgi?id=MGI:1919134) | EW2_R1plate06_E04 | AAR24587.1 | TpnT | [FBgn0004169](http://flybase.bio.indiana.edu/.bin/fbidq.html?FBgn0004169) |
| EW1_F1plate08_E08 | AAB92035.1 | E04A4.7 | [WBGene00017121](http://www.wormbase.org/db/searches/basic?class=Any&query=WBGene00017121) | EW2_R1plate06_F05 | NP_080834.2 | 6720467C03Rik | [MGI:1915349](http://www.informatics.jax.org/searches/accession_report.cgi?id=MGI:1915349) |
| EW1_F1plate08_E10 | AAH33457.1 | Atp6v1d | [MGI:1921084](http://www.informatics.jax.org/searches/accession_report.cgi?id=MGI:1921084) | EW2_R1plate06_G03 | AAH66197.1 | Ubb | [MGI:98888](http://www.informatics.jax.org/searches/accession_report.cgi?id=MGI:98888) |
| EW1_F1plate08_E12 | NP_001027420.1 | CG30084 | [FBgn0083919](http://flybase.bio.indiana.edu/.bin/fbidq.html?FBgn0083919) | EW2_R1plate06_G06 | NP_500207.1 | C45G7.2 | [WBGene00016669](http://www.wormbase.org/db/gene/gene?class=CDS;name=WBGene00016669) |
| EW1_F1plate08_E12 | AAH04809.1 | Pdlim1 | [MGI:1860611](http://www.informatics.jax.org/searches/accession_report.cgi?id=MGI:1860611) | EW2_R1plate06_G06 | NP_500206.1 | C45G7.3 | [WBGene00016670](http://www.wormbase.org/db/gene/gene?class=CDS;name=WBGene00016670) |
| EW1_F1plate08_E12 | AAC08436.1 | Clim1 | [MGI:894670](http://www.informatics.jax.org/searches/accession_report.cgi?id=MGI:894670) | EW2_R1plate06_H11 | NP_506749.1 | cyn-7 | [WBGene00000883](http://www.wormbase.org/db/gene/gene?class=CDS;name=WBGene00000883) |
| EW1_F1plate08_H02 | AAH03930.1 | Dhrs1 | [MGI:1196314](http://www.informatics.jax.org/searches/accession_report.cgi?id=MGI:1196314) | EW2_R1plate07_C03 | NP_033104.1 | Rpl19 | [MGI:98020](http://www.informatics.jax.org/searches/accession_report.cgi?id=MGI:98020) |
| EW1_F1plate08_H02 | NP_498146.1 | dehydrogenase | [WBGene00000973](http://www.wormbase.org/db/gene/gene?class=CDS;name=WBGene00000973) | EW2_R1plate07_C05 | AAH49931.1 | Indo | [MGI:96416](http://www.informatics.jax.org/searches/accession_report.cgi?id=MGI:96416) |
| EW1_F1plate08_H10 | AAH46806.1 | Rpn2 | [MGI:98085](http://www.informatics.jax.org/searches/accession_report.cgi?id=MGI:98085) | EW2_R1plate07_C06 | NP_511117.1 | l(1)10Bb | [FBgn0001491](http://flybase.bio.indiana.edu/.bin/fbidq.html?FBgn0001491) |
| EW1_F1plate09_A04 | AAH30908.1 | Lrp2bp | [MGI:1914870](http://www.informatics.jax.org/searches/accession_report.cgi?id=MGI:1914870) | EW2_R1plate07_C06 | NP_499144.1 | C07A9.2 | [WBGene00007400](http://www.wormbase.org/db/gene/gene?class=CDS;name=WBGene00007400) |
| EW1_F1plate09_B01 | BAA78420.1 | mt3-mmp | [MGI:1276107](http://www.informatics.jax.org/searches/accession_report.cgi?id=MGI:1276107) | EW2_R1plate07_C07 | AAA15214.1 | cbn | [FBgn0004580](http://flybase.bio.indiana.edu/.bin/fbidq.html?FBgn0004580) |
| EW1_F1plate09_B01 | NP_062698.2 | Mmp16 | [MGI:1276107](http://www.informatics.jax.org/searches/accession_report.cgi?id=MGI:1276107) | EW2_R1plate07_C08 | NP_001013423.1 | D9Ertd402e | [MGI:1196217](http://www.informatics.jax.org/searches/accession_report.cgi?id=MGI:1196217) |
| EW1_F1plate09_B02 | NP_032478.1 | Klf2 | [MGI:1342772](http://www.informatics.jax.org/searches/accession_report.cgi?id=MGI:1342772) | EW2_R1plate07_C09 | NP_848783.3 | E430028B21Rik | [MGI:2443226](http://www.informatics.jax.org/searches/accession_report.cgi?id=MGI:2443226) |
| EW1_F1plate09_C05 | AAN71066.1 | MESK2 | [FBgn0043070](http://flybase.bio.indiana.edu/.bin/fbidq.html?FBgn0043070) | EW2_R1plate07_C10 | AAO39477.1 | CG8775 | [FBgn0038138](http://flybase.bio.indiana.edu/.bin/fbidq.html?FBgn0038138) |
| EW1_F1plate09_C07 | CAA38253.1 | USO1 | [SGD:S000002216](http://db.yeastgenome.org/cgi-bin/SGD/locus.pl?locus=S000002216) | EW2_R1plate07_C10 | NP_731787.1 | CG32473 | [FBgn0052473](http://flybase.bio.indiana.edu/.bin/fbidq.html?FBgn0052473) |
| EW1_F1plate09_D05 | AAR82779.1 | mask | [FBgn0043884](http://flybase.bio.indiana.edu/.bin/fbidq.html?FBgn0043884) | EW2_R1plate07_D08 | AAH56211.1 | Pafah1b2 | [MGI:108415](http://www.informatics.jax.org/searches/accession_report.cgi?id=MGI:108415) |
| EW1_F1plate09_D05 | NP_783598.1 | Ankrd5 | [MGI:2441685](http://www.informatics.jax.org/searches/accession_report.cgi?id=MGI:2441685) | EW2_R1plate07_D10 | AAH61154.1 | Chi3l3 | [MGI:1330860](http://www.informatics.jax.org/searches/accession_report.cgi?id=MGI:1330860) |
| EW1_F1plate09_D11 | AAL29139.1 | CG1597 | [FBgn0030289](http://flybase.bio.indiana.edu/.bin/fbidq.html?FBgn0030289) | EW2_R1plate07_D11 | AAH82790.1 | Cyc1 | [MGI:1913695](http://www.informatics.jax.org/searches/accession_report.cgi?id=MGI:1913695) |
| EW1_F1plate09_D11 | NP_502053.1 | F13H10.4 | [WBGene00008775](http://www.wormbase.org/db/gene/gene?class=CDS;name=WBGene00008775) | EW2_R1plate07_E02 | NP_648234.1 | CG6776 | [FBgn0035904](http://flybase.bio.indiana.edu/.bin/fbidq.html?FBgn0035904) |
| EW1_F1plate09_E03 | AAT94418.1 | oho23B | [FBgn0015521](http://flybase.bio.indiana.edu/.bin/fbidq.html?FBgn0015521) | EW2_R1plate07_E02 | AAL28890.2 | CG6673 | [FBgn0035906](http://flybase.bio.indiana.edu/.bin/fbidq.html?FBgn0035906) |
| EW1_F1plate09_E03 | AAH86912.1 | Rps21 | [MGI:1913731](http://www.informatics.jax.org/searches/accession_report.cgi?id=MGI:1913731) | EW2_R1plate07_E03 | NP_001014737.1 | up | [FBgn0004169](http://flybase.bio.indiana.edu/.bin/fbidq.html?FBgn0004169) |
| EW1_F1plate09_E05 | NP_727447.1 | Atg8a | [FBgn0052672](http://flybase.bio.indiana.edu/.bin/fbidq.html?FBgn0052672) | EW2_R1plate07_E03 | AAR24587.1 | TpnT | [FBgn0004169](http://flybase.bio.indiana.edu/.bin/fbidq.html?FBgn0004169) |
| EW1_F1plate09_E09 | AAZ67515.1 | CG4800 | [FBgn0037874](http://flybase.bio.indiana.edu/.bin/fbidq.html?FBgn0037874) | EW2_R1plate07_F02 | NP_524519.1 | His2Av | [FBgn0001197](http://flybase.bio.indiana.edu/.bin/fbidq.html?FBgn0001197) |
| EW1_F1plate09_F07 | NP_648603.1 | CG10754 | [FBgn0036314](http://flybase.bio.indiana.edu/.bin/fbidq.html?FBgn0036314) | EW2_R1plate07_G04 | AAH66197.1 | Ubb | [MGI:98888](http://www.informatics.jax.org/searches/accession_report.cgi?id=MGI:98888) |
| EW1_F1plate09_F07 | AAH52697.1 | Sf3a2 | [MGI:104912](http://www.informatics.jax.org/searches/accession_report.cgi?id=MGI:104912) | EW2_R1plate07_G04 | AAH08661.1 | Ubc | [MGI:98889](http://www.informatics.jax.org/searches/accession_report.cgi?id=MGI:98889) |
| EW1_F1plate09_F07 | CAA92593.2 | F11A10.2 | [WBGene00008683](http://www.wormbase.org/db/searches/basic?class=Any&query=WBGene00008683) | EW2_R1plate07_H01 | NP_083572.1 | 1700001C19Rik | [MGI:1922712](http://www.informatics.jax.org/searches/accession_report.cgi?id=MGI:1922712) |
| EW1_F1plate09_F11 | NP_649520.1 | CG10233 | [FBgn0037302](http://flybase.bio.indiana.edu/.bin/fbidq.html?FBgn0037302) | EW2_R1plate07_H05 | AAH34548.1 | Chia | [MGI:1932052](http://www.informatics.jax.org/searches/accession_report.cgi?id=MGI:1932052) |
| EW1_F1plate09_F11 | NP_932776.1 | BC023055 | [MGI:2449568](http://www.informatics.jax.org/searches/accession_report.cgi?id=MGI:2449568) | EW2_R1plate07_H06 | AAH51394.1 | Sart1 | [MGI:1309453](http://www.informatics.jax.org/searches/accession_report.cgi?id=MGI:1309453) |
| EW1_F1plate09_G03 | CAA44197.1 | Epithelin | [MGI:95832](http://www.informatics.jax.org/searches/accession_report.cgi?id=MGI:95832) | EW2_R1Plate08_A06 | NP_788751.1 | Ppn | [FBgn0003137](http://flybase.bio.indiana.edu/.bin/fbidq.html?FBgn0003137) |
| EW1_F1plate09_H05 | AAH36177.1 | 4930429A22Rik | [MGI:2442555](http://www.informatics.jax.org/searches/accession_report.cgi?id=MGI:2442555) | EW2_R1Plate08_A10 | AAH54768.1 | Sae2 | [MGI:1858313](http://www.informatics.jax.org/searches/accession_report.cgi?id=MGI:1858313) |
| EW1_F1plate09_H05 | AAC46543.1 | F48E8.6 | [WBGene00018612](http://www.wormbase.org/db/gene/gene?class=CDS;name=WBGene00018612) | EW2_R1Plate08_B07 | NP_082255.1 | Chit1 | [MGI:1919134](http://www.informatics.jax.org/searches/accession_report.cgi?id=MGI:1919134) |
| EW1_F1plate10_A11 | AAH20089.1 | Dcun1d5 | [MGI:1924113](http://www.informatics.jax.org/searches/accession_report.cgi?id=MGI:1924113) | EW2_R1Plate08_C04 | NP_609792.1 | CaBP1 | [FBgn0025678](http://flybase.bio.indiana.edu/.bin/fbidq.html?FBgn0025678) |
| EW1_F1plate10_A11 | NP_849227.1 | Dcun1d4 | [MGI:2140972](http://www.informatics.jax.org/searches/accession_report.cgi?id=MGI:2140972) | EW2_R1Plate08_C06 | CAA83432.1 | Cctz | [MGI:107184](http://www.informatics.jax.org/searches/accession_report.cgi?id=MGI:107184) |
| EW1_F1plate10_B01 | NP_031756.1 | Col12a1 | [MGI:88448](http://www.informatics.jax.org/searches/accession_report.cgi?id=MGI:88448) | EW2_R1Plate08_C08 | NP_598861.1 | D15Mgi27 | [MGI:2146241](http://www.informatics.jax.org/searches/accession_report.cgi?id=MGI:2146241) |
| EW1_F1plate10_B01 | NP_031764.1 | Col7a1 | [MGI:88462](http://www.informatics.jax.org/searches/accession_report.cgi?id=MGI:88462) | EW2_R1Plate08_C08 | AAF59574.3 | Y54G2A.4 | [WBGene00021870](http://www.wormbase.org/db/gene/gene?class=CDS;name=WBGene00021870) |
| EW1_F1plate10_C02 | NP_808238.1 | Slfnl1 | [MGI:3045330](http://www.informatics.jax.org/searches/accession_report.cgi?id=MGI:3045330) | EW2_R1Plate08_C09 | CAD30668.1 | Grin2b | [MGI:95821](http://www.informatics.jax.org/searches/accession_report.cgi?id=MGI:95821) |
| EW1_F1plate10_C02 | AAA96130.3 | F20A1.9 | [WBGene00017620](http://www.wormbase.org/db/gene/gene?class=CDS;name=WBGene00017620) | EW2_R1Plate08_D07 | AAH05779.1 | Sdhc | [MGI:1913302](http://www.informatics.jax.org/searches/accession_report.cgi?id=MGI:1913302) |
| EW1_F1plate10_C03 | AAY55322.1 | CG5197 | [FBgn0034147](http://flybase.bio.indiana.edu/.bin/fbidq.html?FBgn0034147) | EW2_R1Plate08_E10 | NP_608447.1 | Pros45 | [FBgn0020369](http://flybase.bio.indiana.edu/.bin/fbidq.html?FBgn0020369) |
| EW1_F1plate10_C03 | AAH61016.1 | 1700026D08Rik | [MGI:1922806](http://www.informatics.jax.org/searches/accession_report.cgi?id=MGI:1922806) | EW2_R1Plate08_E10 | NP_651811.1 | CG2241 | [FBgn0039788](http://flybase.bio.indiana.edu/.bin/fbidq.html?FBgn0039788) |
| EW1_F1plate10_C12 | AAH02054.1 | Scye1 | [MGI:102774](http://www.informatics.jax.org/searches/accession_report.cgi?id=MGI:102774) | EW2_R1Plate08_E10 | AAH04052.1 | Psmc5 | [MGI:105047](http://www.informatics.jax.org/searches/accession_report.cgi?id=MGI:105047) |
| EW1_F1plate10_D01 | XP_907379.1 | Rrbp1 | [MGI:1932395](http://www.informatics.jax.org/searches/accession_report.cgi?id=MGI:1932395) | EW2_R1Plate08_F03 | NP_871809.1 | carboxypeptidase | [WBGene00020281](http://www.wormbase.org/db/gene/gene?class=CDS;name=WBGene00020281) |
| EW1_F1plate10_E09 | AAA27909.2 | tyr-1 | [WBGene00015332](http://www.wormbase.org/db/gene/gene?class=CDS;name=WBGene00015332) | EW2_R1Plate08_F05 | BAA88309.1 | mDj11 | [MGI:1928373](http://www.informatics.jax.org/searches/accession_report.cgi?id=MGI:1928373) |
| EW1_F1plate10_E09 | AAK68589.4 | Y73B6BL.1 | [WBGene00022231](http://www.wormbase.org/db/gene/gene?class=CDS;name=WBGene00022231) | EW2_R1Plate08_F05 | AAH55729.1 | Dnajc7 | [MGI:1928373](http://www.informatics.jax.org/searches/accession_report.cgi?id=MGI:1928373) |
| EW1_F1plate10_F06 | NP_610545.1 | CG12129 | [FBgn0033475](http://flybase.bio.indiana.edu/.bin/fbidq.html?FBgn0033475) | EW2_R1Plate08_F10 | AAH54768.1 | Sae2 | [MGI:1858313](http://www.informatics.jax.org/searches/accession_report.cgi?id=MGI:1858313) |
| EW1_F1plate10_F06 | NP_081213.1 | Ascc1 | [MGI:1916340](http://www.informatics.jax.org/searches/accession_report.cgi?id=MGI:1916340) | EW2_R1Plate08_G05 | AAH83344.1 | Tuba1 | [MGI:98869](http://www.informatics.jax.org/searches/accession_report.cgi?id=MGI:98869) |
| EW1_F1plate10_G03 | CAA77110.1 | ETR-3 | [MGI:1338822](http://www.informatics.jax.org/searches/accession_report.cgi?id=MGI:1338822) | EW2_R1Plate08_G06 | NP_511057.1 | sqh | [FBgn0003514](http://flybase.bio.indiana.edu/.bin/fbidq.html?FBgn0003514) |
| EW1_F1plate10_G03 | NP_034290.1 | Cugbp2 | [MGI:1338822](http://www.informatics.jax.org/searches/accession_report.cgi?id=MGI:1338822) | EW2_R1Plate09_A05 | AAK93412.1 | CG1869 | [FBgn0035398](http://flybase.bio.indiana.edu/.bin/fbidq.html?FBgn0035398) |
| EW1_F2plate11_A09 | NP_034981.1 | Myd88 | [MGI:108005](http://www.informatics.jax.org/searches/accession_report.cgi?id=MGI:108005) | EW2_R1Plate09_A12 | AAH28865.1 | Bzw1 | [MGI:1914132](http://www.informatics.jax.org/searches/accession_report.cgi?id=MGI:1914132) |
| EW1_F2plate11_B12 | AAH83318.1 | Rpl5 | [MGI:102854](http://www.informatics.jax.org/searches/accession_report.cgi?id=MGI:102854) | EW2_R1Plate09_B02 | NP_608692.1 | CG3214 | [FBgn0031436](http://flybase.bio.indiana.edu/.bin/fbidq.html?FBgn0031436) |
| EW1_F2plate11_C07 | NP_033286.1 | Spnb2 | [MGI:98388](http://www.informatics.jax.org/searches/accession_report.cgi?id=MGI:98388) | EW2_R1Plate09_C02 | NP_500207.1 | C45G7.2 | [WBGene00016669](http://www.wormbase.org/db/gene/gene?class=CDS;name=WBGene00016669) |
| EW1_F2plate11_C12 | NP_608956.1 | CG11030 | [FBgn0031736](http://flybase.bio.indiana.edu/.bin/fbidq.html?FBgn0031736) | EW2_R1Plate09_C02 | NP_500206.1 | C45G7.3 | [WBGene00016670](http://www.wormbase.org/db/gene/gene?class=CDS;name=WBGene00016670) |
| EW1_F2plate11_C12 | NP_081166.1 | Ngdn | [MGI:1916216](http://www.informatics.jax.org/searches/accession_report.cgi?id=MGI:1916216) | EW2_R1Plate09_C05 | NP_035290.1 | Prkg1 | [MGI:108174](http://www.informatics.jax.org/searches/accession_report.cgi?id=MGI:108174) |
| EW1_F2plate11_D05 | CAA52784.1 | rpL19 | [FBgn0002607](http://flybase.bio.indiana.edu/.bin/fbidq.html?FBgn0002607) | EW2_R1Plate09_E03 | AAH94900.1 | Hspa8 | [MGI:105384](http://www.informatics.jax.org/searches/accession_report.cgi?id=MGI:105384) |
| EW1_F2plate11_D10 | AAH34113.1 | Trip12 | [MGI:1309481](http://www.informatics.jax.org/searches/accession_report.cgi?id=MGI:1309481) | EW2_R1Plate09_E04 | AAH52339.1 | Rpl7a | [MGI:1353472](http://www.informatics.jax.org/searches/accession_report.cgi?id=MGI:1353472) |
| EW1_F2plate11_D12 | NP_610409.1 | PGRP-SC1b | [FBgn0033327](http://flybase.bio.indiana.edu/.bin/fbidq.html?FBgn0033327) | EW2_R1Plate09_E05 | NP_033102.1 | Rpl12 | [MGI:98002](http://www.informatics.jax.org/searches/accession_report.cgi?id=MGI:98002) |
| EW1_F2plate11_D12 | NP_610410.1 | PGRP-SC2 | [FBgn0043575](http://flybase.bio.indiana.edu/.bin/fbidq.html?FBgn0043575) | EW2_R1Plate09_F02 | NP_001029047.1 | Ctrc | [MGI:1923951](http://www.informatics.jax.org/searches/accession_report.cgi?id=MGI:1923951) |
| EW1_F2plate11_E04 | AAT07066.1 | Adk | [MGI:87930](http://www.informatics.jax.org/searches/accession_report.cgi?id=MGI:87930) | EW2_R1Plate09_F02 | NP_079859.1 | Ctrb1 | [MGI:88559](http://www.informatics.jax.org/searches/accession_report.cgi?id=MGI:88559) |
| EW1_F2plate11_G01 | NP_609803.1 | CG17996 | [FBgn0032595](http://flybase.bio.indiana.edu/.bin/fbidq.html?FBgn0032595) | EW2_R1Plate09_F06 | AAA15214.1 | cbn | [FBgn0004580](http://flybase.bio.indiana.edu/.bin/fbidq.html?FBgn0004580) |
| EW1_F2plate11_G01 | AAH56653.1 | 2010110K16Rik | [MGI:1915149](http://www.informatics.jax.org/searches/accession_report.cgi?id=MGI:1915149) | EW2_R1Plate09_G06 | AAF26675.1 | Cpg16 | [MGI:1330861](http://www.informatics.jax.org/searches/accession_report.cgi?id=MGI:1330861) |
| EW1_F2plate11_G05 | AAH52169.1 | Tspan18 | [MGI:1917186](http://www.informatics.jax.org/searches/accession_report.cgi?id=MGI:1917186) | EW2_R1Plate10_A02 | AAH27212.1 | Cyb561d2 | [MGI:1929280](http://www.informatics.jax.org/searches/accession_report.cgi?id=MGI:1929280) |
| EW1_F2plate11_G07 | NP_666206.1 | Fads1 | [MGI:1923517](http://www.informatics.jax.org/searches/accession_report.cgi?id=MGI:1923517) | EW2_R1plate10_C02 | AAK21364.1 | ftn-2 | [WBGene00001501](http://www.wormbase.org/db/gene/gene?name=WBGene00001501;class=Gene) |
| EW1_F2plate12_B06 | AAM48331.1 | CG18589 | [FBgn0031931](http://flybase.bio.indiana.edu/.bin/fbidq.html?FBgn0031931) | EW2_R1Plate10_D02 | NP_036075.1 | Map3k1 | [MGI:1346872](http://www.informatics.jax.org/searches/accession_report.cgi?id=MGI:1346872) |
| EW1_F2plate12_B06 | NP_523507.2 | TepIII | [FBgn0041181](http://flybase.bio.indiana.edu/.bin/fbidq.html?FBgn0041181) | EW2_R1Plate10_D07 | NP_726435.1 | CG3376 | [FBgn0034997](http://flybase.bio.indiana.edu/.bin/fbidq.html?FBgn0034997) |
| EW1_F2plate12_B06 | CAB87809.1 | Tep3 | [FBgn0041181](http://flybase.bio.indiana.edu/.bin/fbidq.html?FBgn0041181) | EW2_R1Plate10_E06 | AAK93412.1 | CG1869 | [FBgn0035398](http://flybase.bio.indiana.edu/.bin/fbidq.html?FBgn0035398) |
| EW1_F2plate12_B06 | NP_694738.1 | Cd109 | [MGI:2445221](http://www.informatics.jax.org/searches/accession_report.cgi?id=MGI:2445221) | EW2_R1Plate10_F06 | CAA83432.1 | Cctz | [MGI:107184](http://www.informatics.jax.org/searches/accession_report.cgi?id=MGI:107184) |
| EW1_F2plate12_B08 | NP_079859.1 | Ctrb1 | [MGI:88559](http://www.informatics.jax.org/searches/accession_report.cgi?id=MGI:88559) | EW2_R1Plate10_G04 | NP_082255.1 | Chit1 | [MGI:1919134](http://www.informatics.jax.org/searches/accession_report.cgi?id=MGI:1919134) |
| EW1_F2plate12_C02 | AAH25571.1 | 2310005P05Rik | [MGI:1914734](http://www.informatics.jax.org/searches/accession_report.cgi?id=MGI:1914734) | EW2_R1Plate11_A12 | AAL90273.1 | Act57B | [FBgn0000044](http://flybase.bio.indiana.edu/.bin/fbidq.html?FBgn0000044) |
| EW1_F2plate12_C05 | NP_649058.1 | Nufip | [FBgn0036812](http://flybase.bio.indiana.edu/.bin/fbidq.html?FBgn0036812) | EW2_R1Plate11_B03 | NP_077754.2 | Lap3 | [MGI:1914238](http://www.informatics.jax.org/searches/accession_report.cgi?id=MGI:1914238) |
| EW1_F2plate12_C05 | AAL13717.1 | CG4076 | [FBgn0036812](http://flybase.bio.indiana.edu/.bin/fbidq.html?FBgn0036812) | EW2_R1Plate11_B09 | AAG22803.1 | Gmfb | [MGI:1927133](http://www.informatics.jax.org/searches/accession_report.cgi?id=MGI:1927133) |
| EW1_F2plate12_C05 | AAH56192.1 | Nufip1 | [MGI:1351474](http://www.informatics.jax.org/searches/accession_report.cgi?id=MGI:1351474) | EW2_R1Plate11_C05 | NP_082255.1 | Chit1 | [MGI:1919134](http://www.informatics.jax.org/searches/accession_report.cgi?id=MGI:1919134) |
| EW1_F2plate12_C08 | NP_081620.1 | 2310051M13Rik | [MGI:1917452](http://www.informatics.jax.org/searches/accession_report.cgi?id=MGI:1917452) | EW2_R1Plate11_C08 | NP_741145.1 | mlc-3 | [WBGene00003371](http://www.wormbase.org/db/gene/gene?class=CDS;name=WBGene00003371) |
| EW1_F2plate12_D07 | AAH60227.1 | Txlna | [MGI:105968](http://www.informatics.jax.org/searches/accession_report.cgi?id=MGI:105968) | EW2_R1Plate11_D08 | AAK93412.1 | CG1869 | [FBgn0035398](http://flybase.bio.indiana.edu/.bin/fbidq.html?FBgn0035398) |
| EW1_F2plate12_F12 | AAH03451.1 | Mat2a | [MGI:2443731](http://www.informatics.jax.org/searches/accession_report.cgi?id=MGI:2443731) | EW2_R1Plate11_D09 | AAH64781.1 | Gstp2 | [MGI:95864](http://www.informatics.jax.org/searches/accession_report.cgi?id=MGI:95864) |
| EW1_F2plate12_G02 | AAH63266.1 | Ints1 | [MGI:1915760](http://www.informatics.jax.org/searches/accession_report.cgi?id=MGI:1915760) | EW2_R1Plate11_D09 | AAH61109.1 | Gstp1 | [MGI:95865](http://www.informatics.jax.org/searches/accession_report.cgi?id=MGI:95865) |
| EW1_F2plate12_H08 | NP_666061.1 | Indol1 | [MGI:2142489](http://www.informatics.jax.org/searches/accession_report.cgi?id=MGI:2142489) | EW2_R1Plate11_E02 | AAH66197.1 | Ubb | [MGI:98888](http://www.informatics.jax.org/searches/accession_report.cgi?id=MGI:98888) |
| EW1_F2plate12_H08 | AAH49931.1 | Indo | [MGI:96416](http://www.informatics.jax.org/searches/accession_report.cgi?id=MGI:96416) | EW2_R1Plate11_E02 | AAH08661.1 | Ubc | [MGI:98889](http://www.informatics.jax.org/searches/accession_report.cgi?id=MGI:98889) |
| EW1_F2plate13_B01 | AAH11162.1 | Ncald | [MGI:1196326](http://www.informatics.jax.org/searches/accession_report.cgi?id=MGI:1196326) | EW2_R1Plate11_E04 | AAK93412.1 | CG1869 | [FBgn0035398](http://flybase.bio.indiana.edu/.bin/fbidq.html?FBgn0035398) |
| EW1_F2plate13_B04 | NP_608996.1 | Arc-p20 | [FBgn0031781](http://flybase.bio.indiana.edu/.bin/fbidq.html?FBgn0031781) | EW2_R1Plate11_E10 | NP_722637.1 | lwr | [FBgn0010602](http://flybase.bio.indiana.edu/.bin/fbidq.html?FBgn0010602) |
| EW1_F2plate13_B04 | AAH55309.1 | Arpc4 | [MGI:1915339](http://www.informatics.jax.org/searches/accession_report.cgi?id=MGI:1915339) | EW2_R1Plate11_F02 | AAK21364.1 | ftn-2 | [WBGene00001501](http://www.wormbase.org/db/gene/gene?name=WBGene00001501;class=Gene) |
| EW1_F2plate13_C03 | NP_725339.1 | shot | [FBgn0013733](http://flybase.bio.indiana.edu/.bin/fbidq.html?FBgn0013733) | EW2_R1Plate11_F03 | AAH86916.1 | Rpl31 | [MGI:2149632](http://www.informatics.jax.org/searches/accession_report.cgi?id=MGI:2149632) |
| EW1_F2plate13_D04 | NP_001014737.1 | up | [FBgn0004169](http://flybase.bio.indiana.edu/.bin/fbidq.html?FBgn0004169) | EW2_R1Plate11_F10 | AAH03441.1 | Arpc1b | [MGI:1343142](http://www.informatics.jax.org/searches/accession_report.cgi?id=MGI:1343142) |
| EW1_F2plate13_D04 | AAR24587.1 | TpnT | [FBgn0004169](http://flybase.bio.indiana.edu/.bin/fbidq.html?FBgn0004169) | EW2_R1Plate11_F10 | AAH01988.1 | Arpc1a | [MGI:1928896](http://www.informatics.jax.org/searches/accession_report.cgi?id=MGI:1928896) |
| EW1_F2plate13_D11 | AAH33449.1 | Rgs3 | [MGI:1354734](http://www.informatics.jax.org/searches/accession_report.cgi?id=MGI:1354734) | EW2_R1Plate11_G03 | AAL90369.1 | nahoda | [FBgn0034797](http://flybase.bio.indiana.edu/.bin/fbidq.html?FBgn0034797) |
| EW1_F2plate13_E09 | AAH26606.1 | Cdc16 | [MGI:1917207](http://www.informatics.jax.org/searches/accession_report.cgi?id=MGI:1917207) | EW2_R1Plate11_G03 | NP_497053.1 | C09F9.2 | [WBGene00007479](http://www.wormbase.org/db/gene/gene?class=CDS;name=WBGene00007479) |
| EW1_F2plate13_G03 | NP_062696.2 | ARL2 | [MGI:1928393](http://www.informatics.jax.org/searches/accession_report.cgi?id=MGI:1928393) | EW2_R1Plate11_G04 | AAV36984.1 | CG3662 | [FBgn0031285](http://flybase.bio.indiana.edu/.bin/fbidq.html?FBgn0031285) |
| EW2_R1Plate11_H04 | NP_733305.1 | ATPsyn-gamma | [FBgn0020235](http://flybase.bio.indiana.edu/.bin/fbidq.html?FBgn0020235) | EW2_R1Plate11_G08 | NP_032039.2 | Fgl2 | [MGI:103266](http://www.informatics.jax.org/searches/accession_report.cgi?id=MGI:103266) |
