# Supplementary material for: Cloning, analysis and functional annotation of expressed sequence tags from the Earthworm Eisenia fetida
Source: BMC Bioinformatics. 2007 Nov 1;8(Suppl 7):S7. doi: 10.1186/1471-2105-8-S7-S7 (PMC2099499; doi:10.1186/1471-2105-8-S7-S7)
Supplement: Additional file 3 — A complete listing of the KEGG pathways mapped for 157 unique Eisenia fetida sequences. [file 1471-2105-8-S7-S7-S3.doc]

| **KEGG Pathway** | **No. of Mapping** | **Sequence ID** | **No. of  Seq** | **Percentage of total** |
| --- | --- | --- | --- | --- |
| Carbohydrate Metabolism | **10** |  | 35 | 22% |
| Glycolysis / Gluconeogenesis |  | EW1_F1plate01_F12, EW1_F2Plate20_G03, EW1_R1plate02_G06, EW2_F1plate03_F08, EW2_F1plate03_H05 | 5 | 3% |
| Citrate cycle (TCA cycle) |  | EW1_F1plate01_C07, EW2_R1Plate08_D07 | 2 | 1% |
| Pentose and glucuronate interconversions |  | Contig18 | 1 | 1% |
| Fructose and mannose metabolism |  | EW1_F1plate08_B05, EW1_R1plate03_G02, EW1_R1plate05_H11, EW2_F1plate02_G03 | 4 | 3% |
| Starch and sucrose metabolism |  | EW1_F2Plate20_H09 | 1 | 1% |
| Aminosugars metabolism |  | Contig125, Contig269, Contig275, EW1_F1plate02_G06, EW1_F1plate03_B11, EW1_F1plate04_H08,  EW1_F1plate06_B12, EW1_F1plate06_H04, EW1_F1plate08_E04, EW1_R1plate06_B02, EW2_R1plate02_H03, EW2_R1plate03_B10, EW2_R1plate05_G04, EW2_R1plate07_H05, EW2_R1Plate08_B07, EW2_R1Plate10_G04, EW2_R1Plate11_C05 | 17 | 11% |
| Glyoxylate and dicarboxylate metabolism |  | EW1_F1plate01_C07 | 1 | 1% |
| Propanoate metabolism |  | EW1_F1plate05_B07, Contig321, EW1_R1plate03_D04 | 3 | 2% |
| Butanoate metabolism |  | EW2_F1plate03_C07, EW1_F1plate05_B07 | 2 | 1% |
| Inositol phosphate metabolism |  | EW1_F2plate14_A04 | 1 | 1% |
| Energy Metabolism | **8** |  | 28 | 18% |
| Oxidative phosphorylation |  | Contig10, Contig58, Contig65, EW1_F1plate01_B01, EW1_F1plate02_F12, EW1_F1plate02_G07, EW1_F1plate04_C04, EW1_F1plate05_E04, EW1_F1plate06_E05, EW1_F1plate06_H02, EW1_F1plate07_F08, EW1_F1plate08_C02, EW1_F1plate08_E10, EW1_F2Plate19_B05, EW1_F2Plate20_D05, EW1_R1plate05_E07, EW2_F1plate02_D09, EW2_R1plate02_D06, EW2_R1plate07_D11 | 19 | 12% |
| Sulfur metabolism |  | Contig163 | 1 | 1% |
| Fatty acid metabolism |  | EW1_F1plate05_B07, EW1_F2Plate20_G03, Contig321, EW1_R1plate03_D04 | 4 | 3% |
| Bile acid biosynthesis |  | EW1_F2Plate20_G03 | 1 | 1% |
| Glycerolipid metabolism |  | EW2_F1plate02_G03, EW1_R1plate01_C09 | 2 | 1% |
| Glycerophospholipid metabolism |  | EW1_R1plate01_C09 | 1 | 1% |
| Ether lipid metabolism |  | EW2_R1plate07_D08 | 1 | 1% |
| Arachidonic acid metabolism |  | Contig18 | 1 | 1% |
| Nucleotide Metabolism | **2** |  | 2 | 1% |
| Purine metabolism |  | EW1_F2plate11_E04, EW1_F1plate04_E06 | 2 | 1% |
| Pyrimidine metabolism |  | EW1_F1plate04_E06 | 1 | 1% |
| Amino Acid Metabolism | **12** |  | 18 | 11% |
| Glutamate metabolism |  | EW2_F1plate03_C07 | 1 | 1% |
| Alanine and aspartate metabolism |  | EW2_F1plate03_C07 | 1 | 1% |
| Glycine, serine and threonine metabolism |  | Contig278 | 1 | 1% |
| Methionine metabolism |  | Contig278, Contig206, EW1_F2plate12_F12, EW1_R1Plate08_B05 | 4 | 3% |
| Valine, leucine and isoleucine degradation |  | EW1_F1plate05_B07, Contig356, Contig321, EW1_R1plate03_D04 | 4 | 3% |
| Lysine degradation |  | EW1_F1plate05_B07 | 1 | 1% |
| Arginine and proline metabolism |  | Contig236, EW2_R1Plate11_B03 | 2 | 1% |
| Histidine metabolism |  | EW1_R1plate02_G06, EW1_F1plate02_F11, EW2_R1plate01_E03 | 3 | 2% |
| Tyrosine metabolism |  | EW1_F2Plate20_G03, EW1_R1plate02_G06, Contig356, EW1_R1plate05_C11 | 4 | 3% |
| Phenylalanine metabolism |  | EW1_R1plate02_G06, EW1_R1plate05_C11 | 2 | 1% |
| Tryptophan metabolism |  | EW1_F1plate05_B07, Contig356, EW1_F2plate12_H08, EW2_R1plate07_C05 | 4 | 3% |
| Phenylalanine, tyrosine and tryptophan biosynthesis |  | EW1_F1plate01_F12, EW2_F1plate03_F08, EW2_F1plate03_H05 | 3 | 2% |
| Metabolism of Other Amino Acids | **3** |  | 10 | 6% |
| beta-Alanine metabolism |  | EW2_F1plate03_C07, EW1_F1plate05_B07, Contig18, Contig321 | 4 | 3% |
| Selenoamino acid metabolism |  | Contig278, Contig206, EW1_F2plate12_F12, EW1_R1Plate08_B05, Contig163 | 5 | 3% |
| Glutathione metabolism |  | EW2_R1Plate11_D09 | 1 | 1% |
| Glycan Biosynthesis and Metabolism | **8** |  | 6 | 4% |
| N-Glycan biosynthesis |  | EW1_F1plate09_D11, EW2_R1plate01_A06 | 2 | 1% |
| N-Glycan degradation |  | EW1_R1plate03_C01, EW1_R1plate03_F10 | 2 | 1% |
| Keratan sulfate biosynthesis |  | EW2_R1plate01_A06 | 1 | 1% |
| Glycosphingolipid biosynthesis - neo-lactoseries |  | Contig64, EW2_R1plate01_A06 | 2 | 1% |
| Glycosphingolipid biosynthesis - globoseries |  | Contig64 | 1 | 1% |
| Glycan structures - biosynthesis 1 |  | EW1_F1plate09_D11, EW2_R1plate01_A06 | 2 | 1% |
| Glycan structures - biosynthesis 2 |  | EW2_R1plate01_A06, EW2_F1plate02_G03, Contig64 | 3 | 2% |
| Glycan structures - degradation |  | EW1_R1plate03_C01, EW1_R1plate03_F10 | 2 | 1% |
| Metabolism of Cofactors and Vitamins | **6** |  | 9 | 6% |
| Vitamin B6 metabolism |  | Contig356, EW1_F1plate02_E12, EW2_R1plate01_A08 | 3 | 2% |
| Nicotinate and nicotinamide metabolism |  | Contig356 | 1 | 1% |
| Pantothenate and CoA biosynthesis |  | EW1_R1plate07_F01, EW1_R1plate07_H09 | 2 | 1% |
| Folate biosynthesis |  | EW1_F2Plate20_H09 | 1 | 1% |
| One carbon pool by folate |  | EW1_F1plate02_F11, EW2_R1plate01_E03 | 2 | 1% |
| Retinol metabolism |  | EW1_R1plate02_G06 | 1 | 1% |
| Biosynthesis of Secondary Metabolites | **1** |  | 2 | 1% |
| Limonene and pinene degradation |  | EW1_F1plate05_B07, EW1_F1plate08_H02 | 2 | 1% |
| Xenobiotics Biodegradation and Metabolism | **7** |  | 6 | 4% |
| Caprolactam degradation |  | EW1_F1plate05_B07 | 1 | 1% |
| gamma-Hexachlorocyclohexane degradation |  | EW1_F1plate08_H02 | 1 | 1% |
| Ethylbenzene degradation |  | EW2_R1plate07_D08 | 1 | 1% |
| Benzoate degradation via CoA ligation |  | EW1_F1plate05_B07, EW1_F1plate08_H02 | 2 | 1% |
| Bisphenol A degradation |  | EW1_F1plate08_H02 | 1 | 1% |
| 1- and 2-Methylnaphthalene degradation |  | EW1_F1plate08_H02, EW1_F2Plate20_G03 | 2 | 1% |
| Metabolism of xenobiotics by cytochrome P450 |  | EW1_F2Plate20_G03, EW1_R1plate02_G06, EW2_R1Plate11_D09 | 3 | 2% |
| Transcription | **2** |  | 2 | 1% |
| RNA polymerase |  | EW1_F1plate04_E06 | 1 | 1% |
| Basal transcription factors |  | EW1_F1plate05_B05 | 1 | 1% |
| Translation | **1** |  | 17 | 11% |
| Ribosome |  | Contig164, Contig201, Contig312, Contig385, Contig78, EW1_F1plate09_E03, EW1_F2plate16_A07, EW1_F2plate16_A08, EW1_F2plate16_A09, EW1_F2plate16_A10, EW1_F2plate16_A11, EW1_F2plate16_A12, EW2_F1plate01_D07, EW2_F1plate03_H07, EW2_R1plate01_F09, EW2_R1plate03_D02, EW2_R1plate07_C03 | 17 | 11% |
| Folding, Sorting and Degradation | **3** |  | 9 | 6% |
| Ubiquitin mediated proteolysis |  | EW2_R1plate02_D03 | 1 | 1% |
| Proteasome |  | Contig292, EW1_F1plate01_H09, EW1_F1plate04_D12, EW1_F1plate05_D04, EW1_F1plate07_B12, EW1_F1plate07_E08, EW2_R1Plate08_E10 | 7 | 4% |
| DNA polymerase |  | Contig52 | 1 | 1% |
| Membrane Transport | **1** |  | 1 | 1% |
| ABC transporters - General |  | Contig66 | 1 | 1% |
| Signal Transduction | **6** |  | 14 | 9% |
| MAPK signaling pathway |  | EW1_F1plate02_B07, EW1_F1plate07_C07, EW1_F1plate02_E08, EW2_R1Plate10_D02, EW1_R1plate04_D09 | 5 | 3% |
| Wnt signaling pathway |  | EW1_F1plate05_E07, EW2_F1plate03_B09, EW2_F1plate03_C09 | 3 | 2% |
| Notch signaling pathway |  | EW1_R1Plate08_E02, Contig116, EW1_R1plate03_B09 | 3 | 2% |
| TGF-beta signaling pathway |  | EW1_F1plate02_F09, EW2_F1plate03_B09, EW2_F1plate03_C09 | 3 | 2% |
| Calcium signaling pathway |  | Contig215 | 1 | 1% |
| Phosphatidylinositol signaling system |  | Contig215, EW1_R1plate01_C09 | 2 | 1% |
| Signaling Molecules and Interaction | **3** |  | 13 | 8% |
| Neuroactive ligand-receptor interaction |  | EW2_F1plate03_A01, EW2_R1plate03_A02, EW2_R1plate04_B08, EW2_R1plate05_H01, EW2_R1Plate08_C09, EW2_F1plate01_D02 | 6 | 4% |
| Cytokine-cytokine receptor interaction |  | EW1_R1plate07_E02 | 1 | 1% |
| ECM-receptor interaction |  | EW1_F1plate01_F05, EW1_F1plate01_F11, EW1_F1plate04_B12, EW1_F1plate01_F03, EW1_F1plate02_F09, EW1_F1plate08_B06 | 6 | 4% |
| Cell Motility | **3** |  | 9 | 6% |
| Regulation of actin cytoskeleton |  | EW1_F2plate13_B04, EW1_F2Plate20_C02, EW2_F1plate01_E07, EW2_R1Plate11_A12, EW2_R1Plate11_F10 | 5 | 3% |
| Cell cycle |  | EW1_F2plate13_E09, EW2_F1plate03_B09, EW2_F1plate03_C09 | 3 | 2% |
| Apoptosis |  | EW1_F2plate11_A09 | 1 | 1% |
| Cell Communication | **4** |  | 13 | 8% |
| Focal adhesion |  | EW1_R1plate07_E02, EW1_F1plate01_F03, EW1_F1plate02_B07, EW1_F1plate02_F09, EW1_F1plate07_C07, EW1_F1plate08_B06 | 6 | 4% |
| Adherens junction |  | EW1_R1plate07_E02 | 1 | 1% |
| Tight junction |  | Contig158, EW1_F2plate11_C07 | 2 | 1% |
| Gap junction |  | EW2_F1plate03_D07, EW2_R1plate01_F11, EW2_R1plate02_F04, EW2_R1Plate08_G05, EW2_R1Plate09_C05 | 5 | 3% |
| Endocrine System | **3** |  | 4 | 3% |
| Insulin signaling pathway |  | Contig215 | 1 | 1% |
| PPAR signaling pathway |  | Contig321, EW1_R1plate03_D04 | 2 | 1% |
| GnRH signaling pathway |  | Contig215, EW2_R1Plate10_D02 | 2 | 1% |
| Immune System | **3** |  | 5 | 3% |
| Complement and coagulation cascades |  | Contig214 | 1 | 1% |
| Toll-like receptor signaling pathway |  | EW1_F2plate11_A09 | 1 | 1% |
| Antigen processing and presentation |  | Contig178, Contig363, EW1_R1plate04_D09 | 3 | 2% |
| Nervous System | **2** |  | 8 | 5% |
| Long-term potentiation |  | Contig215, EW2_F1plate03_A01, EW2_R1plate03_A02, EW2_R1plate04_B08, EW2_R1plate05_H01, EW2_R1Plate08_C09 | 6 | 4% |
| Long-term depression |  | EW2_R1plate02_F04, EW2_R1Plate09_C05 | 2 | 1% |
| Sensory System | **1** |  | 3 | 2% |
| Olfactory transduction |  | EW2_R1plate02_F04, EW2_R1Plate09_C05, Contig215 | 3 | 2% |
| Development | **2** |  | 3 | 2% |
| Dorso-ventral axis formation |  | EW1_R1Plate08_E02 | 1 | 1% |
| Axon guidance |  | EW1_F2plate13_D11, EW1_R1plate07_E02 | 2 | 1% |
| Neurodegenerative Disorders | **4** |  | 6 | 4% |
| Alzheimer's disease |  | Contig116, EW1_R1plate03_B09 | 2 | 1% |
| Parkinson's disease |  | EW1_F2plate16_B03 | 1 | 1% |
| Huntington's disease |  | Contig278, Contig215 | 2 | 1% |
| Prion disease |  | EW1_R1plate04_D09 | 1 | 1% |
| Metabolic Disorders | **2** |  | 2 | 1% |
| Type II diabetes mellitus |  | EW2_F1plate03_C07 | 1 | 1% |
| Maturity onset diabetes of the young |  | EW1_F2plate14_D06 | 1 | 1% |
| Cancers | **2** |  | 2 | 1% |
| Colorectal cancer |  | EW1_R1plate07_E02 | 1 | 1% |
| Glioma |  | Contig215 | 1 | 1% |
